# Application of modular isoxazoline-β^2,2^-amino acid-based peptidomimetics as chemical model systems for studying the tau misfolding

**DOI:** 10.1016/j.isci.2025.112272

**Published:** 2025-03-22

**Authors:** Davide di Lorenzo, Nicolo Bisi, Raffaella Bucci, Inga Ennen, Leonardo Lo Presti, Veronica Dodero, Roland Brandt, Sandrine Ongeri, Maria-Luisa Gelmi, Nicolo Tonali

**Affiliations:** 1 Université Paris-Saclay, CNRS, BioCIS, Bat. Henri Moissan, 17 av. des Sciences, 91400 Orsay, France; 2 Dipartimento di Scienze Farmaceutiche, DISFARM, Università degli Studi di Milano, Via Venezian 21, 20133 Milano, Italy; 3 Department of Neurobiology, Osnabrück University, Barbarastrasse 11, D-49076 Osnabrück, Germany; 4 Department of Physics, Bielefeld University Universitätsstr. 25, 33615 Bielefeld, Germany; 5 Department of Chemistry, Università degli Studi di Milano, Via Golgi 19, 20133 Milano, Italy; 6 Department of Chemistry, Physical and Biophysical Chemistry, Bielefeld University Universitätsstr. 25, 33615 Bielefeld, Germany; 7 CEA Saclay, DRF/JOLIOT/DMTS/SIMoS/LPEM, 91191 Gif-sur-Yvette, France

**Keywords:** Biochemistry, Molecular biology, Structural biology

## Abstract

Tau is a microtubule-associated protein essential for regulating microtubule dynamics and axonal transport in neurons. In tauopathies, the transition of tau from a physiological to a pathological form remains unclear, though the hexapeptides PHF6 and PHF6∗ are key in triggering aggregation. These sequences are shielded by a β-hairpin structure in the native state but expose hydrophobic residues during misfolding, promoting self-assembly. This study employs a non-natural β^2^-amino acid to induce PHF6 and PHF6∗ into either extended or β-hairpin conformations. The extended form triggers tau aggregation without additives, acting as a seed-competent monomer model system. Conversely, the β-hairpin preserves tau in a soluble monomeric state. Additionally, a β-hairpin mimic inspired by Hsp90 showed potential as a chaperone mimic and inhibitor of tau aggregation, offering insights into corrective folding and aggregation modulation in neuronal environments.

## Highlights

• β^2,2^-amino acid–based peptides modulate tau misfolding according to the conformation
• The seed-mimic’s extended form triggers tau aggregation, forming fibrils with unique traits
• Fibrils obtained with the seed-mimic are closer to those found in patients’ brains
• Mimics of Hsp90 inhibit tau aggregation and act as chaperones for new therapies

## Graphical abstract

**Figure:**
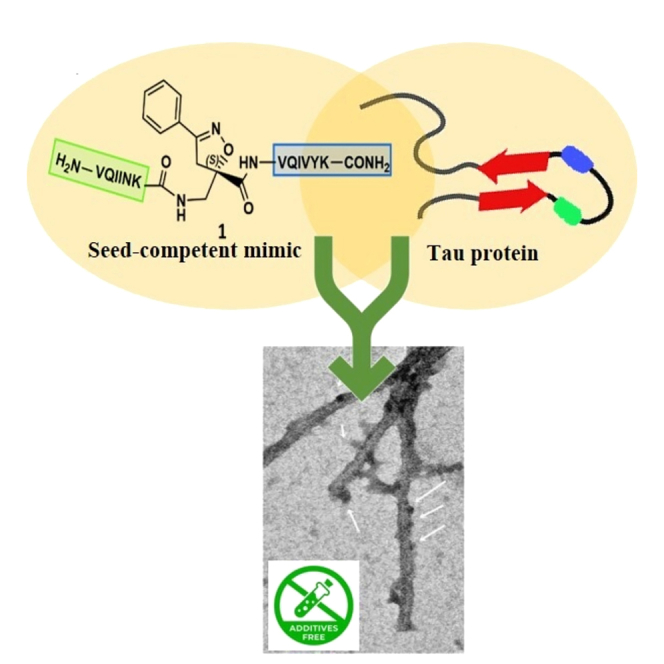

## Introduction

Tauopathies are a group of neurodegenerative diseases characterized by the deposition of abnormal aggregates of tau protein in the brain, forming neurofibrillary tangles (NFTs) inside the neuronal cells.^1^ Tau is a microtubule (MT)-associated protein (MAP) whose physiological role is to regulate MT polymerization and ensure the correct axonal transport in neurons.^2^ Tau interacts with MTs through the microtubule-binding domain (MTBD)(Q244-K370) that differs, among the various tau isoforms, in terms of the repeat domain numbers (three, 3R, to four, 4R).^3^ The 4R isoform was shown to promote microtubule assembly and binding to microtubules better than the 3R isoform.^4^ However, in tauopathies, tau undergoes a conformational transition from a physiological to a pathological folding state, the mechanism of which is still unclear.^5^ It is widely accepted that conformational or post-translational changes in the protein must initiate the autocatalytic aggregation. Furthermore, this self-assembly, associated with abnormal phosphorylation, induces the detachment from MTs and the formation of intracellular aggregates.^6^ This process is structurally unique for each tau strain and tauopathy, resulting in a different panel of fibril structures and polymorphs.^7^^,^^8^

**Figure 1:**
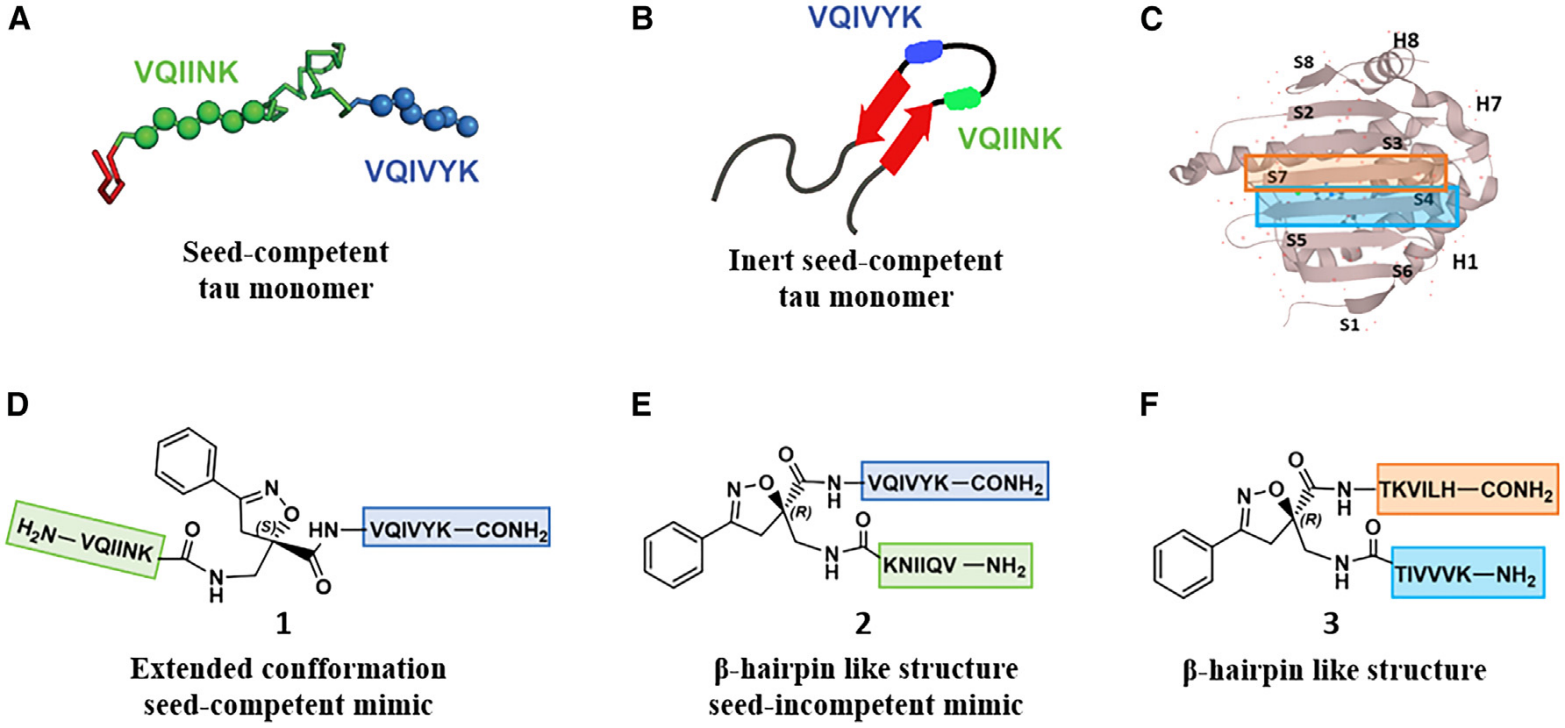
Schematic representation of the designed compounds 1, 2, and 3 (A) conformation of tau monomer in the seed component organization as reported by Mirbaha et al.^10^ and (B) conformation of tau monomer in the inert seed-component organization, as reported by Fernandez-Ramirez et al.^11^; highlighted in green PHF6∗, (VQIINK) and blue PHF6, (VQIVYK). (C) Three-dimensional conformation of the crystal structure of the N-terminal portion of Hsp90 (PDB = 3NMQ). The S4 and S7 β-sheet in an antiparallel position are highlighted in blue and orange, respectively; (D) Chemical structure of extended peptide **1** bearing the S-configured Isox-β^2^-AA which is based on seed-competent tau monomer shown in (A). (E) Chemical structure of β-hairpin peptide **2** bearing the R-configured Isox-β^2^-AA which is based on inert seed-competent tau monomer shown in B and according to the hairpin shape of Kadavath et al.^3^ and Fernandez-Ramirez et al.^11^ (F) Chemical structure of β-hairpin peptide **3** bearing the R-configured Isox-β^2^-AA which is based on the N-terminal portion core of Hsp90 (PDB = 3NMQ) shown in (C).

Morphologically, NTFs can be composed of two types of fibrils: paired helical filaments (PHFs) and straight filaments (SFs), whose common structural core consists of residues V306-F378, indicating the inclusion of R3 and R4 repeats.^7^ R2 has been demonstrated not to be part of this pronase-resistant core, as immunolabeling of PHFs and SFs from brain patients with an R2-specific epitope antibody (anti-4R, raised against V275-C291 of R2) after pronase treatment was negative.^7^ It is evident that the MTBD plays a central role in tau protein aggregation. Particularly, tau aggregation seems to be mediated by short motifs, called amyloid motifs, corresponding to highly hydrophobic regions of the full sequence. Two hexapeptide sequences, named PHF6 and PHF6∗, which are located in the R3 and R2 repetition domains, respectively, seem to be crucial in triggering the aggregation (Figures 1A and 1B). The hexapeptide PHF6 (^306^VQIVYK^311^) has been confirmed, by cryo-EM analysis, to be present in the core of Alzheimer’s disease filaments, packing inside the in-register cross β-sheet structure through a heterotypic, non-staggered interface with the opposing residues, ^373^THKLTF^378^.^6^ Recently, cryo-EM structures of fibrils of VQIINK (PHF6∗) allowed Seidler et al. to hypothesize that the core of the fibrils (PHF6) might be not the primary driver of aggregation, but might serve as a solvent-excluded scaffold that can cluster PHF6∗ together in the fuzzy coat, which poises the solvent-exposed VQIINK steric zippers for seeding.^9^

Tau is considered an intrinsically disordered protein (IDP), containing a high proportion of polar and charged amino acids, which results in the absence of a well-defined three-dimensional structure.^12^ Kadavath et al. showed, through NMR analysis, that although tau is highly flexible in solution and can form β-sheet structure only in amyloid fibrils, it can locally fold into a stable structure upon binding to MTs. This conformation is characterized by a hairpin shape, involving the hexapeptides PHF6 and PHF6∗ located at the beginning of R3 and R2, respectively.^3^ By definition, a β hairpin refers explicitly to a reverse turn connecting adjacent strands in an antiparallel β sheet.^13^ When tau plays its physiological role, the two amyloid “hot spots” adopt a favorable conformation, allowing the protein to maintain its soluble monomeric form and thus its MT interaction. Chen et al. supported this hypothesis by showing that tau adopts a β-hairpin-like compact structure that shields the ^306^VQIVYK^311^ motif and mitigates aggregation. They also claimed that disease-associated mutations close to this motif may contribute to tau molecular rearrangement from an inert to an early seed-competent form.^14^ By using cross-linking reaction and mass spectrometry, Mirbaha et al. demonstrated that tau monomer could exist as inert and seed-competent stable monomer states, and it is only when tau assumes an open configuration, thus making the two ^306^VQIVYK^311^ and ^275^VQIINK^280^ sequences more accessible, that tau monomer produces larger seed-competent assemblies.^10^ Fernández-Ramírez et al. proposed that monomeric tau forms a compact β-hairpin that masks the aggregation-prone ^275^VQIINK^280^ and ^306^VQIVYK^311^ hexapeptides, resulting in an aggregation-defective conformer.^11^ By combining all of these observations, we envisaged that PHF6 is the key amyloid motif, which triggers the aggregation propensity of tau by exposing its hydrophobic character in an extended conformation manner. Contrary to the native state, where hydrophobic residues of PHF6 are protected by a β-hairpin-like compact structure, in which PHF6 can directly interact with PHF6∗, the misfolded protein exposes hydrophobic residues which can form hydrophobic interactions, driving the self-assembly. The removal of PHF6 from PHF6∗ might explain why R2 is not involved in the formation of the NFT core. Moreover, 3R-tau has a higher propensity to form dimers and HMW oligomers than 4R-tau isoforms, and that the absence of the second repeat promotes the aggregation.^15^

Our understanding of tau aggregation has come mostly from *in vitro* studies using either tau models, in which exogenous additives are required to initiate tau aggregation, or tau fragments, which can spontaneously aggregate.^6^ T40 (2N4R) full-length tau has been extensively used to study the aggregation process.^16^ The disadvantage of this model is the requirement of heparin, RNA, or fatty acids to induce self-aggregation, leading to the formation of filaments that differ from the core of AD filaments. Because of their main involvement in the NFTs core formation, tau fragments that only comprise the MTBD (K18/K19) represented an easy and common tau model for studying the entire aggregation process. On the other hand, this approach suffers from the same problem of structural dissimilarities of the filaments produced, especially regarding the packing of PHF6 hexapeptide.^5^ To date, the dGAE, fragment (residues 297–391), corresponding to one of the species isolated from the proteolytically stable AD PHF core preparations,^17^ is considered a consistent and reliable model for understanding pathological tau self-assembly and for screening inhibitors, thanks to its ability to self-assemble spontaneously without cofactors. However, although the tau model evolved and arrived to reproduce the correct packing of the NFTs core, the disease-specific conformations and the mechanism of the misfolding transition remain unknown. As a result, other models able to mimic the conformational change of tau are needed to improve the knowledge of the local and global structural changes in tau, which are responsible for its capacity to form a seed-competent conformation.

Chemical model systems that are simpler and easier accessible than full-length amyloid proteins but inspired by their sequences can be designed to behave in controlled fashions to form well-defined conformations. These model systems can afford structures at atomic resolution, providing insights into the structures and modes of folding of amyloid proteins. Such model systems have been, for example, largely employed by Nowick et al. to elucidate the structures of amyloid oligomers. These oligomers are formed by macrocyclic β-hairpin mimics derived from Aβ, α-synuclein, and β2-microglobulin.^18^^,^^19^ The macrocyclic β-hairpin peptides consist of two peptide β-strands from the amyloidogenic peptide or protein that are constrained by two turn units of ^δ^Orn.

Peptidomimetic foldamers are synthetic molecules that mimic the structure of proteins. They have been developed primarily to mimic the 3D structures and functions of biopolymers, particularly nucleic acids and proteins, through a biomimetic approach. In fact, they are characterized by unique conformations that are inspired by the structural features of their bioactive peptide counterparts.^20^^,^^21^^,^^22^^,^^23^^,^^24^^,^^25^^,^^26^^,^^27^ Several examples of peptidomimetic foldamers can be found in the literature in a wide range of applications, such as catalysis, medicinal chemistry, and materials science.^28^^,^^29^^,^^30^ To our knowledge, no examples of foldamers application in chemical biology, as chemical model systems for the understanding of amyloid aggregation process, are present in literature. This article presents the application of modular isoxazoline-β^2^-amino acid (Isox-β^2^-AA) based peptidomimetic foldamers as chemical model system for studying the tau misfolding mechanism. The two “hot spot” sequences of tau (PHF6 and PHF6∗) have been forced to adopt either a totally extended conformation or a β-hairpin, according to the *(S)* or *(R)* stereochemistry of the scaffold, respectively. It is here demonstrated that an extended conformation, exposing the hydrophobic residues of both sequences and driving away PHF6∗ from PHF6, can trigger the aggregation of tau and behaves as a seed-competent model system. Furthermore, the insertion of the *(R)*-(Isox-β^2^-AA) into peptide sequences based on the Hsp90 chaperone protein demonstrated to be a valuable way to rationally design stable β-hairpin compounds, acting as potent modulator of tau aggregation.

## Results

### Design

Recently, some of us showed that the non-coded Isox-β^2^-AA can promote an α-turn or extended conformations when inserted into tripeptide sequences, depending on the absolute *R*- or *S*- configuration at isooxazoline C-5.^31^ Based on these results, we decided to employ this Isox-β^2^-AA, to assess its potential as a modulable inducer of secondary structure into peptide sequences, able to stabilize either an extended or a β-hairpin conformation, accordingly to the C-5 stereocenter. Although β-amino acids have been extensively studied and employed to stabilize specific conformation,^22^^,^^23^^,^^24^^,^^25^^,^^26^ there are still no examples of a single β^2^-AA demonstrating the ability to induce β-turn folding in long peptide sequences.

As previously mentioned, the two hexapeptide sequences of tau, known as PHF6∗ (^275^VQIINK^280^, 2nd repeat (R2)) and PHF6 (^306^VQIVYK^311^, 3rd repeat (R3)) either are involved in a hairpin-like conformation, when tau physiologically interacts with MTs,^3^ or they are the “hot spot” sequences triggering tau oligomerization and aggregation in pathological conditions.^7^^,^^9^^,^^10^^,^^32^^,^^33^ The inert seed competent tau monomer structural model reported by Mirbaha et al.,^10^ masking of VQIINK and VQIVYK sequences in compact ‘hairpin’ structures (Figure 1B), is similar to the structure of microtubule-bound tau previously determined by Kadavath et al.^3^ Despite different studies supporting the hypothesis that the extended conformation of these sequences promotes the pathological aggregation of tau,^10^^,^^34^ a mechanistic explanation is still lacking. Therefore, with the objective to build two original chemical model systems mimicking the seed-incompetent and seed-competent monomer conformation of tau, as reported in Figure 1, Isox-β^2,2^-AA was used as a tool to build extended or β-hairpin conformations when inserted in peptide sequences. We linked to the *R-* and *S-* scaffold the two hexapeptide motifs PHF6∗ and PHF6 at *N-* and *C*-termini, respectively, to better understand if a conformational change from a β-hairpin (compound **2**) toward an extended structure (compound **1**) might trigger the wild type- (wt-) tau aggregation (Figures 1A and 1B, 1D and 1E).

Finally, to verify the ability of Isox-β^2^-AA to induce more stable β-turn like folding in a long peptide sequence and to behave as synthetic chaperone modulator of tau aggregation in β-hairpin conformation, as previously described by some of us,^35^^,^^36^ the S4 and S7 peptide sequences of the *N*-terminal portion core of Hsp90^37^ were attached to the *R*-configured Isox-β^2^-AA (Figures 1C and 1F).

### Synthesis

**Scheme 1:**
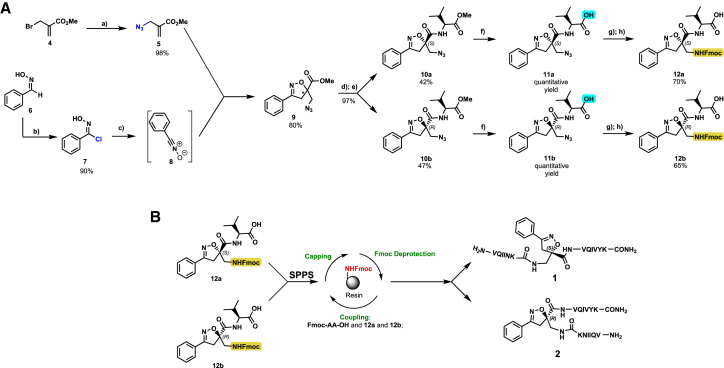
Synthetic approach of compounds 1 and 2 (A) Liquid phase synthesis: a) NaN_3_ (2 eq), acetone/H_2_O (3:1), 20 min, r.t.; b) NCS (1.1 eq), DMF, 40°C, 3h; c) **5** (1 eq), **7** (1.1 eq), TEA (2 eq), r.t., 16h; d) LiOH 0.1M in H_2_O (2 eq), THF (1M), r.t., 30 min; e) HCl·H-Val-OMe (1.5 eq), T3P (3 eq), DIPEA (3 eq), DCM, r.t., 16h; f) LiOH 0.1M in H_2_O (1.2 eq), THF (1M), r.t., 30 min; g) PMe_3_ (1.1 eq), H_2_O (7 eq), THF (0.1 M); h) Fmoc-OSu (1.1 eq), DIPEA (2 eq), DCM (0.1 M), 0°C to r.t., 4h; (B) Liberty Blue synthesis. *Fmoc cleavage*: 20% piperidine in DMF (65s, 90°C, mw); *Coupling:* Fmoc-AA-OH (5 eq), DIC (5 eq), OXYMA (5 eq) (110s, 90°C, 40W, mw); *Coupling of the scaffold:* Fmoc-Isox-β^2^-AA-OH (2 eq), DIC (2 eq), OXYMA (2 eq), DMF/DCM, r.t., 16h; *Cleavage from resin:* trifluoracetic acid/water/thioanisol/triisopropyl silane 95/2.5/1.25/1.25 for 2 h; *Purification:* H_2_O/ACN +0.1% TFA; 20 to 70 gradient ACN in 20 min.

Isoxazoline intermediate **9** was synthesized through 1,3-dipolar cycloaddition between the azido substituted dipolarophile **5** and nitrile oxide **8** (Scheme 1A). The commercially available bromo-methacrylate **4** was first subjected to a nucleophilic substitution using NaN_3_ [acetone/H_2_O (3:1), 20 min, 25°C] to obtain the azido methacrylate derivative **5** in nearly quantitative yield. To obtain nitrile oxide derivative **7**, first chlorooxime **7** (90%) [NCS (1.1 eq), DMF, 40°C, 3 h] was prepared from commercially available benzaldehyde oxime **6**. The treatment of **7** with triethylamine (TEA; 2 eq) leads to the *in situ* generation of the nitrile oxide derivative **7**. Its reaction with **4** forms the 5-disubstituted isooxazoline compound **9** in good yield (up to 80%), as a racemic mixture.^31^ Regarding the regioselectivity, the oxygen atom of the nitrile oxide preferentially reacts with the most substituted carbon atom of the dipolarophile, generating only the product substituted at the 5-position. The regioisomer at the 4-position was not observed, nor in traces. The methyl ester group of **9** was hydrolyzed to produce the corresponding carboxylic acid in quantitative yield through a standard saponification reaction [LiOH (2 eq), THF/H_2_O, 25°C, 30 min]. To resolve the racemic mixture, accordingly with our designed sequences, we performed a first coupling with H-Val-OMe hydrochloride salt (1.5 eq) in the presence of propylphosphonic anhydride (T3P; 3 eq) and DIPEA (4 eq) at pH 8–9. This reaction allowed the formation of a mixture of **10a** and **10b** with an overall yield of 97%. Subsequently, the two diastereoisomers **10a** and **10b** were separated by flash chromatography (DMC/Et_2_O, 98/2) in 42% and 47% yield, respectively. As reported above, the methyl ester of **10a** and **10b** was hydrolyzed, affording carboxylic acid derivatives **11a** and **11b** in quantitative yields without any trace of Val-racemization, as proved by NMR analysis. Crystals were prepared by slow evaporation of a solution containing **11b** dissolved in Et_2_O/*n*-Hexane to define the correct absolute stereochemistry. The X-ray analysis of the obtained **11b** crystals allowed the determination of the absolute *R*-stereochemistry of C-5 of the isooxazoline ring. Indirectly, the *S*-stereochemistry was assigned to diastereoisomer **11a**.

The azido moiety of **11** was then reduced through a Staudinger reaction to afford the amine intermediate of the Isox-β^2,2^-AA **[**PMe_3_ (1.1 eq), H_2_O (7 eq), THF (0.1 M), 16 h, rt]. This crude product was then removed from the solvent and directly used for the next synthetic step. By treating compounds **11** with Fmoc-succinimide (Fmoc-OSu) in DCM and DIPEA (2 eq) (4 h, from 0°C to rt), protected compounds **12a** and **12b** were obtained in good yields (65% and 70%, respectively over three synthetic steps), as compatible intermediates for an SPPS synthetic approach.

The synthesis of the final compounds **1** and **2** was performed using the Liberty BlueTM (CEM) Automated Microwave Peptide Synthesizer under classical conditions (Scheme 1B): the coupling reaction was accomplished at 25°C for 120 s, followed by 480 s at 50°C and 35 W. We used Rink-amide resin (0.1 mmol scale, resin loading = 0.55 mmol/g) and commercially available Fmoc-AA-OH (5 eq, 0.2M), DIC (5 eq, 0.2M), OXYMA (5 eq, 0.2M), previously solubilized in DMF. The Fmoc group was cleaved with a standard deprotection protocol (20% piperidine in DMF) at 75°C, 155 W for 15 s followed by 60 s at 90°C, 50 W. The coupling of **12a** and **12b** was manually performed using the pre-activation step in the presence of 2 equivalents of **12a** or **12b**, DIC (2 eq), and OXYMA (2 eq) in a DCM/DMF (1/3) mixture during 16 h at room temperature. Afterward, the remaining amino acids were coupled under the analog conditions indicated above. Finally, after resin cleavage using an acidic cocktail (TFA/H_2_O/thioanisol/triisopropyl silane; 95/2.5/1.25/1.25). The final peptidomimetic **1** and **2** were purified by semipreparative HPLC using H_2_O/ACN +0.1% TFA with a 20 to 70 gradient in 20 min.

**Scheme 2:**
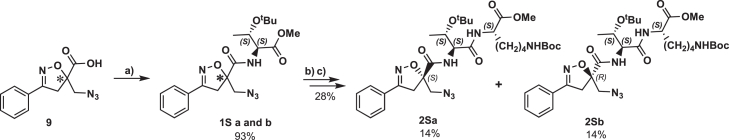
Synthesis and separation of 2Sa and 2Sb (a) **9** (1 eq), HCl·H-Thr(O^t^Bu)-OMe (1.5 eq), T3P (3 eq), DIPEA (3 eq), DCM, r.t., 16h; b) LiOH 0.1M in H_2_O (1.2 eq), THF (1M), r.t., 30 min; c) H-Lys(Boc)-OMe (1.5 eq), T3P (3 eq), DIPEA (3 eq), DCM, r.t., 16h.

**Scheme 3:**
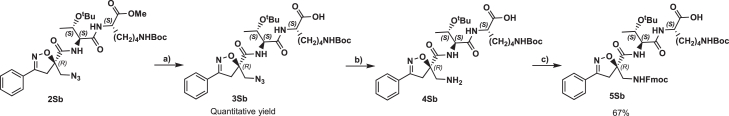
Synthesis of the Fmoc-protected *R*-Isox-β^2^- Thr(O^t^Bu)Lys(Boc)-OH (5Sb) a) LiOH 0.1M in H_2_O (1.5 eq), THF (1M), r.t., 30 min; b) PMe_3_ (7 eq), H_2_O (49 eq), THF (0.1 M); c) Fmoc-OSu (1.1 eq), DCM (0.1 M), 0°C - > r.t., 4h.

Compound **3** was synthesized using the same synthetic approach, with the exception of the diastereomeric mixture being separated by flash chromatography (Hex/EtOAc, 3/2) after two consecutive couplings (Thr and Lys) on intermediate **9** instead of one (for details, see method details, Scheme 2 and 3).

### Conformational study and self-aggregation assessment with and without heparin of compounds (1) and (2)

Compounds **1** and **2**, both bearing the two nucleation sites of tau, were predicted to be self-aggregative. So, for the assessment of their respective conformation and self-aggregation behavior, according to the to the *(S)* or *(R)* stereochemistry of the scaffold, they were preventively subjected to the HFIP treatment before being employed, to ensure as much as possible their monomerization.

**Figure 2:**
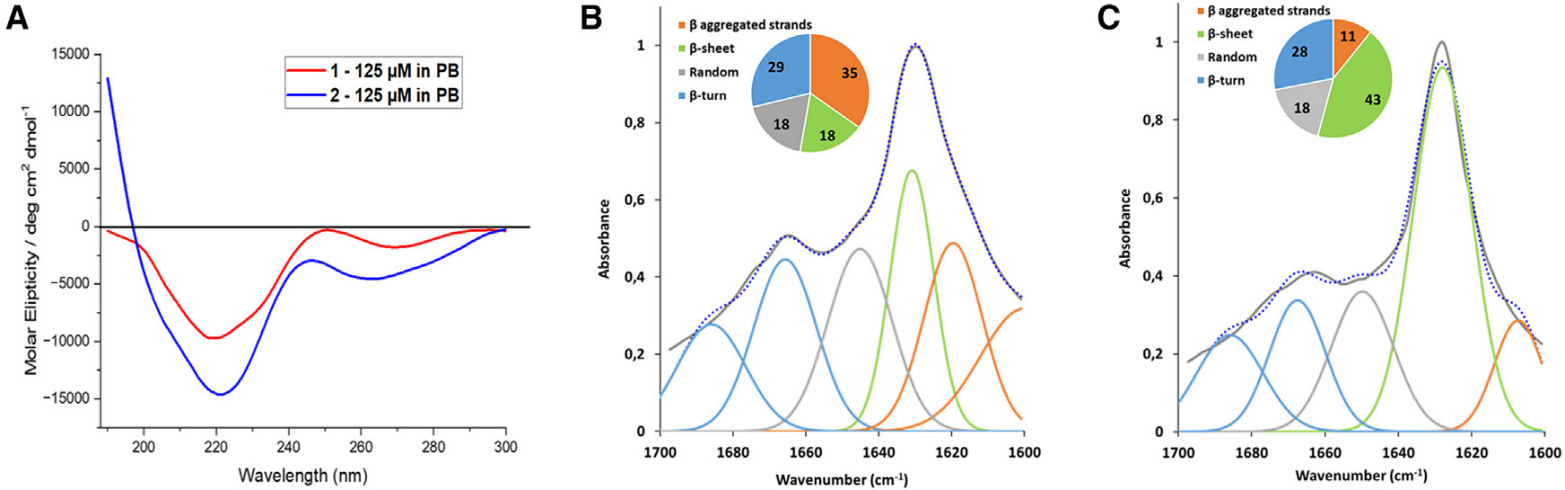
Conformational analysis of compounds **1** and **2** (A) CD spectra of compounds **1** and **2**; B-C) IR-ATR amide I deconvolution of **1** (B) (error squared = 0.029, standard deviation 8.83) and **2** (C) (error squared = 0.042, standard deviation 7.16) with the schematic representation of the secondary structure motif. Both compounds were pre-treated with HFIP and immediately employed at the concentration of 125 μM in 10 mM PB, pH 7.2.

Circular dichroism (CD) analyses were conducted at 125 μM concentration at room temperature in 10 mM phosphate buffer (PB) at pH 7.2 (Figures 2A, S1, and S2, supplemental information). Both compounds showed a negative broad band at around 220 nm and a positive band at 198 nm, characteristic of β-sheet structures. At the same concentration, the CD spectrum of **1** is less intense than compound **2**, characteristic of self-assembly peptides.^38^

Both compounds were also subjected to IR analysis in 10 mM PB (pH 7.2) at the same concentration of 125 μM. As shown in Figures 2B and 2C, the amide I deconvolution of **1** showed a predominant content of aggregated strands (35%), β-sheet structures (18%) and β-turn (29%) (Figure 2B). On the other hand, compound **2** displayed a minor content of aggregated strands (11%), a higher content of β-sheet (43%), and similar contents of β-turn (28%) (Figure 2C). Taken together, the results agree with the CD spectra of **1** and **2**. Compound **1** with the *S*-Isox-β^2^-AA showed a greater tendency to self-assembly in β-sheet rich insoluble structures than **2**, containing *R*-Isox-β^2^-AA, which seems to be more stable as a β-hairpin conformation and less prone to stack into supramolecular structures.

**Figure 3:**
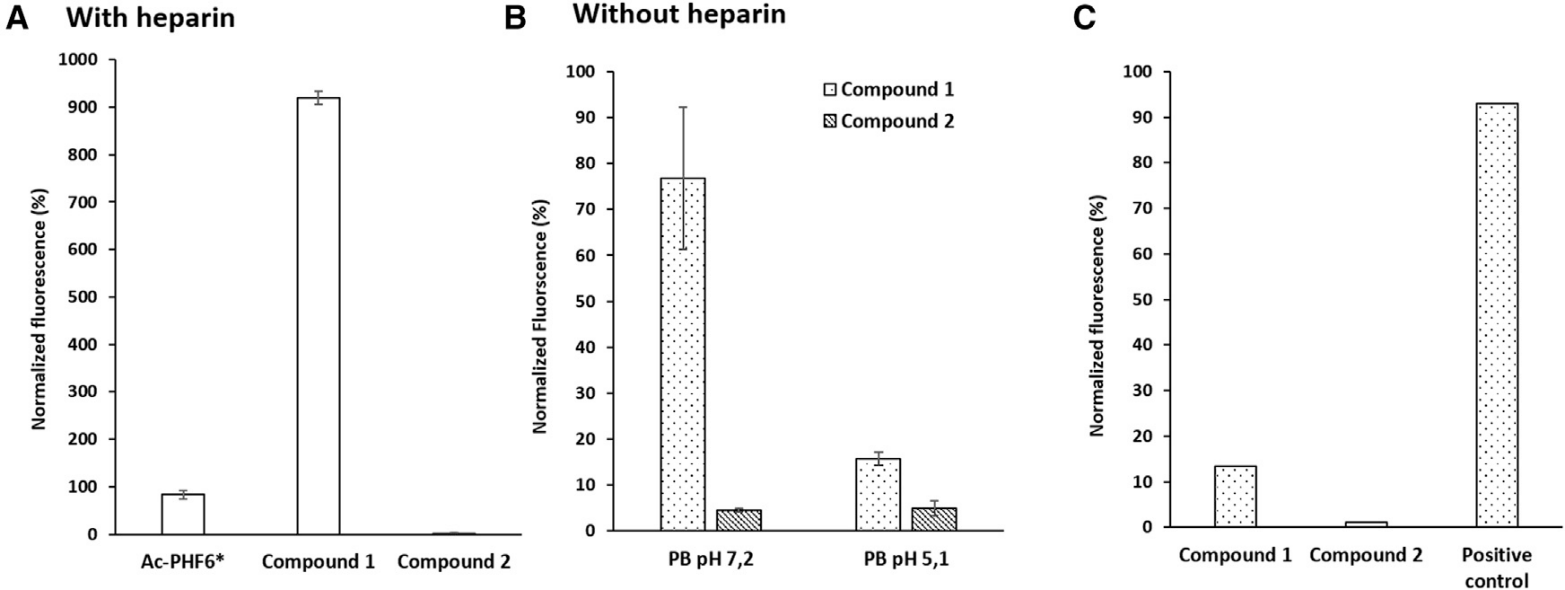
ThT fluorescence spectroscopy assay (A) Self-assembly of compounds **1** and **2** (25 μM) assessed in comparison to the model peptide Ac-PHF6∗ (25 μM) in MOPS 20 mM buffer at pH 7.4 in the presence of 1 μM of heparin, as reported in the literature.^40^ Fluorescence data have been normalized by putting the max of fluorescence of Ac-PHF6∗ as 100%. Data are represented as mean ± SEM. (B) Self-assembly of compounds **1** and **2** (25 μM) in the absence of heparin in phosphate buffer at two different pH, 7.2 and 5.1. Data are represented as mean ± SEM. (C) Self-assembly without heparin of compounds **1** and **2** (1 μM) without heparin under the conditions for tau aggregation assessment (25 mM PB buffer at pH 6.6). Positive control: tau (10 μM) in the presence of heparin (0.1 μM). Data are represented as mean ± SEM.

The hexapeptide PHF6∗ is often used as a model for understanding the aggregation of the full-length tau protein.^9^^,^^39^^,^^40^ It is well known that PHF6∗ at 25 μm in the presence of heparin shows an aggregation kinetic profile very similar to PHF6, reaching a plateau within 30 min. So, we first investigated the self-aggregation propensity and β-sheet formation of compounds **1** and **2** by employing the same protocol as the one described in the literature for Ac-PHF6∗-NH_2_,^40^ using a Thioflavin-T (ThT) fluorescence spectroscopy assay (Figure 3A). The assay was performed in the presence of heparin (1 μM) in 20 mM MOPS buffer at pH 7.4 (Figure 3A). After 60 min, compound **1** showed an extremely high ThT fluorescent signal, nearly nine times higher than the model Ac-PHF6∗-NH_2_, confirming a strong self-aggregative behavior. On the other hand, the ThT signal in the presence of compound **2** did not increase, suggesting no self-aggregation (Figure 3A). All the results are in agreement with the previous observations by CD and IR. However, to further investigate if this self-aggregation persists even without heparin (as observed in CD and IR), both compounds were assessed by ThT fluorescence spectroscopy in the same conditions of the previous physical-chemical analyses (PB 10 mM pH 7.2). Compound **1** demonstrated to maintain its aggregation propensity in phosphate buffer, while compound **2** did not show any aggregation (Figure 3B). Interestingly, lowering the pH from 7.2 to 5.1 led to a considerable decrease in the ThT signal of compound **1** (Figure 3B), demonstrating less aggregation propensity at pH 5.1, also confirmed by the CD analysis (Figure S3, supplemental information). Finally, both compounds were also tested at 1 μM concentration under the conditions for tau aggregation assessment, and none of them showed self-aggregation in the absence of heparin. All together, these results allowed to highlight a different self-aggregation behavior of compounds **1** and **2** according to the *(S)* or *(R)* stereochemistry of the scaffold, even if they are composed by the same hexapeptide sequences, and to find the correct conditions to ensure a stable monomerization.

To confirm that **1** and **2** were able to adopt a stable extended conformation and a β-hairpin-like structure, respectively, 1D and 2D homo and heteronuclear NMR conformational studies were performed in 10 mM PB, at pH 5.1 (final peptide concentration of 3.6 mM). This pH value was chosen because in this condition both compounds have a comparable high monomer stability (Figures 3B and S3, supplemental information).

^1^H and ^13^C resonances were completely assigned using 1D 1H WATERGATE, 2D ^1^H–^1^H COSY (20 ms mixing time), 2D ^1^H–^1^H DIPSI (20 ms mixing time), 2D ^1^H–^1^H ROESY (20 ms mixing time) and 2D ^1^H-^13^C HSQC, 2D ^1^H-^13^C HMBC. The resonances were assigned using spectrum acquired at 283K, other supplementary spectra were recorded respectively at 298K, 318K, 328K for **1** and 293K, 303K and 313K for **2**, to evaluate the presence of intramolecular hydrogen bonds through temperature coefficient calculation. ^1^H and ^13^C chemical shifts were calibrated on the methyl moiety of aliphatic amino acids. The vicinal coupling constants (^*3*^*J*) were extracted from 1D ^1^H and 2D ^1^H–^1^H COSY spectrum at 283K.

^1^H NMR spectrum of **1** showed a good dispersion of the NH chemical shifts indicating a single conformation. A complete attribution of all the corresponding chemical shifts is provided in Table S1 (see supplemental information).

**Figure 4:**
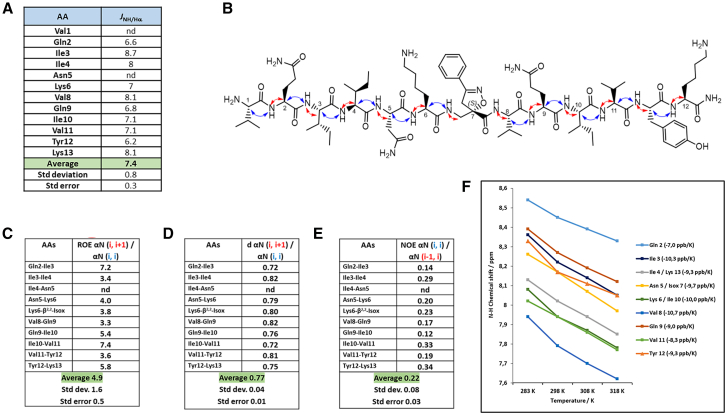
NMR conformational analysis of compound 1 in 20 mM phosphate buffer at pH 5.1 (A) Values of ^*3*^*J*_*NH-Hα*_ coupling constants; (B) Representation of the CH/NH (*i*, *i*) ROEs (red) and the CH/NH (*i*, *i+1*) ROEs (blue); (C) ROE intensity ratio of αN (*i*, *i+1*)/αN (*i*, *i*); (D) H_α_-NH distances; (E) ROE intensity ratio of αN (*i*, *i*)/αN (*i-1*, *i*); (F) Temperature coefficients of amide protons between 283 and 318 K. Data are represented as mean ± SEM.

The vicinal ^*3*^*J*_NH-Hα_ coupling constant showed an average value of 7.4 ± 0.3 Hz, discarding the possibility of a folded structure. However, this value appears to be closer to the coupling constant found in β-sheets (8.9 Hz for antiparallel and 9.7 for parallel) than in α-helices (3.9–4.2 Hz) (Figure 4A). Both peptide arm sequences present a complete set of strong CH/NH (*i*, *i+1*) ROEs, suggesting an extended conformation for each peptide arm (Figure 4B, blue and red curves). Additionally, this hypothesis is supported by the lack of intra-strand NH/NH (*i*, *i+1*) ROEs and the absence of inter-strand ROEs. Neither cross-strand ROEs interaction between the two peptide arms nor any spatial proximity between the -CH_2_ protons of the Isox-β^2^-AA^7^ and the NH/CH_α_ protons of the Val^8^ were observed.

As demonstrated by Dobson et al.,^41^ the intensity ROEs αN (*i*, *i+1*)/ROEs NN (*i*, *i+1*) ratio can indicate the propensity of an amino acid to adopt a specific secondary structure. A ratio value close to 1 suggests increased α-propensity, values around 1.4 indicate the tendency to form random coil regions and values of approximately 2.7 reveal a higher-than-average β-propensity. For compound **1**, the lack of ROEs NN (*i*, *i+1*) leads to an elevated ratio, which is a characteristic of residues with higher-than-average β-propensity.

Additionally, ROEs αN (*i*, *i+1*)/ROEs αN (*i*, *i*) intensity ratio about 2.3 is predicted for the population-weighted random coil model, whereas values exceeding 4 are expected for β-strands.^42^ Upon calculation of this ratio for **1**, the resulting average value of 4.9 ± 0.5 suggests the presence of a general extended conformation (Figure 4C).

Finally, ROEs αN (*i*, *i*)/ROEs αN (*i-1*, *i*) intensity ratio is known to be influenced by the ψ angle of residue *i − 1*, with values ranging from around 6 for α-helices to roughly 0.25 for β-sheets (ratio <1).^43^ A ratio less than 1 is indicative of β-sheets, whereas a ratio greater than 1 suggests α-helices.^44^ An average value of 0.22 ± 0.03 is in line with an extended conformation for compound **1** (Figure 4E).

Furthermore, the ratio of sequential to intra-residue H_α_-NH distance varies according to the secondary structure: 1.25 for α-helices and 0.73 for β-strands (where d αN (*i*, *i+1*) α-helix = 3.5 Å and d αN (*i*, *i*) α-helix = 2.8 Å; d αN (*i*, *i+1*) β-sheet = 2.2 Å and d αN (*i*, *i*) β-sheet = 3.0 Å).^45^ In our case, with an average value of 0.77 ± 0.01, the data fits once more with an extended conformation adopted by **1** (Figure 4D).

Any of the amide protons were involved in H-bond as demonstrated by all temperature coefficients (Δδ_NH_/ΔT) below −4.5 ppb K^−1^, and an average value of −9.13 ppb K^−1^ (Figure 4F).

**Figure 5:**
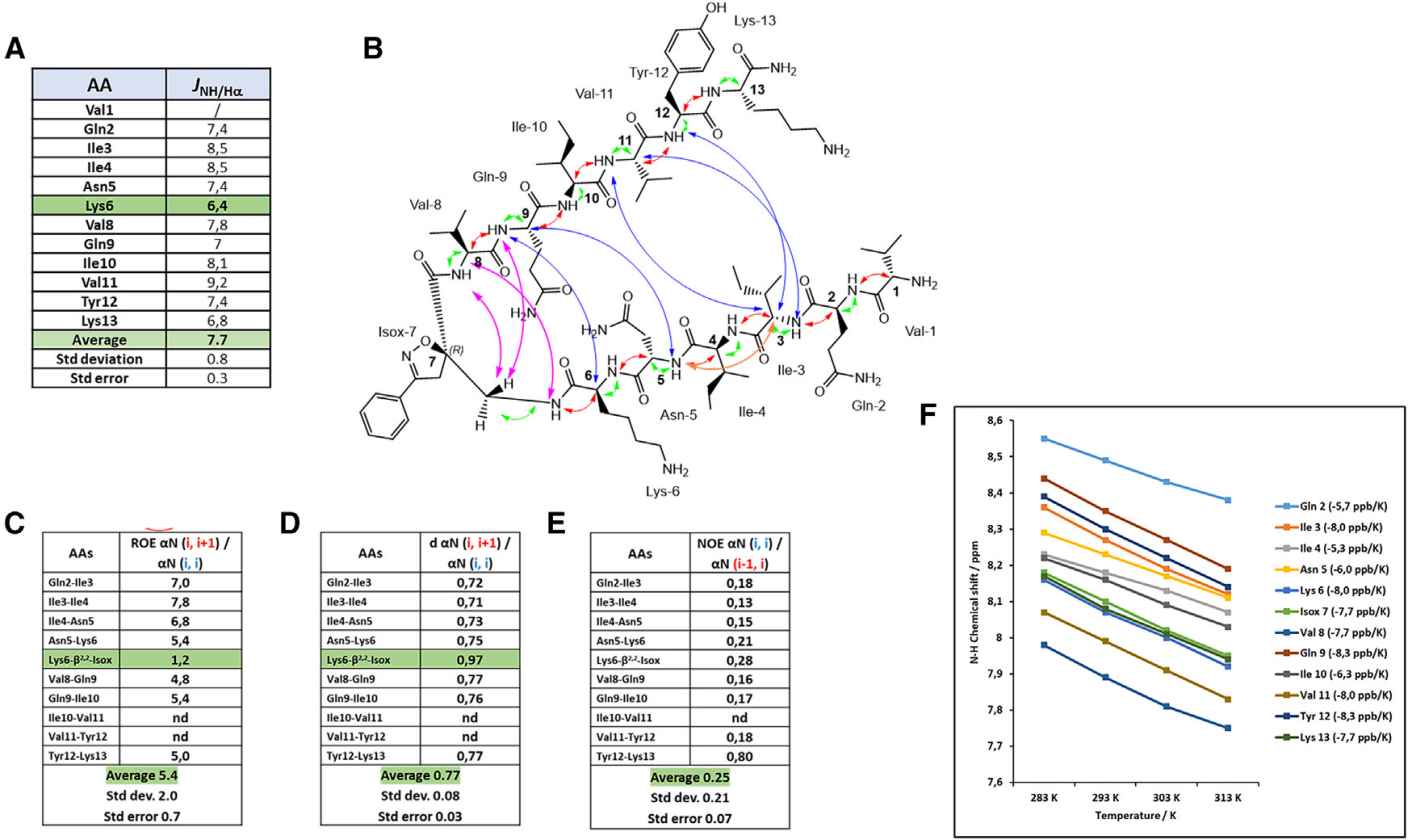
NMR conformational analysis of compound 2 in 20 mM phosphate buffer at pH 5.1 (A) Values of ^*3*^*J*_*NH-Hα*_ coupling constants; (B) Representation of the CH/NH (*i*, *i*) ROEs (green), the CH/NH (*i*, *i+1*) ROEs (red), the intra-strand NH/NH (*i*, *i+1*) ROEs (orange), the inter-strand CH-NH ROEs in the turn region (purple), and the long-range inter-strands ROEs (blue); (C) ROE intensity ratio of αN (*i*, *i+1*)/αN (*i*, *i*); (D) H_α_-NH distances; (E) ROE intensity ratio of αN (*i*, *i*)/αN (*i-1*, *i*); (F) Temperature coefficients of amide protons between 283 and 318 K. Data are represented as mean ± SEM.

Even for compound **2**, the ^1^H NMR spectrum showed a good dispersion of the NH chemical shifts indicating the presence of a single conformation. A complete attribution of all the corresponding chemical shifts is provided in Table S2 (see supplemental information). The average vicinal ^*3*^*J*_NH-Hα_ coupling resulted to be 7.7 ± 0.3 Hz, suggesting an extended conformation of both peptide arms. Remarkably, although the *J* coupling constants of **2** are similar or slightly larger than **1**, an interesting difference is observed in the ^*3*^*J*_NH-Hα_ coupling constant of Lys^6^ in **2**. Lys^6^ is located just before the Isox-β^2^-AA, and exhibits a reduction from 7 Hz in **1** to 6.4 Hz in **2**. According to the Karplus formula, this reduction is consistent with a smaller ϕ dihedral angle, and, therefore, provides evidence for the formation of a local turn (Figure 5A).

The ROESY analysis showed a complete set of strong sequential CH/NH (*i*, *i+1*) ROEs (Figure 5B, red curves) for both peptide arms, suggesting an extended conformation, also demonstrated by the absence of intra-strand NH/NH (*i*, *i+1*) ROEs, leading to an elevated ROEs αN (*i*, *i+1*)/ROEs NN (*i*, *i+1*) ratio, which is a characteristic of residues with higher-than-average β-propensity. The resulting average value of 5.4 ± 0.7 for the ROEs αN (*i*, *i+1*)/ROEs αN (*i*, *i*) intensity ratio also suggests the presence of extended conformations on both peptide arms. Worthy of particular attention, the only ROEs αN (*i*, *i+1*)/ROEs αN (*i*, *i*) ratio lower than 2.3 is the one between Lys^6^ (*i*) and Isox-β^2^-AA^7^ (*i+1*), suggesting a different orientation of the H_α_ (Lys^6^) with respect to the NH of the Isox-β^2^-AA^7^, due to a reduced ϕ dihedral angle value, as seen before for the ^*3*^*J*_NH-Hα_ coupling constant (Figure 5C).

Furthermore, an average value of 0.25 ± 0.07 for ROEs αN (*i*, *i*)/ROEs αN (*i-1*, *i*) intensity ratio is still in line with an extended conformation of the peptide sequences of compound **2** (Figure 5E).

Finally, an average value of 0.77 ± 0.03 for the ratio of sequential to intra-residue H_α_-NH distances well fits with a β-strand conformation adopted by the two peptide arms of compound **2**. Once again, a shift toward 1 (0.97) for Lys^6^ (*i*) and Isox-β^2^-AA^7^ (*i+1*) is observed, compatibly with the presence of a turn in this part of the molecule (Figure 5D).

Interestingly, in contrast to **1**, multiple cross-strands ROEs were observed for compound **2**. The turn conformation is confirmed by the presence of inter-strand CH-NH ROE signals between H_α_ of Val^8^ and NH of Isox-β^2^-AA^7^, H_β_ of Isox-β^2^-AA^7^ and NH of Gln^9^, and H_β_ of Isox-β^2^-AA^7^ and NH of Val^8^ (purple arrows Figure 5B). Additionally, further long-range inter-strand interactions between H_α_ of Lys^6^ and NH of Gln^9^, H_α_ of Gln^9^ and NH of Asn^5^, H_α_ of Val^11^ and NH of Ile^3^, H_α_ Ile^3^ and NH of Val^11^, and H_α_ of Ile^3^ and NH of Tyr^12^ confirm the hairpin conformation alongside the full peptide arms (Figure 5B blue arrows; and Figure S4 in supplemental information). A single long-range intra-strand is found and highlighted with an orange arrow between the NH of Asn^5^ and the H_α_ of Ile^3^, probably indicating a mild twist in this portion (Figure 5B).

As reported in Figure 5F, any of the amide protons are involved in stable H-bond, as demonstrated by all temperature coefficients (Δδ_NH_/ΔT) below −4.5 ppb K^−1^, and an average value of −7.2 ppb K^−1^. It has to be noticed that the two borderline values for Ile^4^ and Val^8^ (5.3 and 5.7 ppb K^−1^, respectively) suggest a partial involvement of the two amide protons in weak H-bonds, and this will be in accordance with an alternating 10- and 14-membered rings, typical of antiparallel β-sheet.

### Tau aggregation in the presence of compound (1) and (2): Evaluation by thioflavin-T fluorescence spectroscopy, transmission electron microscopy, and fluorescence decay after photoactivation

#### Tau aggregation in the absence of heparin is triggered by (1) and not by (2)

To investigate whether the two distinct conformations of compounds **1** and **2** have the potential to induce or prevent Tau aggregation, we exploited ThT fluorescence spectroscopy to obtain real-time information about the supramolecular self-assembly of tau in the presence of **1** and **2**.

We opted to test the compounds at a sub-stoichiometric ratio (1 μM) for two primary reasons. Firstly, we observed that, at higher concentrations, compound **1** exhibits a notable increment in its self-aggregative tendency, thus making the interpretation of the results more challenging (see Figure 3B). Moreover, at this concentration no aggregation of both compounds was observed (Figures 3C, S3, S5E, and S5F, supplemental information). Secondly, by working at the sub-stoichiometric ratio, it can be demonstrated that the presence of a minimal amount of a seed-competent mimic, as compound **1**, could drive the full-length wt-tau toward the formation of fibrils.^5^^,^^46^

Therefore, the two compounds have been incubated with wt-Tau441 (10 μM) at 0.1/1 ratio without heparin in PB buffer (pH 6.6) at 37°C, and the ThT (25 μM) fluorescence emission has been monitored over time. To have a positive control, the aggregation of wt-Tau441 was induced by the addition of a low quantity of heparin (100/1 ratio, 0.1 μM) to provide evidence of the mandatory use of anionic co-factors like heparin to induce the aggregation of Tau *in vitro* and compared to wt-Tau441 without heparin (negative control).

**Figure 6:**
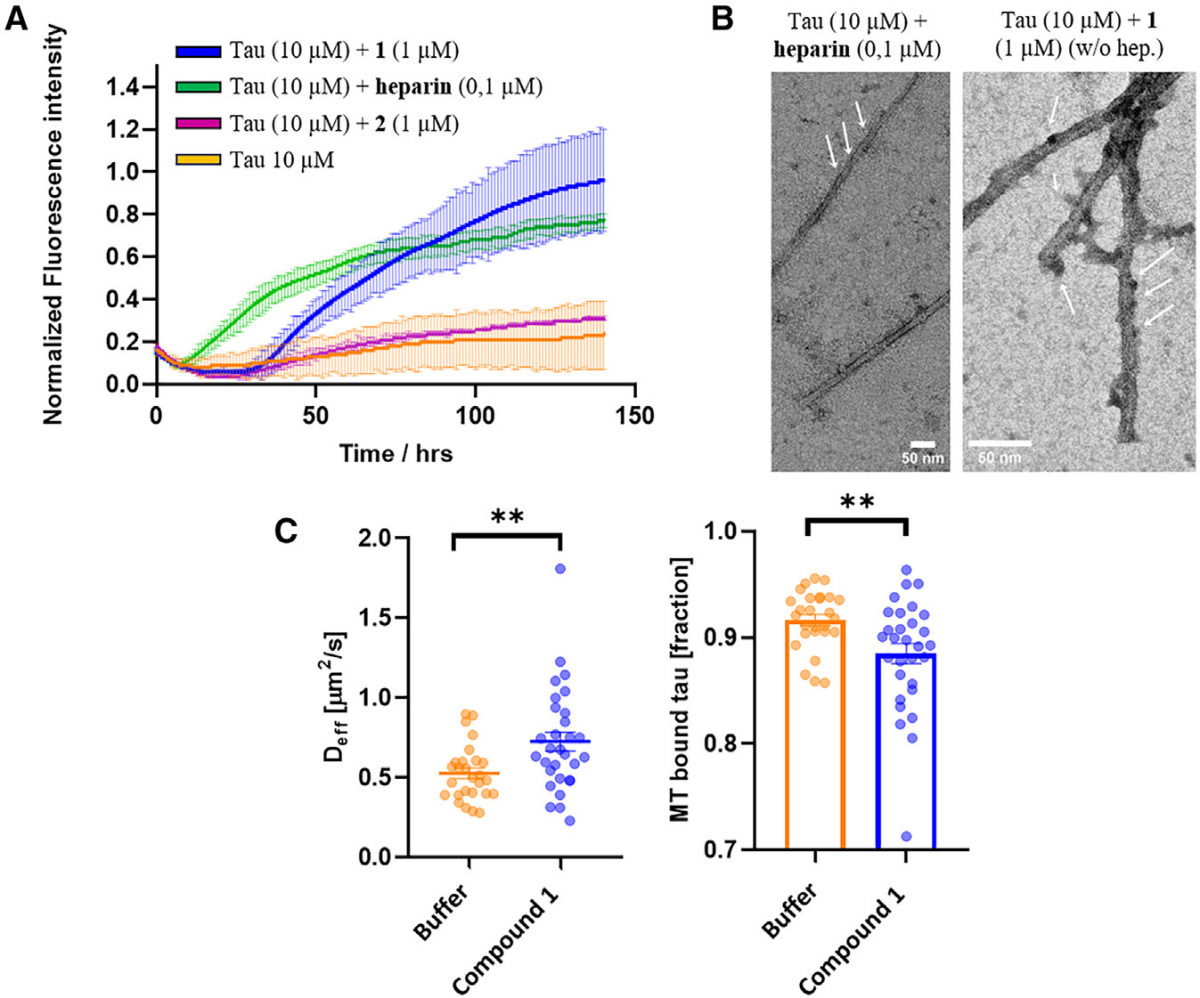
Assessment of tau fibrillation in the presence of compounds 1 and 2 (A) ThT fluorescence spectroscopy of tau (10 μM) in PB buffer (pH 6.6) at 37°C in the presence of 0.1 μM of heparin (green line), 1 μM of compound **1** (blue line), and 1 μM of compound **2** (purple line), or in the absence of any additive (orange line). Fluorescence data were normalized, and the results are represented as the mean of three replicates (*n* = 3) +/− SEM; (B) Representative tau fibrils formed in the presence of heparin (0.1 μM) on the left, and with compound **1** (1 μM) on the right obtained by TEM; arrows show the helical organization of tau alone (left) and the presence of oligomers on the surface of the fibrils (right). The original TEM images are presented in Figure S5. Low-magnification pictures with the corresponding magnification area are in Figure S6; (C) Fluorescence decay after photoactivation (FDAP) analysis of **1** in differentiated model neurons (PC-12 cells) transfected with an aggregation-prone tau construct (PAGFP-Tau441 ΔK280). The decay is fitted with a mathematical model, which allows the analysis of the effective diffusion constant (D_eff_) and the MT-bound fractions of tau in axon-like processes as an indicator of the effect of the compound on tau aggregation.^48^ Presence of **1** increases Tau D_eff_ while decreasing Tau MT bound fraction, indicating that **1** induces increased tau aggregation in the neuronal cells. Experiments were performed with PC12 cells differentiated with NGF, 24 h. Pre-incubation time, DMSO concentration 0.125% (Control). FDAP decay images are provided in Figure S9. Statistical significance was attributed following an unpaired two-tailed t-test, where two stars (∗∗) represents *p* value less than 0.01.

As previously described, the ThT fluorescence value of wt-Tau441 without heparin (orange line) remains at its basal level during 140 h, showing that wt-Tau441 under this condition cannot form amyloid fibrils (Figure 6A), which was confirmed by TEM (Figure S5, supplemental information). On the other hand, in the case of wt-Tau441 in the presence of heparin (0.1 μM), the ThT fluorescence (green line) shows the classical sigmoidal curve characterized by a lag phase (up to 5h), the elongation phase and the plateau reached after approximately 60 h (Figure 6A). This curve follows a molecular model (MRE = 0.00249) in which the mechanism dominating the kinetics of aggregation is the nucleation/elongation (AmyloFit),^47^ characterized by rate constant of elongation and primary nucleation (*k*_*+*_*k*_*n*_) of 4.07x10^29^ conc^-n^ time^−2^. The occurrence of amyloid fibrils with a diameter around 20 nm (Figure 6B, left) with planar and a helical organization of a single protofilament was detected by TEM (Figure 6B, see arrows).

Interestingly, wt-Tau441 in the presence of compound **1** (1 μM) and without heparin (Figure 6A, blue line) shows a ThT fluorescence increase with a classical sigmoidal curve with a lag phase of about 30 h and a growth phase until 140 h, slightly different from the one of control wt-Tau441:heparin. The AmyloFit fitting of the curve suggests a molecular model (MRE = 0.00291) in which the mechanism dominating the kinetic of aggregation is the secondary nucleation (*k*_*+*_*k*_*n*_ = 9.00 × 10^5^ conc^-n^ time^−2^ and *k*_*+*_*k*_*2*_ = 1.82 5 10^16^ conc^-n^ time^−2^). This different kinetic suggests a different aggregation mechanism. Figure 6B (right), shows a change in the morphology of the wt-Tau441 in the presence of compound **1**. These fibrils are thinner than tau fibrils in the presence of heparin, with a diameter of around 12 nm and surrounded by oligomers on their surface, confirming the secondary nucleation mechanism that the ThT assay suggested. On the contrary, wt-Tau441 (10 μM) in the presence of compound **2** (1 μM) (Figure 6A, purple line and TEM images in Figure S5, supplemental information) doesn’t exhibit any aggregation similar to the negative control (tau in the absence of heparin). This result demonstrates that the β-hairpin conformation of **2**, mimicking the inert conformation of tau, cannot trigger the aggregation process. In contrast, compound **1**, through its extended conformation, behaves as a seed-competent template, allowing tau to fold in a seed-competent monomer by similarity of conformation.

Fluorescence decay after photoactivation (FDAP) is a live-cell microscopy technique applied to study tau-microtubule interaction and investigate tau mobility in neuronally differentiated cells.^49^ Parameters such as the effective diffusion constant (D_eff_), the tau microtubule-bound fraction (MT bound tau), the association (K_on_), and dissociation (K_off_) kinetic constants^50^^,^^51^ can be derived by this analysis to evaluate potential changes in the tau-MT interaction in axon-like processes of model neurons. The constructs are N-terminally tagged with photoactivatable GFP (PAGFP) and exogenously expressed in PC-12 cells differentiated into a neuronal phenotype and locally photoactivated using a laser scanning microscope. When used with an aggregation-prone tau construct (Tau441 ΔK280 construct), the assay can be used to determine the effect of exogenously added compounds on modulating tau aggregation.^48^ If a compound is supposed to induce the aggregation of tau in a cell environment, its effective diffusion constant results to be increased due to a pronounce aggregation. Conversely, when the compound prevents the aggregation, the diffusion constant decreases and tau interacts more with MT.

The toxicity of compound **1** alone was tested through an MTT cell viability assay and in different concentrations up to 50 μM. It did not show any statistically significant modification of cellular metabolism on differentiated PC-12 cells (Figure S7, supplemental information). FDAP analysis was performed at a concentration of 25 μM of the pro-aggregative compound **1**. Remarkably, compound **1** significantly increased the effective diffusion constant (D_eff_), indicating a reduced interaction of Tau with the MTs (Figure 6C). Accordingly, the MT-Tau bound fraction calculation showed a significant decrement compared to the control, indicating that compound **1** induces a substantial increase in tau aggregation. We did not observe changes in K_on_ and K_off_ (Figure S8, supplemental information), indicating that compound **1** does not affect the tau/MT kinetics.

Compound **1**, employed as a chemical model system of a seed-competent conformation, allowed us to shed light on the early mechanism behind Tau aggregation. It provided evidence that when the two hexapeptide motifs, PHF6∗ and PHF6, are in an extended conformation, the aggregation of Tau is accelerated, both *in vitro* and in cells.

#### Tau aggregation in the presence of heparin can be controlled by the concentration of the β-hairpin compound **3**

**Figure 7:**
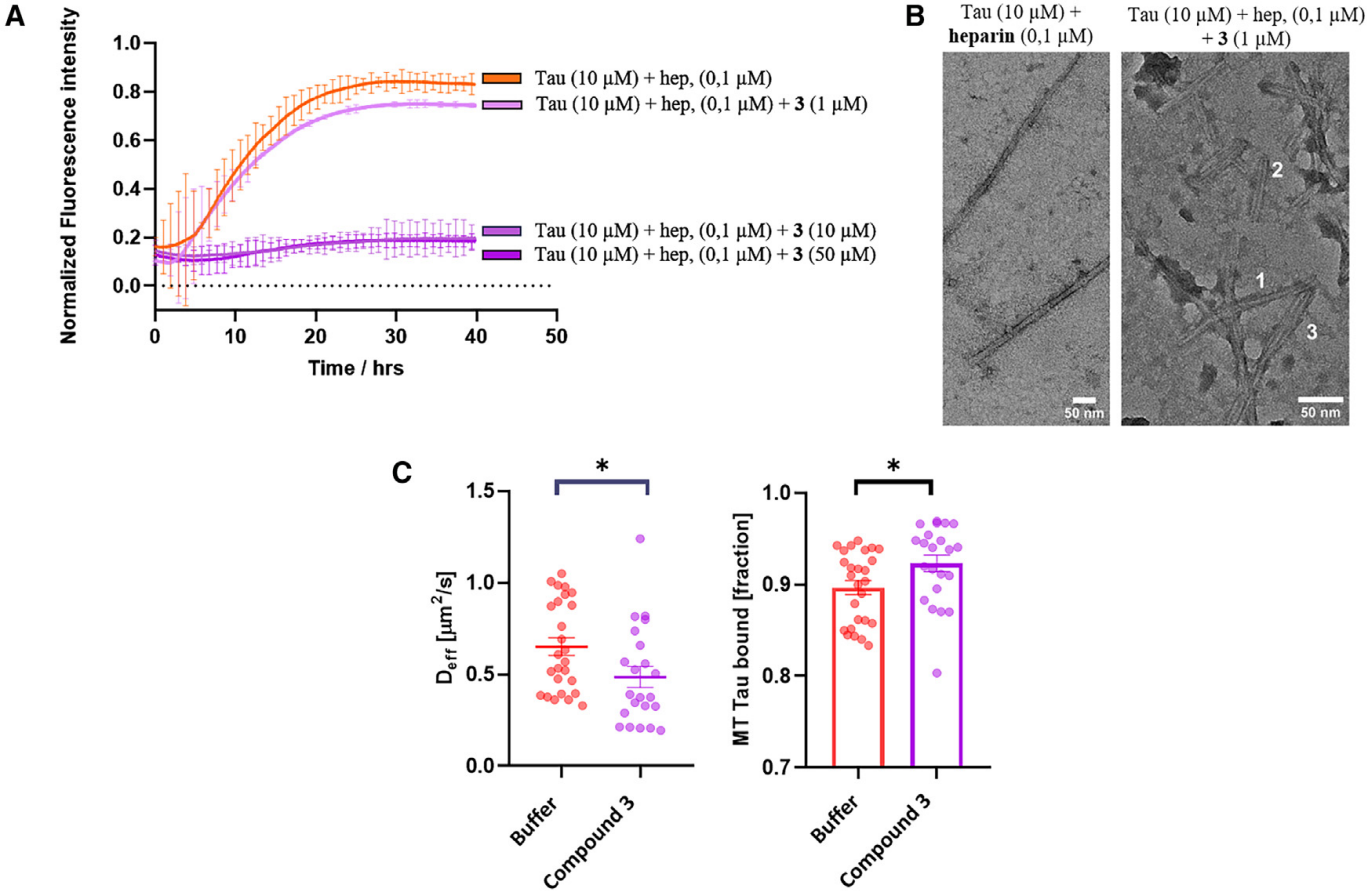
Assessment of tau fibrillation in the presence of compounds 3 (A) ThT fluorescence spectroscopy assessment of compound **3** in the presence of tau (10 μM) in NaPi buffer (pH 6.6) at 37°C and 0.1 μM of heparin at different molar ratios (1:5, 1:1 and 0.1:1, tau/**3**). Fluorescence data were normalized and the results are represented as the mean of three replicates (*n* = 3) +/− SEM; (B) Representative tau fibrils formed in the presence of heparin (0.1 μM) on the left and with compound **3** (1 μM) and heparin (0.1 μM) on the right obtained by TEM. The original image and those for the controls are in Figure S11. The number shows the amount of lateral association. 1 for only one filament, 2 for two filaments and 3 for three filaments; no fibrils were detected when compound **3** was present at 5:1 and 1:1 ratios; (C) FDAP analysis of **3** on PC-12 cells transfected with PAGFP-Tau441 ΔK280. Mathematical modeling shows that compound **3** increases Tau D_eff_ and consequently decreases the tau MT bound fraction at a concentration of the compound of 25 μM, indicating that **3** inhibits tau aggregation in neuronal cells and restores the physiological tau-MT interaction in axon-like processes; Experiments were performed with differentiated model neurons (PC12 cells differentiated with NGF), compound incubation time was 24 h, DMSO concentration 0.125% (Control). FDAP decay images are provided in Figure S13. Statistical significance was attributed following an unpaired two-tailed t-test, where one star (∗) represents *p* value less than 0.05.

As aforementioned, compound **3** was designed according to previous data showing that a β-hairpin mimic, bearing defined sequences (S4 and S7) from Hsp90 (Figure 1C), can inhibit both wt-tau and pro-aggregative ΔK280 tau mutant fibrillation.^35^ To verify the ability of the *(R)*-configured Isox-β^2^-AA to induce a stable β-turn like folding in a long peptide sequence and, thus, to be employable as β-turn inducer for potential inhibitors of amyloid aggregation, the S4 and S7 peptide sequences of N-terminal portion of Hsp90 were attached to the *(R)*-configured Isox-β^2^-AA. Compound **3** forms a stable β-hairpin conformation as demonstrated by CD and IR analyses (Figure S10, supplemental information). The activity of **3** as an inhibitor was assessed according to a classical protocol employing heparin to induce wt-tau aggregation.^52^ The inhibitor activity of **3** resulted dependent on its concentration (Figures 7A and 7B). In the absence of **3**, the ThT-fluorescence curve of wt-tau/heparin (10 μM/0.1 μM) showed a classical sigmoidal shape with a lag phase of 3 h, followed by an elongation phase and a final plateau reached after 14 h (Figure 7A). In the presence of compound **3** at 5/1 and 1/1 ratios, the ThT-fluorescence curve of wt-tau/heparin displayed a complete inhibition of the aggregation, confirmed by the absence of aggregates in TEM (Figure S11, supplemental information). The addition of a sub-stoichiometric quantity of **3** (0.1/1 ratio) shows a ThT kinetic curve similar to the one of tau in the presence of heparin alone, fitting well with a molecular model (MRE = 0.00430) in which the mechanism dominating the kinetic of aggregation is the secondary nucleation, with rate constants (*k*_*+*_*k*_*n*_ = 2.80 × 10^8^ conc^-n^ time^−2^ and *k*_*+*_*k*_*2*_ = 1.59 × 10^19^ conc^-n^ time^−2^) slightly higher than the tau-heparin control (see above). Considering the morphologies, fibrils in the presence of heparin alone are longer and more uniform than the straight filaments in AD-tau seeded filaments (Figure 7B left).^53^ Surprisingly, the morphology of tau in the presence of 0.1 μM of **3** led to the formation of heparin-assembled recombinant tau fibrils that are exclusively short and straight, constituted by one to three filaments associated laterally, more similar to the PHFs and SFs in AD tau.^54^

FDAP experiments to determine the effect of compound **3** in neuronal cells allowed us to demonstrate that β-hairpin **3** at 25 μM significantly decreases the effective diffusion constant of the aggregative prone Tau441 ΔK280 construct (D_eff_, Figure 7C), indicating a raised interaction of tau with the microtubules (Figure 7C) compared to the control. This indicates that compound **3** significantly decreases tau aggregation in neuronal cells thereby restoring a physiological tau-MT interaction. K_on_ and K_off_ parameters were not altered (Figure S12, supplemental information), indicating that **3** reduces tau aggregation but does not affect the association and dissociation of tau to MT.

In conclusion, compound **3**, designed on the Tau-Hsp90 interaction and having a β-hairpin conformation, demonstrated remarkable inhibitory activity against tau aggregation, both *in vitro* and in cells. Furthermore, the data provide evidence that compound **3** is able to restore the physiological tau-MT interaction of aggregation-prone tau in model neurons.

## Discussion

In this research article, we demonstrated that chemical systems, inspired by the conformations of non-pathological and pathological proteins, can be employed to modulate and study the amyloid misfolding and aggregation mechanism of tau. In particular, according to the stereochemistry of a new non-natural β^2^-amino acid, β-hairpin-like and extended conformations could be stabilized in long peptide sequences. Through this folding modulation, we showed that an extended conformation, exposing the hydrophobic residues of both sequences and driving away PHF6∗ from PHF6, can trigger the aggregation of tau in the absence of additives such as heparin, thus behaving like a seed-competent monomer model system. Conversely, a β-hairpin imitates the favorable folding of tau, allowing the protein to maintain its soluble monomeric form and potentially its MT interaction due to the absence of aggregation. Therefore, this β-hairpin conformation cannot induce the aggregation of tau *in vitro*. The extended conformation of our seed-competent monomer mimic can trigger the tau aggregation process, leading to the formation of fibrils, morphologically different from the ones obtained in the presence of heparin. These fibrils are the result of a different kinetic profile than the one observed with heparin. Indeed, a secondary nucleation-dominated mechanism drives wt-tau to form thinner fibrils, more similar to the ones found in the patients’ brains.^7^ CD experiments of tau in the presence of sub-stoichiometric concentration of both compounds might be interesting in the future for an insight characterization of the tau secondary structure in the very early stages of its misfolding. Furthermore, a β-hairpin mimic, characterized by *R-*configuration of the non-natural β^2^-amino acid and based on the chaperone protein Hsp90, demonstrated the further application of this type of peptidomimetic foldamers for the development of inhibitors of tau aggregation. Surprisingly, at a sub-stoichiometric ratio and in the presence of low concentrated heparin, the same compound inspired by the chaperone protein Hsp90, can maintain a secondary nucleation-dominated kinetic, as observed with the seed-competent mimic, showing, however, morphology of the fibrils similar to the straight filaments observed in the brain of patients.^53^ Remarkably, our study shows that peptidomimetic foldamers are capable to modulate tau aggregation in living model neurons thereby influencing the tau-MT interaction in axon-like processes. In conclusion, chemical model systems are not only simpler and more accessible than full-length proteins, but can also be used to study the structures and folding modes of amyloid proteins, as well as to modulate the misfolding and aggregation of an amyloid protein *in vitro* and in cells to obtain a fibril morphology *in vitro* that approximates the pathologically relevant one.

### Limitations of the study

This study demonstrated that chemical systems inspired by the conformations of pathological and non-pathological proteins can effectively modulate and investigate the amyloid misfolding and aggregation mechanisms of tau. By utilizing a non-natural β^2^-amino acid, it is possible to stabilize β-hairpin-like or extended conformations in peptide sequences. The extended conformation facilitated tau aggregation in the absence of traditional additives like heparin, simulating a seed-competent monomer system, while the β-hairpin conformation preserved tau in a soluble monomeric state, preventing aggregation and potentially preserving microtubule interaction. Despite its significant findings, the study has limitations, such as reliance on *in vitro* models that may not fully capture the complexity of tau aggregation *in vivo*. Additionally, further characterization of tau’s secondary structure during early misfolding stages is necessary. Nevertheless, this work provides robust evidence that peptidomimetic foldamers can be employed to modulate tau aggregation both *in vitro* and in living neuron models.

## Resource availability

### Lead contact

Requests for further information and resources should be directed to and will be fulfilled by the lead contacts, Nicolo Tonali (nicolo.tonali@cea.fr) and Maria-Luisa Gelmi (marialuisa.gelmi@unimi.it).

### Materials availability

Pertinent experimental materials are listed in the key resources table and individual STAR methods sections. All unique/stable reagents generated in this study (compounds **1**, **2** and **3**) are available from the lead contacts with a completed materials transfer agreement.

### Data and code availability

• The published article and supplemental information include all data generated and analyzed during this study.
• CCDC 2363965 contains the supplementary crystallographic data for this paper. The data is available from the Cambridge Crystallographic Data Center via www.ccdc.cam.ac.uk/structures.
• Deposited Data can be found in the OSF datatype-specific repository under this https://doi.org/10.17605/OSF.IO/WAPSH.
• Any additional information required to reanalyze the data reported in this paper is available from the lead contact upon request.

## Acknowledgments

This research has received funding from the European Union’s Horizon 2020 research and innovation program H2020-MSCAITN- 2019-EJD: Marie Skłodowska-Curie Innovative Training Networks (European Joint Doctorate)—Grant Agreement No: 860070—TubInTrain.

Karine Leblanc is thanked for her contribution to the LC-MS analysis.

## Author contributions

The manuscript was written through contributions of all authors. D.D.L., M.-L.G., and N.T. designed the experiments. D.D.L. performed the synthesis, the conformational analysis of NMR, IR, and CD, and the ThT fluorescence assays; N.B. performed and analyzed MTT assays and quantitative live-cell imaging and produce tau protein; I.H. and V.D. performed TEM imaging; L.L.P. performed the X-ray analysis; R.B.R. supervised N.B. for the MTT assays and quantitative live-cell imaging; R.B.U., N.T., and M.-L.G. supervised D.D.L. for the synthesis and the conformational analysis. S.O. revised the manuscript. N.T. and M.-L.G. supervised the entire research.

All authors have given approval to the final version of the manuscript.

## Declaration of interests

The authors declare no competing interests.

## STAR★Methods

### Key resources table

**Table:** 

REAGENT or RESOURCE	SOURCE	IDENTIFIER
**Chemicals, peptides, and recombinant proteins**		

Serum-DMEM (DMEM supplemented with 10% fetal bovine serum)	DMEM: PAN Biotech Serum: gibco	Cat#P04-03550; 10270-106
Lipofectamine 2000	Thermo-Fisher Scientific	Cat#11668027
Serum-reduced DMEM (DMEM supplemented with 1% fetal bovine serum)	DMEM: PAN Biotech Serum: gibco	Cat#P04-03550; 10270-106
100 ng/ml 7S mouse NGF	Alomone Labs, Israel	Cat #: *N*-130
pET-3d-tau plasmids expressing human Tau protein (Wt-Tau)	Eidenmüller et al. (2000) Biochem. 39:13166-75	https://doi.org/10.1021/bi001290z
Escherichia coli BL21(DE3)pLysS	Brandt and Lee (1993) JBC 268:3414-9	https://doi.org/10.1016/S0021-9258(18)53710-8
Penicillin/streptomycin	Thermo-Fisher Scientific	Cat#15070-063
Tris	Sigma Aldrich	CAS: 77-86-1
EDTA	Sigma Aldrich	CAS: 60-00-4
PMSF	Sigma Aldrich	CAS: 329-98-6
DTT	Sigma Aldrich	CAS: 3483-12-3
DNAse I	Thermo-Fisher Scientific	Cat#NC1839861
DE52 (Diethylaminoethyl Cellulose)	Whatman	Cat#4057050
Bactotryptone	Thermo-Fisher Scientific	Cat#211705
Triton X-100	Sigma Aldrich	Cat#T8787
Chloramphenicol	Sigma Aldrich	CAS: 56-75-7
IPTG	Sigma Aldrich	CAS: 367-93-1
Rifampicin	Sigma Aldrich	CAS: 13292-46-1
Optimem	Thermo-Fisher Scientific	Cat#31985062
DMSO	Sigma Aldrich	CAS: 67-68-5
MPMS	Sigma Aldrich	CAS: 65162-13-2
3-(4,5-dimethylthiazol-2-yl)-2,5-diphenyltetrazolium bromide	Thermo-Fisher Scientific	Cat#M6494
INT	Sigma Aldrich	CAS: 146-68-9
PMS	Sigma Aldrich	CAS: 299-11-6
NAD	Sigma Aldrich	CAS: 53-84-9
Lactic acid	Sigma Aldrich	CAS: 79-33-4
PBS pH 7.4 (1X)	Thermo-Fisher Scientific	Cat#10010-015
Methyl 2-(bromomethyl)acrylate	Sigma-Aldrich	CAS: 4224-69-5
Sodium azide	Sigma-Aldrich	CAS: 26628-22-8
Benzhaldehyde oxyme	Sigma-Aldrich	CAS: 622-31-1
N-Chlorosuccinimide	Sigma-Aldrich	CAS: 128-09-6
Triethylamine	Sigma-Aldrich	CAS: 121-44-8
Lithium hydroxide monohydrate	Sigma-Aldrich	CAS: 1310-66-3
HCl·H-Val-OMe	Bachem	CAS: 6306-52-1
Propylphosphonic anhydride solution in ethyl acetate (≥50 wt. %)	Sigma-Aldrich	CAS: 68957-94-8
N,N-Diisopropylethylamine	Sigma-Aldrich	CAS: 7087-68-5
Trimethylphosphine 1M in Toluene	Sigma-Aldrich	CAS: 594-09-2
Fmoc-OSu	Sigma-Aldrich	CAS: 82911-69-1
Rink Amide resin (100-200 mesh)	BLDpharm	CAS: 183599-10-2
N,N’-Diisopropylcarbodiimide	Sigma-Aldrich	CAS: 693-13-0
Oxyma Pure	Sigma-Aldrich	CAS: 3849-21-6
Piperidine	Sigma-Aldrich	CAS: 110-89-4
Acetic anhydride	Sigma-Aldrich	CAS: 108-24-7
Trifluoroacetic acid	Sigma-Aldrich	CAS: 76-05-1
Triisopropylsilane	Sigma-Aldrich	CAS: 6485-79-6
Thioanisole	Sigma-Aldrich	CAS: 100-68-5
HCl·H-Thr(OtBu)-OMe	Bachem	CAS: 69320-90-7
1,1,1,3,3,3-Hexafluoro-2-propanol	Sigma-Aldrich	CAS: 920-66-1
Sodium phosphate dibasic	Sigma-Aldrich	CAS: 7558-79-4
Sodium phosphate monobasic	Sigma-Aldrich	CAS: 7558-80-7
Ethylenediaminetetraacetic acid	Sigma-Aldrich	CAS: 60-00-4
Hydrochloric acid (37%)	Sigma-Aldrich	CAS: 7647-01-0
Uranyl Acetate	Electron Microscopy Sciences	CAS: 541-09-3
Thioflavin T	Sigma-Aldrich	CAS: 2390-54-7
Heparin sodium salt from porcine intestinal mucosa H-3149, average MW 18 kDa	Sigma-Aldrich	CAS: 9041-08-1
n-Hexane	Sigma-Aldrich	CAS: 110-54-3
Ethyl acetate	Sigma-Aldrich	CAS: 141-78-6
Tetrahydrofuran	Sigma-Aldrich	CAS: 109-99-9
Acetonitrile (≥99.9%, gradient grade, suitable for HPLC)	Sigma-Aldrich	CAS: 75-05-8
Water for HPLC	Agilent	CAS: 7732-18-5
Deuterium oxide	Sigma-Aldrich	CAS: 7789-20-0
Dichloromethane	Sigma-Aldrich	CAS: 75-09-2
Fmoc-Lys(Boc)-OH	Bachem	CAS: 71989-26-9
Fmoc-Thr(OtBu)-OH	Bachem	CAS: 71989-35-0
Fmoc-Val-OH	Bachem	CAS: 68858-20-8
Fmoc-Ile-OH	Bachem	CAS: 71989-23-6
Fmoc-Leu-OH	Bachem	CAS: 35661-60-0
Fmoc-Tyr(tBu)-OH	Bachem	CAS: 71989-38-3
Fmoc-His(Trt)-OH	Bachem	CAS: 109425-51-6
Fmoc-Gln(Trt)-OH	Bachem	CAS: 132327-80-1
Fmoc-Asn(Trt)-OH	Bachem	CAS: 132388-59-1

**Deposited data**		

Crystal structure of compound **11b**	Cambridge Crystallographic Data Centre	CCDC 2363965
NMR, ThT fluorescence spectroscopy, MTT, FDAP	Open Science Framework storage	https://doi.org/10.17605/OSF.IO/WAPSH

**Experimental models: Cell lines**		

PC 12 cells	Brandt et al. (1995) J Cell Biol 13:1327–40	https://doi.org/10.1083/jcb.131.5.1327

**Recombinant DNA**		

Tau441wt	Eidenmüller et al. (2000) Biochem. 39:13166-75	https://doi.org/10.1021/bi001290z
Tau441wt dk280	Pinzi et al. (2024) Nat Commun **15**, 1679	https://doi.org/10.1038/s41467-024-45851-6

**Software and algorithms**		

GraphPad Prism v8.0.1	Prism GraphPad	https://www.graphpad.com/scientific-software/prism/
MATLAB	MATLAB - MathWorks	https://it.mathworks.com/products/matlab.html
TopSpin	Bruker	http://bruker.com/en/products-and-solutions/mr/nmr-software/topspin.html
ChemDraw Ultra 12.0	PerkinElmer	https://www.perkinelmer.com/category/chemdraw
R	The R Project for Statistical Computing	https://www.r-project.org/

**Other**		

Silica gel (230–400 mesh)	Sigma Aldrich	CAS: 112926-00-8
TLC silica gel plates 60 F250 (0.26 mm thickness)	Merck	Code UNSPSC: 41115711

### Experimental model and study participant details

#### Cell lines

PC12 cells (originally obtained from J.A. Wagner, Harvard Medical School) were cultured in serum-DMEM (DMEM supplemented with 10% fetal bovine serum and antibiotics (100 U/ml penicillin and 100 μg/mL streptomycin)), at 37°C with 10% CO_2_ in a humidified incubator and transfected with Lipofectamine 2000 (Thermo-Fisher Scientific, USA) as previously described.^55^ After transfection, the medium was replaced with serum-reduced DMEM (DMEM supplemented with 1% fetal bovine serum and antibiotics) and the cells were neuronally differentiated for 4 days by the addition of 100 ng/ml7SmouseNGF (AlomoneLabs, Israel).^48^

#### Cell culture and transfection

Prokaryotic expression plasmids were based on human adult Tau (Tau441wt) in a pET-3d vector.^55^^,^^56^ pET-3d-tau plasmids expressing human Tau protein (Wt-Tau), were transformed into *Escherichia coli* BL21(DE3)pLysS cells for expression.

### Method details

#### General procedures in chemistry

Usual solvents were purchased from commercial sources. Thin-layer chromatography (TLC) analyses were performed on silica gel 60 F250 (0.26 mm thickness) plates. The plates were visualized with UV light (λ = 254 nm) alternatively stained with a 4 % solution of phosphomolybdic acid or ninhydrin in EtOH.

NMR spectra of intermediates were recorded on an ultra-field Bruker AVANCE 300 (^1^H, 300 MHz, ^13^C, 75 MHz) or on a Bruker AVANCE 400 (^1^H, 400 MHz, ^13^C, 100 MHz); NMR spectra of **1**, **2** and **3** were registered with a Bruker spectrometer operating at 700 MHz equipped with cryoprobe. Chemical shifts δ are in ppm with the solvent resonance as the internal standard (^1^H NMR, CDCl3: δ = 7.26 ppm, CD_3_OD: δ = 3.31 ppm, CD_3_CN: δ = 1.93 ppm; ^13^C NMR, CDCl_3_: δ = 77.16 ppm, CD_3_OD: δ = 49.00 ppm; CD_3_CN: δ = 1.3 ppm), and the following abbreviations are used: singlet (s), doublet (d), doublet of doublet (dd), triplet (t), quintuplet (qt), multiplet (m), broad multiplet (brm), and broad singlet (brs), broad doublet (brd). Mass spectra were obtained using a Bruker Esquire electrospray ionization apparatus. HRMS were obtained using a TOF LCT Premier apparatus (Waters) with an electrospray ionization source. The purity of compounds was determined by HPLC-MS on Agilent 1260 Infinity. Column: ATLANTIS T3 column (C18, 2.1 x 150mm-3μm), mobile phase: ACN/H_2_O + 0.1% TFA (gradient 1-30% in 15 or 20 min). Preparative HPLC was performed on Agilent 1260 Infinity II. Column: Pursuit (C18 10 x 250μm-5μm), mobile phase: ACN/H_2_O + 0.1% formic acid (FA) and on Waters XBridge BEH300 (C18, 2.1 x 150mm-5μM); POROSHELL 120 column (C18, 2.1 x 50mm-1.9μm); mobile phase: ACN/H_2_O + 0.1% formic acid (FA) or 0.1% Trifluoroacetic acid (TFA). UPLC-MS analyses were performed on a Waters Acquity UPLC apparatus equipped with a Luna Omega PSC18 Column (1.5 μM, 2.1 x 50 mm) coupled to a single quadrupole EDI-MS (Mictomass ZQ). ESI mass spectra were recorded on an LCQESI MS on a LCQ Advantage spectrometer from Thermo Finningan and a LCQ Fleet spectrometer from Thermo Scientific. Optical rotations were measured on a Perkin–Elmer 343 polarimeter at 20°C (concentration [C] in g/100 mL).

#### Procedure A

**1**, **2**, and **3** were synthesized by microwave-assisted Fmoc/tBu-based solid phase peptide synthesis (SPPS) using an automated synthesizer (Liberty Blue, CEM). Rink-Amide resin (0.55 mmol/g loading) was used as solid support and the synthesis was carried out on a 0.1 mmol scale. All used amino acids were N-terminally Fmoc-protected, while the side chains of trifunctional amino acids were protected with orthogonal, acid labile groups. The coupling was performed using 5 equivalents (eq.) of the protected amino acid, previously dissolved in DMF to obtain a 0.2 M solution. As coupling reagents, 5 eq. of DIC (0.5 M in DMF) and 5 eq. of Oxyma Pure (1 M in DMF) have been used. To remove the Fmoc group, a solution of piperidine in DMF (20% v/v) has been applied. The coupling reaction has been accomplished at 25°C for 120 s, followed by 480 s at 50°C and 35 W. The Fmoc group was cleaved with a standard deprotection protocol at 75°C, 155 W for 15 s followed by 60 s at 90°C, 50 W. The coupling of the synthetic scaffold was performed directly on resin, using as coupling system DIC/Oxyma Pure/DMF (2/2/eq.) and running it overnight at room temperature. Finally, the peptidyl-bound resins were cleaved with a mixture of using 4 mL of acidic cocktail (trifluoracetic acid/water/thioanisol/triisopropyl silane; 95/2.5/1.25/1.25%) for 2 h under continuous shaking.

#### Procedure B

The methyl ester derivative compound (1 eq.) was suspended in dry THF (1M) in a round bottom flask equipped with a magnetic stirrer. Afterward, LiOH 0,1 M in H2O (1.5 eq.) was added, and the reaction was left to stir for 30 min at room temperature. At the end of the reaction, checked by TLC (hexane/EtOAc = 7:3), the solvent was removed by reduced pressure and then the aqueous solution was acidified with 10% HCl (checking the pH with litmus paper till pH < 5) and extracted with ethyl acetate (3 x 15 mL). The combined organic phases were then dried over Na_2_SO_4_, and the solvent was removed under reduced pressure, affording a white solid in quantitative yield.

#### Procedure C

In a round bottom flask equipped with a magnetic stirrer, the carboxylic acid (1 eq.) was suspended in dry DCM (0.1M), then the solution was cooled to 0°C. Afterward, HCl·H-AA-OMe (1.5 eq.) and propanephosphonic acid anhydride (T3P) solution 50% in EtOAc (3 eq.) were added. Finally, DIPEA was added until pH = 8 (generally 5/6 eq.). The reaction was stirred overnight at room temperature. After 12 h, the reaction mixture was successively washed with 5% aqueous solution of KHSO_4_ (20 mL), aqueous solution of NaHCO_3_ (20 mL), and brine (25 mL). The isolated organic layer was dried over Na_2_SO_4_ and concentrated under reduced pressure, and the crude product was purified by flash chromatography with the appropriate eluents.

#### Procedure D

In a round bottom flask equipped with a magnetic stirrer, azido derivative compound (1 eq.) was dissolved in THF (0.1M). Afterward, H2O (7 eq.) and PMe_3_ 1M in Toluene (1.1 eq.) were added to the solution. The reaction mixture turned from colourless to matt white. The reaction was stirred for 16h at room temperature. At the end of the reaction, checked by TLC (hexane/EtOAc = 7:3) and stained with ninhydrin, the mixture was filtered over cotton to remove a part of the white solid of trimethylphosphine-oxide formed during the reaction. After concentration under reduced pressure, the isolated amine obtained was directly in the next step.

#### Procedure E

In a round bottom flask equipped with a magnetic stirrer, the amine derivative compound (1 eq.) was dissolved in DCM (0.1M), and the solution was cooled to 0°C. Afterward, Fmoc-OSu (1.1 eq.) was added, and the pH was checked with litmus paper. The pH must be basic, around 8.5, but not higher to limit the Fmoc-cleavage. If it was found to be acid, it was necessary to add DIPEA (1 eq.) and the pH had to be rechecked. The reaction was stirred for 1 h at 0°C and then for 4 h at r.t. At the end of the reaction, checked by TLC (DCM/MeOH + AcOH = 95:5 + 1%), silica was directly added to the mixture, the solvent was removed and the crude was purified by flash chromatography.

**Compound 1:** *(S)-N1-((4S,7S,10S,13S,14S)-7-(2-amino-2-oxoethyl)-1-((S)-5-(((3S,6S,9S,12S,15S,18S)-22-amino-6-(3-amino-3-oxopropyl)-9-((S)-sec-butyl)-18-carbamoyl-15-(4-hydroxybenzyl)-12-isopropyl-2-methyl-4,7,10,13,16-pentaoxo-5,8,11,14,17-pentaazadocosan-3-yl)carbamoyl)-3-phenyl-4,5-dihydroisoxazol-5-yl)-4-(4-aminobutyl)-10-((S)-sec-butyl)-14-methyl-3,6,9,12-tetraoxo-2,5,8,11-tetraazahexadecan-13-yl)-2-((S)-2-amino-3-methylbutanamido)pentanediamide*

**Figure:**
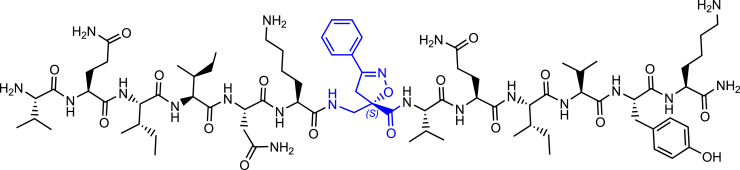

Peptidomimetic **1** was synthesized accordingly to **General procedure A**.

The peptide was purified by semi-preparative HPLC (Linear gradients of 20-70% ACN in H2O containing 0.1% TFA in 20 min, yield isolation: 50%).

Molecular weight: 1644.9715 g/mol.

**HRMS:** Calcd. for [C_79_H_128_N_20_O_18_ + H]^+^: m/z 1645.9788 found 823.4954 [M+2H]^2+^ and 834.4862 [M+H+Na]^2+^

**HPLC purity:** XSELECT column (C18, 2.1 x 75mm-2.5μm); (Linear gradients of 5-100% ACN in H_2_O containing 0.1% TFA in 20 min); Rt = 4.792 min, 100 %.

The LC-MS, HRMS and ^1^H- and ^13^C-NMR chemical shifts are in the supplemental information (PDF1), Figure S14 and Table S1.

**Compound 2:** *(S)-N1-((4S,7S,10S,13S,14S)-7-(2-amino-2-oxoethyl)-1-((R)-5-(((3S,6S,9S,12S,15S,18S)-22-amino-6-(3-amino-3-oxopropyl)-9-((S)-sec-butyl)-18-carbamoyl-15-(4-hydroxybenzyl)-12-isopropyl-2-methyl-4,7,10,13,16-pentaoxo-5,8,11,14,17-pentaazadocosan-3-yl)carbamoyl)-3-phenyl-4,5-dihydroisoxazol-5-yl)-4-(4-aminobutyl)-10-((S)-sec-butyl)-14-methyl-3,6,9,12-tetraoxo-2,5,8,11-tetraazahexadecan-13-yl)-2-((S)-2-amino-3-methylbutanamido)pentanediamide*

**Figure:**
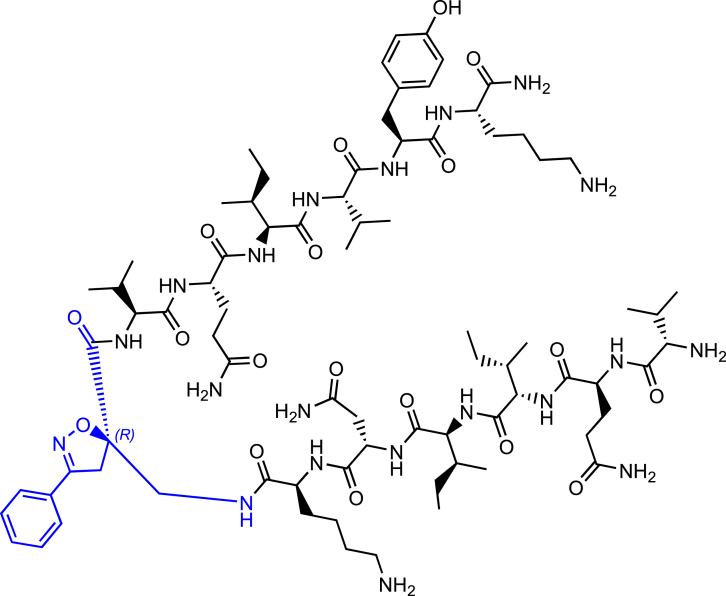

Peptidomimetic **2** was synthesized according to **General Procedure A**.

The peptide was purified by semi-preparative HPLC (Linear gradients of 20-70 % ACN in H2O containing 0.1% TFA in 20 min, yield isolation: 50%).

Molecular weight: 1644.9715 g/mol.

**HRMS:** Calcd. for [C_79_H_128_N_20_O_18_ + H]^+^: m/z 1645.9788 found 1645.9816 [M + H]^+^; Calcd. for [C_79_H_129_N_20_O_18_ + Na]^+^: m/z 1667.9608 found 1667.9594 [M + Na]^+^ and 823.4954 [M + 2H]^2+^

**HPLC purity:** XSELECT column (C18, 2.1 x 75mm-2.5μm); (Linear gradients of 5-100% ACN in H_2_O containing 0.1% TFA in 20 min); Rt = 5.043 min, 100 %.

The LC-MS, HRMS and ^1^H- and ^13^C-NMR chemical shifts are in the supplemental information (PDF1), Figure S15 and Table S2.

**Compound 5:** Methyl 2-(azidomethyl)acrylate.

**Figure:**
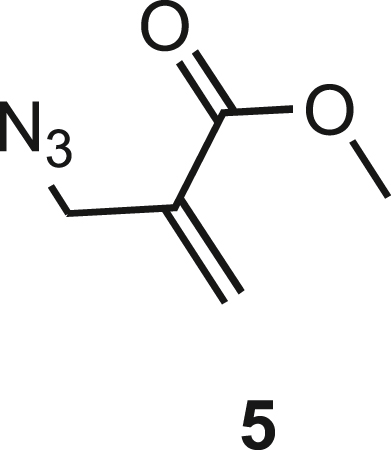

In a round bottom flask equipped with a magnetic stirrer, **4** (74 mg, 4.13 mmol) was suspended in a mixture of (CH_3_)_2_CO and H_2_O 3:1 (16 mL). NaN_3_ (537 mg, 8.26, 2 eq.) was added to the solution. The reaction was stirred for 4 hours at room temperature. The reaction color turned slowly from colourless to pale orange. At the end of the reaction, the mixture was diluted with CH_2_Cl_2_ (15 mL) and the organic layer was extracted and washed successively with H_2_O (5 mL), brine (5 mL) and then dried over Na_2_SO_4_. The solvent was removed under reduced pressure, affording **5** as a pale-yellow oil with 98% yield.

**R*f*** (hexane/AcOEt 9:1) = 0.44.

^**1**^**H NMR (300 MHz, CDCl3)**: δ 6.38-6.35 (1H, m), 5.85-5.83 (1H, m), 4.04 (2H, s), 3.79 (3H, s);

^**13**^**C NMR: (75 MHz, CD3Cl)**: δ 165.7, 134.9; 128.1; 52.2; 51.4.

The NMR analysis is already reported in literature.^57^

**Compound 7:** N-hydroxybenzimidoyl chloride.

**Figure:**
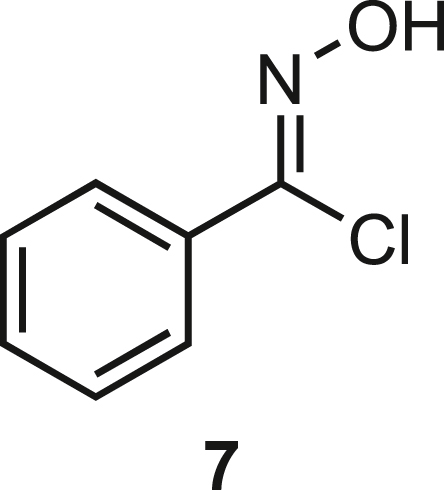

In a two-necked round-bottom flask, equipped with magnetic stirrer and nitrogen inlet, oxime **6** (500 mg, 4.13 mmol) was suspended in dry DMF (6 mL). Afterwards, NCS (551 mg, 4.13 mmol, 1 eq.) was added to the solution. The mixture turned quickly from colourless to bright yellow, and finally pale yellow. The reaction was stirred for 4 hours at room temperature under nitrogen atmosphere. The reaction mixture was diluted with CH_2_Cl_2_ (15 mL) and the organic layer was extracted and washed with H_2_O (10 mL), then dried over Na_2_SO_4_. The solvent was removed under reduced pressure to afford **7** in quantitative yield as a pale-yellow oil, which was used in the next step without further purification.

The NMR analysis are already reported in literature.^58^

^**1**^**H-NMR (300 MHz, CDCl**_**3**_**)**: δ 7.85-7.35 (5H, m), 8.43 (1H, s).

**Compound 9:** Methyl 5-(azidomethyl)-3-phenyl-4,5- dihydroisoxazole-5-carboxylate.

**Figure:**
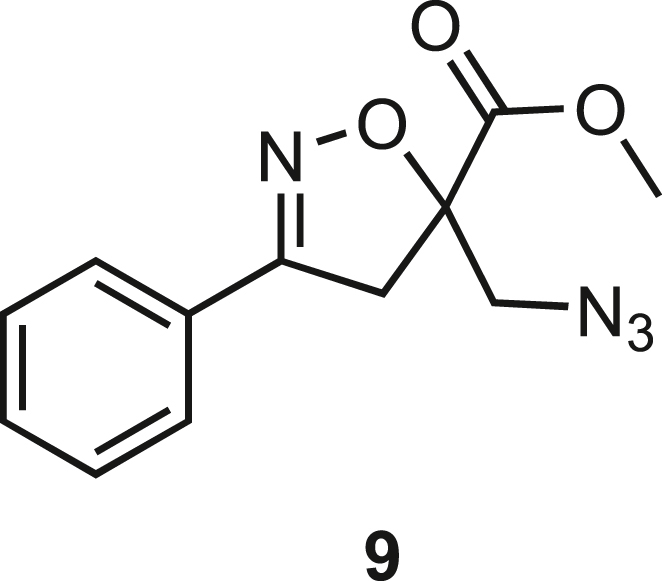

In a two-necked round-bottom flask equipped with a magnetic stirrer and nitrogen inlet, **5** (640 mg, 4.13 mmol, 1 eq.) was suspended in dry THF (5 mL). Afterward, **7** (580 mg, 4.13 mmol, 1 eq.) was diluted in dry THF (7 mL) and added dropwise to the solution. TEA (1,15 mL, 8.26 mmol, 2 eq.) was added dropwise to the mixture, and a white solid precipitate was formed. The reaction was stirred overnight at room temperature under a nitrogen atmosphere. After the concentration of the mixture under reduced pressure, the residue obtained was taken up with AcOEt (10 mL) and washed with H_2_O (15 mL). The organic layer was then dried over Na_2_SO_4_, filtered, and the solvent removed under reduced pressure to give a dark yellow oil, which was purified by chromatography on silica gel eluting with Hexane/AcOEt 8:2 to afford **9** (859 mg, 3.304 mmol, 80%) as a white solid.

Molecular weight: 260.09 g/mol.

**R*f*** (hexane/AcOEt 8:2) = 0.4.

**MS**: calcd. for [C_12_H_12_N_4_O_3_ + H]^+^: m/z 261.0982, found: 261.09 [M + H]^+^ and [C_12_H_12_N_4_O_3_ + Na]^+^: m/z 283.0802; found: 283.1 [M + Na]^+^

^**1**^**H NMR (300 MHz, CDCl**_**3**_**)** : δ 7.70-7.65 (2H, m), 7.54-7.35 (3H, m), 3.87 (3H, s), 3.83-3.76 (2H, m), 3.67 (1H, d, *J =* 13 Hz), 3.52 (1H, d, *J =* 17.2 Hz).

^**13**^**C NMR (75 MHz, CDCl**_**3**_**)** : δ 170.1, 156.4, 130.8, 128.9, 128.4, 127.0, 87.6, 53.4, 44.2, 41.5.

**Compound 9-OH:** 5-(Azidomethyl)-3-phenyl-4,5-dihydroisoxazole-5-carboxylic acid.

**Figure:**
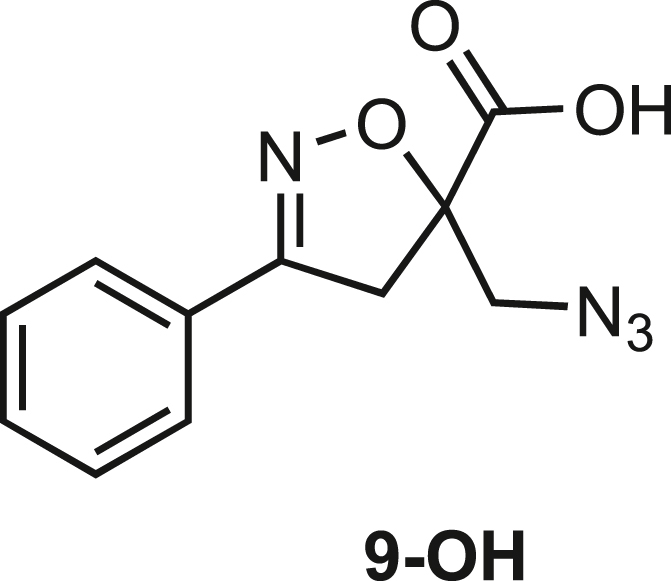

**9-OH** was obtained as a white solid in quantitative yield following the general procedure **B** starting from **9** (980mg, 3.77 mmol)

Molecular weight: 246.08 g/mol.

**R*f*** (Hex/EtOAc 7/3) = 0.

**MS**: Calcd. for [C_11_H_10_N_4_O_3_ + H]^+^: m/z 247.0826 found: 247.08 [M + H]^+^

^**1**^**H NMR (300 MHz, CDCl**_**3**_**)**: δ 9.77 (s, 1H), 7.73–7.61 (2H, m), 7.54 –7.38 (3H, m), 3.85 (1H, d, *J =* 13.11 Hz), 3.77–3.67 (2H, m), 3.58 (1H, d, *J =* 17,38 Hz).

^**13**^**C NMR (75 MHz, CDCl**_**3**_**)**: δ 173.60, 156.91, 131.03, 128.93, 127.89, 127.03, 87.52, 43.97, 41.75.

**Compound 10a:** Methyl ((S)-5-(azidomethyl)-3-phenyl-4,5-dihydroisoxazole-5-carbonyl)-L-valinate

**Compound 10b:** Methyl ((R)-5-(azidomethyl)-3-phenyl-4,5-dihydroisoxazole-5-carbonyl)-L-valinate

**10a** and **10b** were obtained following the general procedure **C** starting from **9a** (927 mg, 3.77 mmol). The crude product was purified by chromatography on silica gel eluting with DCM/Et_2_O 98:2 to yield **10a** and **10b** (1.3 g, 3.66 mmol, overall yield 97%) as white solids isolated in 42% and 47% yield, respectively.

**Figure:**
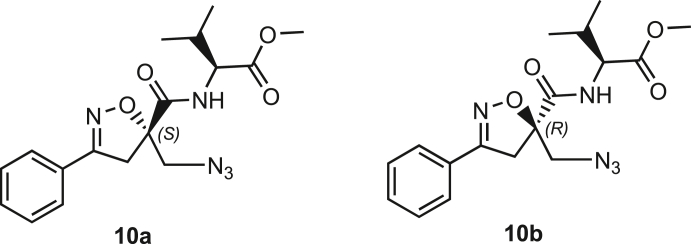

#### **Molecular weight**: 359.16 g/mol

**(10a) R*f*** (DCM/Et_2_O = 98/2) = 0.3.

**(10a) [α]**_**20**_^**D**^: +15° ([c] = 0.001 in CH_2_Cl_2_)

**(10b) R*f*** (DCM/Et_2_O = 98/2) = 0.2.

**(10b) [α]**_**20**_^**D**^: -18.8° ([c] = 0.005 in CH_2_Cl_2_)

**MS**: Calcd. for [C_17_H_21_N_5_O_4_ + H]^+^: m/z 360.1666; found: 359.98 [M + H]^+^ and 740.83 [2M + Na]^+^

**(10a)**^**1**^**H NMR (300 MHz, CDCl**_**3**_**)**: δ 7.68 (2H, m), 7.46, 7.44 (3H, m), 7.35 (1H, d, *J* = 8.9 Hz); 4.54 (1H, dd, *J* = 9.0 Hz and 5.0), 3.84 (1H, d, *J* = 13.0 Hz), 3.71 (3H, s), 3.70 (1H, d, J = 17.53 Hz), 3.67 (1H, d, *J* = 13.1 Hz), 3.52 (1H, d, *J* = 17.53 Hz), 2.26 (1H, m), 1.01 (6H, m).

**(10a)**^**13**^**C NMR (101 MHz, CDCl**_**3**_**)**: δ 171.35, 170.47, 157.47, 130.95, 128.89, 128.18, 127.00, 89.20, 57.34, 55.14, 52.24, 42.42, 31.20, 18.98, 17.72.

**(10b)**^**1**^**H NMR (400 MHz, CDCl**_**3**_**)**: δ 7.75–7.61 (2H, m), 7.5 –7.41 (3H, m), 7.38 (1H, d, *J* = 9.1 Hz), 4.50 (1H, dd, *J* = 9.1Hz and 5.1), 3.88 (1H, d, *J* = 13.3 Hz), 3.65 (3H, s), 3.70 (1H, d, J = 17.49 Hz); 3.65 (1H, d, *J* = 13.3 Hz); 3.62 (1H, d, *J* = 17.45 Hz), 2.29–2.11 (1H, m), 0.90 (6H, d, *J* = 6.9 Hz).

**(10b)**^**13**^**C NMR (101 MHz, CDCl**_**3**_**)**: δ 171.34, 170.76, 157.77, 130.99, 128.94, 128.21, 126.97, 89.39, 57.30, 54.29, 52.33, 42.01, 31.05, 18.97, 17.69.

**Compound 11a:** ((S)-5-(Azidomethyl)-3-phenyl-4,5-dihydroisoxazole-5-carbonyl)-L-valine.

**Figure:**
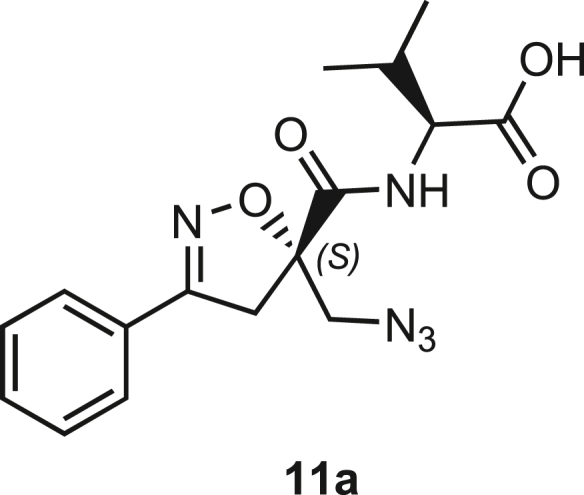

**11a** (193.2 mg, 0.56 mmol, quant.) was obtained as a white solid following the general procedure **B** from **10a** (201 mg, 0.56 mmol).

Molecular weight: 345.14 g/mol.

White solid.

**R*f*** (Hex/EtOAc 7/3) = 0.

**MS**: Calcd. for [C_16_H_18_N_4_O_3_ - H]^-^: m/z 344.1359; found: 344.44 [M - H]^-^

^**1**^**H NMR (300 MHz, CDCl**_**3**_**)**: δ 7.71 (1H, bs), 7.65–7.59 (2H, m), 7.48–7.34 (4H, m), 4.49 (1H, dd, *J* = 8.9 Hz and 4.7 Hz), 3.81 (1H, d, *J* = 13.1 Hz), 3.68 (1H, d, *J* = 17.6 Hz), 3.62 (1H, d, *J* = 13.05 Hz), 3.46 (1H, d, *J* = 17.6 Hz), 2.34–2.20 (1H, m), 0.99 (6H, m).

^**13**^**C NMR (75 MHz, CDCl**_**3**_**)**: δ 175.17, 170.78, 157.65, 131.01, 128.89, 127.96, 127.01, 89.13, 57.20, 55.04, 42.37, 30.90, 18.98, 17.53.

**Compound 11b:** ((R)-5-(Azidomethyl)-3-phenyl-4,5-dihydroisoxazole-5-carbonyl)-L-valine.

**Figure:**
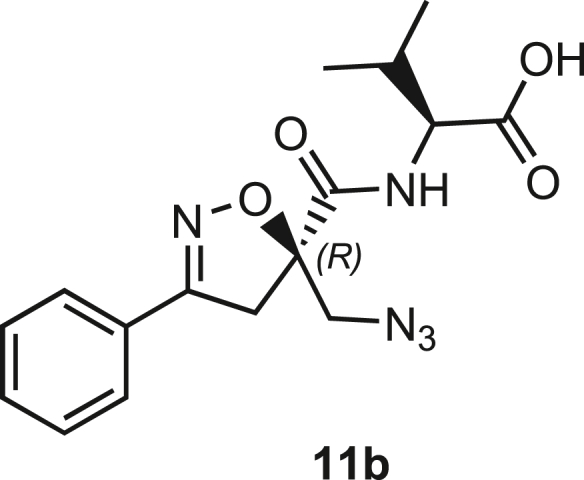

**11b** (548.8 mg, 1.59 mmol, quant.) was obtained as a white solid following the general procedure **B** from 10b. Compound 11b was crystallized by slow evaporation from a solution containing Et2O/Hexane. 11b was dissolved in the lowest amount of Et2O, and a small amount of hexane was added until the appearance of turbidity. After an extremely low evaporation of the solution, without moving the flask, crystals of **11b** were obtained.

**Molecular weight**: 345.14 g/mol;

Rf (Hex/EtOAc 7/3) = 0.

**[α]**_**20**_^**D**^**(3.12b)**: + 6° ([c] = 0.004 in CH_2_Cl_2_);

**MS**: Calcd. for [C_16_H_19_N_4_O_3_ - H]^-^: m/z 344.1359; found: 344.44 [M - H]^-^

^**1**^**H NMR (400 MHz, CDCl**_**3**_**)**: δ 8.58 (1H, bs), 7.69 (2H, dd, *J* = 7.9 Hz and 1.8 Hz), 7.56–7.19 (4H, m), 4.53 (1H, dd, *J* = 8.9 Hz and 4.9 Hz), 3.90 (1H, d, *J* = 13.3 Hz), 3.72 (1H, d, *J* = 17.5 Hz), 3.67 (1H, d, *J* = 13.4 Hz), 3.64 (1H, d, *J* = 17.5 Hz), 2.30 (1H, m, *J* = 6.9 Hz and 4.9 Hz), 0.96 (6H, dd, *J* = 6.9, 3.8 Hz) ppm.

^**13**^**C NMR (101 MHz, CDCl**_**3**_**)**: δ 175.48, 171.21, 157.91, 131.06, 128.96, 128.12, 127.01, 89.37, 57.25, 54.23, 41.97, 30.73, 19.05, 17.58 ppm.

**Compound 11a-NH**_**2**_**:** Methyl ((S)-5-(aminomethyl)-3-phenyl-4,5-dihydroisoxazole-5-carbonyl)-L-valine.

**Figure:**
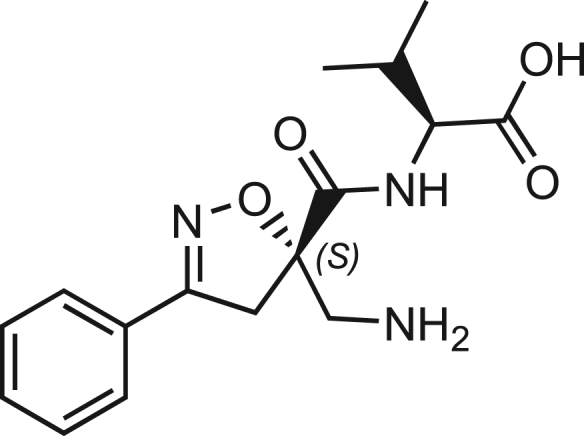

**11a-NH**_**2**_ was obtained as a pale yellow oil following the general procedure **D** starting from **11a** (193 mg, 0.56 mmol). The compound was used for the next synthetic step without any further purification.

**Molecular weight**: 319.15 g/mol;

**R*f*** (Hex/EtOAc 7/3) = 0.

**MS**: Calcd. for [C_16_H_21_N_4_O_3_ + H]^+^: m/z 320.1605; found: 320.23 [M + H]^+^

**Compound 11b-NH**_**2**_**:** Methyl ((R)-5-(aminomethyl)-3-phenyl-4,5-dihydroisoxazole-5-carbonyl)-L-valine.

**Figure:**
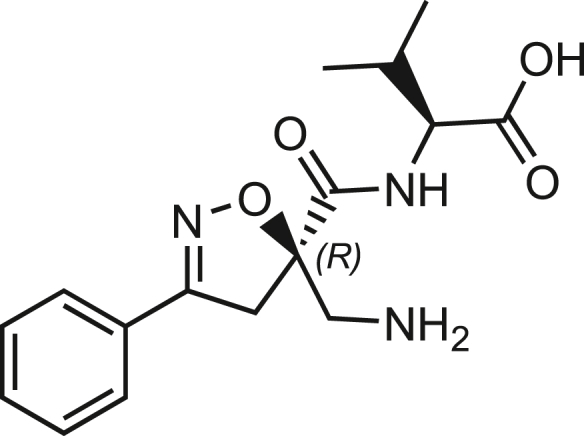

**11b-NH**_**2**_ was obtained as a pale yellow oil following the general procedure **D** starting from **11b** (570 mg, 1.59 mmol). The compound was used for the next synthetic step without any further purification.

**Molecular weight**: 319.15 g/mol;

**R*f*** (Hex/EtOAc 7/3) = 0.

**MS:** Calcd. for [C_16_H_21_N_4_O_3_ + H]^+^: m/z 320.1605; found: 320.23 [M + H]^+^

**Compound 12a:** Methyl ((S)-5-(((((9H-fluoren-9-yl)methoxy)carbonyl)amino)methyl)-3-phenyl-4,5-dihydroisoxazole-5-carbonyl)-L-valine.

**Figure:**
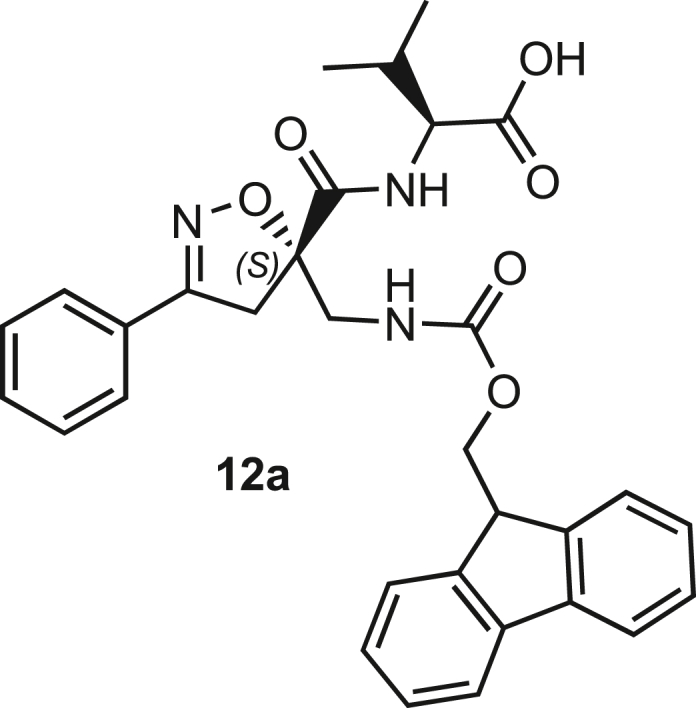

**12a** was obtained following the general procedure **E** from crude **11a-NH**_**2**_ (0.56 mmol). TLC (DCM/MeOH + AcOH = 95:5 + 1%) was used to monitor the reaction. The crude obtained was purified by chromatography on silica gel eluting with DCM/MeOH + AcOH 95/5 + 1% as eluent to afford **12a** (212 mg, 0,392 mmol) as a white spongy solid in 70% yield.

**Molecular weight**: 541.22 g/mol;

**R*f*** (DCM/MeOH + AcOH 95/5 + 1%) = 0.5;

**[α]**_**20**_^**D**^**(3.14a)**: -14° ([c] = 0.002 M in CH_2_Cl_2_);

**MS**: Calcd. for [C_31_H_31_N_3_O_6_ + H]^+^: m/z 542.2286 found: 542.61 [M + H]^+^; Calcd. for [C_31_H_31_N_3_O_6_ + Na]^+^: m/z 564.2105; found: 564.79 [M+Na]^+^ and 1105.90 [2M + Na]^+^;

^**1**^**H NMR (400 MHz, CD**_**3**_**OD)**: δ 7.76 (2H, dd, *J* = 7.6 Hz and 3.6 Hz), 7.72–7.63 (2H, m), 7.59 (2H, d, *J* = 7.5 Hz), 7.51–7.39 (3H, m, H1), 7.34 (2H, t, *J* = 7.5 Hz), 7.22 (2H, dt, *J* = 15.1 Hz and 7.5 Hz), 4.42–4.35 (2H, m), 4.27 (1H, dd, *J* = 10.6 Hz and 6.8 Hz), 4.16 (1H, t, *J* = 6.9 Hz), 3.81–3.64 (3H, m), 3.55 (1H, d, *J* = 17.8 Hz), 2.24 (1H, m), 0.98 (6H, m).

^**13**^**C NMR (101 MHz, CD**_**3**_**OD)**: δ 172.68, 172.04, 157.78, 157.70, 143.81, 143.77, 141.15, 141.15, 130.40, 128.53, 128.50, 127.33, 126.73, 126.66, 124.80, 124.68, 119.48, 119.46, 89.57, 66.64, 57.45, 46.94, 45.15, 41.18, 30.48, 18.13, 16.86.

**Compound 12b:** Methyl ((R)-5-(((((9H-fluoren-9-yl)methoxy)carbonyl)amino)methyl)-3-phenyl-4,5-dihydroisoxazole-5-carbonyl)-L-valine.

**Figure:**
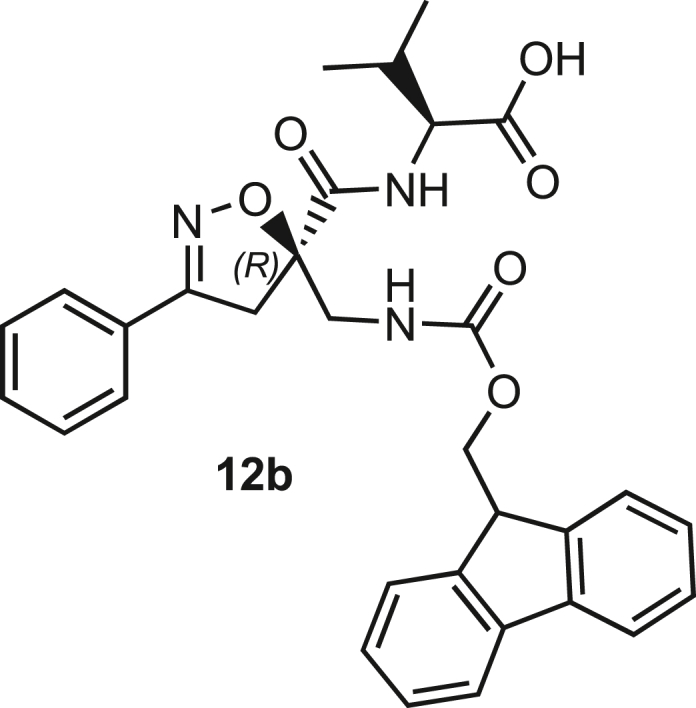

**12b** was obtained following the general procedure **E** from crude **11b-NH**_**2**_ (1.59 mmol). TLC (DCM/MeOH + AcOH = 95:5 + 1%) was used to monitor the reaction. The crude obtained was purified by chromatography on silica gel eluting with DCM/MeOH + AcOH 95/5 + 1% as eluent to afford **12b** (500 mg, 0.97 mmol) as a white spongy solid in 60% yield.

**Molecular weight**: 541.22 g/mol;

**R*f*** (DCM/MeOH + AcOH 95/5 + 1%) = 0.5;

**[α]**_**20**_^**D**^**(3.14b)**: +20° ([c] = 0.005 in CH_2_Cl_2_);

**MS:** Calcd. for [C_31_H_31_N_3_O_6_ + H]^+^: M/z 542.2286 found: 542.61 [M + H]^+^; Calcd. for [C_31_H_31_N_3_O_6_ + Na]^+^: m/z 564.2105; found: 564.79 [M + Na]^+^ and 1105.90 [2M + Na]^+^;

^**1**^**H NMR (400 MHz, CD**_**3**_**OD)**: δ 7.75 (2H, dd, *J* = 7.6, 4.4 Hz), 7.72–7.67 (2H, m), 7.60 (2H, dd, *J* = 7.5 Hz and 4.8 Hz), 7.5 –7.39 (3H, m), 7.33 (2H, t, *J* = 7.5 Hz), 7.28–7.14 (2H, m), 4.43– 4.24 (3H, m), 4.18 (1H, t, *J* = 7.1 Hz), 3.80 (1H, d, *J* = 14.6 Hz), 3.75–3.62 (2H, m), 3.55 (1H, d, *J* = 17.7 Hz), 2.23 (1H, m, *J* = 6.9 Hz and 5.2 Hz), 0.92 (6H, m).

^**13**^**C NMR (101 MHz, CD**_**3**_**OD)**: δ 172.71, 172.19, 158.09, 157.77, 143.90, 143.74, 141.12, 130.52, 128.59, 128.51, 127.32, 126.73, 126.62, 124.81, 124.78, 119.44, 89.65, 66.68, 57.38, 46.96, 44.30, 41.01, 30.49, 18.08, 16.61.

#### Synthetic strategy for compound 3

The two stereoisomers of **9** have been separated after elongating the *C-*terminal peptide sequence. H-Thr(O^t^Bu)-OMe (1.5 eq) has been coupled with the same protocol based on T3P (3 eq) and DIPEA (3 eq), obtaining **1Sa** and **1Sb** with an excellent 93% yield. However, the diastereomeric mixture could not be separated using silica chromatography. To address this problem, we decided to insert an extra stereogenic center at the *C-*terminal chain. The methyl ester was first removed via basic hydrolysis (LiOH, H2O/THF), followed by the coupling with H-Lys(Boc)-OMe using the T3P protocol. Although the coupling afforded a moderate 28% yield, the resulting diastereoisomers, **2Sa** and **2Sb**, resulted separable by flash chromatography (Hex/EtOAc = 6:4) (Scheme S1 and 2).

Diastereoisomer **2Sb** was subjected to modification to make it suitable for the SPPS strategy. First, the methyl ester group was hydrolyzed via classical saponification using LiOH (1.5 eq) in THF/H_2_O, isolating **3Sb** in quantitative yields. Then, the Staudinger protocol was used in the presence of PMe_3_ (7 eq) and H_2_O (49 eq) to reduce the azido group and obtain the amino derivatives **4Sb**, which was then Fmoc-protected (Fmoc-OSu (1.1 eq) in DCM, 4h), affording the final scaffold **5Sb** in satisfactory yields (67%) over two steps (Scheme S2 and 3).

**Compound 3:** *(R)-N-((2S,5S,8S,11S,14S,17S,18S)-2-((1H-imidazol-2-yl)methyl)-1-amino-14-(4-aminobutyl)-8-((S)-sec-butyl)-18-hydroxy-5-isobutyl-11-isopropyl-1,4,7,10,13,16-hexaoxo-3,6,9,12,15-pentaazanonadecan-17-yl)-5-((4S,7S,10S,13S,16S,19S)-19,23-diamino-7-((S)-sec-butyl)-4-((S)-1-hydroxyethyl)-10,13,16-triisopropyl-3,6,9,12,15,18-hexaoxo-2,5,8,11,14,17-hexaazatricosyl)-3-phenyl-4,5-dihydroisoxazole-5-carboxamide.*

**Figure:**
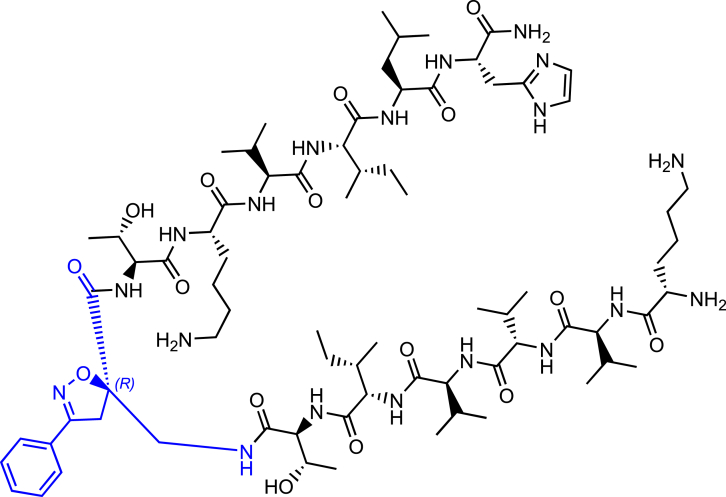

Peptidomimetic **3** was synthesized according to **General Procedure A**.

The peptide was purified by semi-preparative HPLC (Linear gradients of 20-70 % ACN in H_2_O containing 0.1% TFA in 20 min, yield isolation: 50%).

**Molecular weight**: 1549.9708 g/mol.

**HRMS:** Calcd. for [C_75_H_127_N_19_O_16_ + H]^+^: m/z 1550.9781 found 1550.9712 [M + H]^+^; Calcd. for [C_79_H_129_N_20_O_18_ + Na]^+^: m/z 1572.9600 found 1572.9530 [M + Na]^+^ and 775,9936 [M + 2H]^2+^ and 517.6668 [M + 3H]^3+^

**HPLC purity**: XSELECT column (C18, 2.1 x 75mm-2.5μm); (Linear gradients of 5-100% ACN in H_2_O containing 0.1% TFA in 20 min); Rt = 4.81 min, 100 %.

The LC-MS, HRMS and ^1^H- and ^13^C-NMR chemical shifts are in the supplemental information (PDF1), Figure S16 and Table S3.

**Compound 1S (a,b):** *Methyl N-(5-(azidomethyl)-3-phenyl-4,5-dihydroisoxazole-5-carbonyl)-O-(tert-butyl)-L-allothreoninate*

**Figure:**
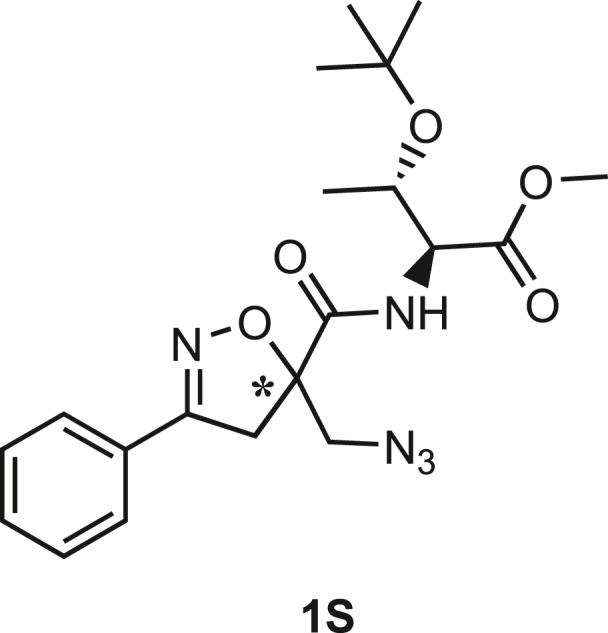

**1S** was obtained as a pale yellow oil following the general procedure **C** starting from **9a** (963 mg, 3.91 mmol, 1 eq.). The crude product was purified by chromatography on silica gel eluting with Hexane/AcOEt to yield **1S** (1.51 g, 3.64 mmol, 93%) as a mixture of inseparable diastereoisomers.

Molecular weight: 417.20 g/mol.

**R*f*** (Hexane/AcOEt = 85:15): 0.3.

**MS**: Calcd. for [C_20_H_27_N_5_O_5_ + H]^+^: M/z 418.2085 found: 418.31 [M + H]^+^

(**1S a**) ^**1**^**H NMR (300 MHz, CDCl**_**3**_**)**: δ 7.75 – 7.65 (2H, m), 7.62 (1H, d, *J* = 9.1 Hz), 7.51 – 7.38 (3H, m,), 4.44 (1H, dd, *J* = 9.1, 2.1 Hz), 4.26 (1H, dd, *J* = 6.3, 2.3 Hz, 1H), 3.91 (1H, d, *J* = 13.3 Hz), 3.73 – 3.64 (2H, m) 3.63 (3H, s), 3.51 (1H, d, *J* = 17.52 Hz), 1.22 (3H, d, *J* = 6.24 Hz), 1.12 (9H, s).

(**1S a**) ^**13**^**C NMR (101 MHz, CDCl**_**3**_**)**: δ 171.09, 170.49, 157.35, 130.82, 128.86, 128.37, 126.96, 89.18, 74.24, 67.09, 58.14, 54.91, 52.23, 42.51, 29.70, 20.95.

(**1S b**) ^**1**^**H NMR (400 MHz, CDCl**_**3**_**)**: δ 7.75 – 7.65 (2H, m), 7.62 (1H, d, *J* = 9.1 Hz), 7.51 – 7.38 (3H, m), 4.41 (1H, dd, *J* = 9.0, 2.3 Hz), 4.26 (1H, dd, *J* = 6.3, 2.3 Hz), 3.91 (1H, d, *J* = 13.3 Hz), 3.76 (3H, s), 3.72 (1H, d, *J* = 18.0 Hz) , 3.66 – 3.60 (2H, m), 1.14 (9H, s), 1.11 (d, *J* = 6.2 Hz).

(**1S b**) ^**13**^**C NMR (101 MHz, CDCl**_**3**_**)**: δ 171.43, 170.46, 157.55, 130.84, 128.88, 128.42, 126.99, 89.46, 74.29, 66.94, 58.30, 54.39, 52.35, 41.63, 28.35, 20.98.

**Compound 2S a** : *Methyl N*^*2*^*-(N-((S)-5-(azidomethyl)-3-phenyl-4,5-dihydroisoxazole-5-carbonyl)-O-(tert-butyl)-L-allothreonyl)-N*^*6*^*-(tert-butoxycarbonyl)-L-lysinate.*

**Compound 2S b** : *Methyl N*^*2*^*-(N-((R)-5-(azidomethyl)-3-phenyl-4,5-dihydroisoxazole-5-carbonyl)-O-(tert-butyl)-L-allothreonyl)-N*^*6*^*-(tert-butoxycarbonyl)-L-lysinate.*

**Figure:**
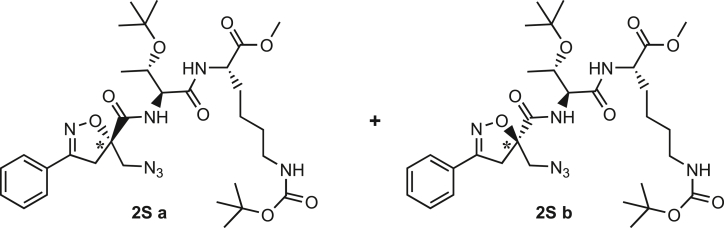

After saponification of **1S** according to procedure **B**, in a round bottom flask equipped with a magnetic stirrer, deprotected **1S** (927 mg, 3.77 mmol, 1 eq.) was suspended in dry DCM (20 mL), then the solution was cooled to 0 °C. At this moment, H-Thr(OtBu)-OMe (947 mg, 5.65 mmol, 1.5 eq) and T_3_P solution 50% in EtOAc (4.10 mL, 8.88 mmol, 3 eq.) were added and secondly DIPEA was added until pH = 8 (22.6 mmol, 5 eq.). After stirring overnight at room temperature, the reaction mixture was successively washed with a 5% aqueous solution of KHSO4 (20 mL), an aqueous solution of NaHCO3 (20 mL), and brine (25 mL). Then, the organic layer was isolated, dried over Na_2_SO_4_, filtered, and concentrated under reduced pressure. The crude pale-yellow oil obtained was purified by chromatography on silica gel eluting with DCM/Et_2_O 98/2 to afford compounds 2Sa (316 mg, 0.5 mmol, 13%) and 2Sb (341 mg, 0.54 mmol, 14%) separately as white solid crystals.

**Molecular weight**: 645.35 g/mol.

**(2S a) R*f*** (DCM/Et_2_O 98/2) = 0.3.

**(2S b) R*f*** (DCM/Et_2_O 98/2) = 0.18.

**MS**: Calcd. for [C_31_H_47_N_7_NaO_8_ + Na]^+^: m/z 668.3378 found: 668.37.

**(2S a)**^**1**^**H NMR (400 MHz, CDCl**_**3**_**)**: δ 7.97 (1H, d, *J* = 5.9 Hz), 7.66 (3H, m), 7.49–7.35 (3H, m), 4.60–4.43 (2H, m), 4.35 (1H, dd, *J* = 5.9 Hz and 4.0 Hz), 4.26 (1H, dd, *J* = 6.4 Hz and 4.0 Hz), 3.84 (1H, d, *J* = 13.0 Hz), 3.76–3.68 (4H, m), 3.64 (1H, d, *J* = 13.0 Hz), 3.45 (1H, d, *J* = 17.5 Hz), 3.08 (2H, m, *J* = 6.4 Hz), 1.90–1.77 (1H, m), 1.7–1.64 (1H, m), 1.43 (11H, s), 1.33 (11H, bs), 1.16 (3H, d, *J* = 6.4 Hz).

**(2S a)**^**13**^**C NMR (101 MHz, CDCl**_**3**_**)**: δ 172.22, 170.48, 168.73, 157.04, 155.89, 130.78, 128.78, 128.30, 126.99, 89.04, 79.10, 75.69, 65.86, 57.44, 55.39, 52.27, 42.26, 40.24, 31.79, 29.65, 28.39, 28.16, 22.45, 16.74.

**(2S b)**^**1**^**H NMR (400 MHz, CDCl**_**3**_**)**: δ 7.91 (1H, d, *J* = 6.2 Hz), 7.74–7.59 (3H, m), 7.52–7.38 (3H, m), 4.56 (2H, m), 4.35 (1H, dd, *J* = 6.3 Hz and 3.8 Hz), 4.16 (1H, m), 3.89 (1H, d, *J* = 13.1 Hz), 3.76 (3H, s), 3.73–3.62 (2H, m,), 3.56 (d, 1H, *J* = 17.5 Hz), 3.12 (2H, q, *J* = 6.4 H), 1.96–1.82 (1H, m), 1.81–1.68 (1H, m), 1.58–1.48 (2H, m), 1.45 (9H, s), 1.29 (11H, s), 1.02 (3H, d, *J* = 6.4 Hz).

**(2S b)**^**13**^**C NMR (101 MHz, CDCl**_**3**_**)**: δ 172.27, 170.76, 168.81, 157.20, 155.93, 130.86, 128.89, 128.34, 126.94, 89.16, 79.14, 75.51, 65.98, 57.59, 54.65, 52.31, 52.29, 41.95, 40.27, 31.87, 29.69, 28.42, 28.21, 22.54, 17.26.

**Compound 3S b:** N^2^-(N-((R)5-(azidomethyl)-3-phenyl-4,5-dihydroisoxazole-5-carbonyl)-O-(tert-butyl)-L-allothreonyl)-N^6^-(tert-butoxycarbonyl)-L-lysine.

**3S b** (341 mg, 0.54 mmol, quant.) was obtained following the general procedure **B** starting from **2S b** (341 mg, 0.54 mmol, 1 eq.) as a white solid in quantitative yield.

**Figure:**
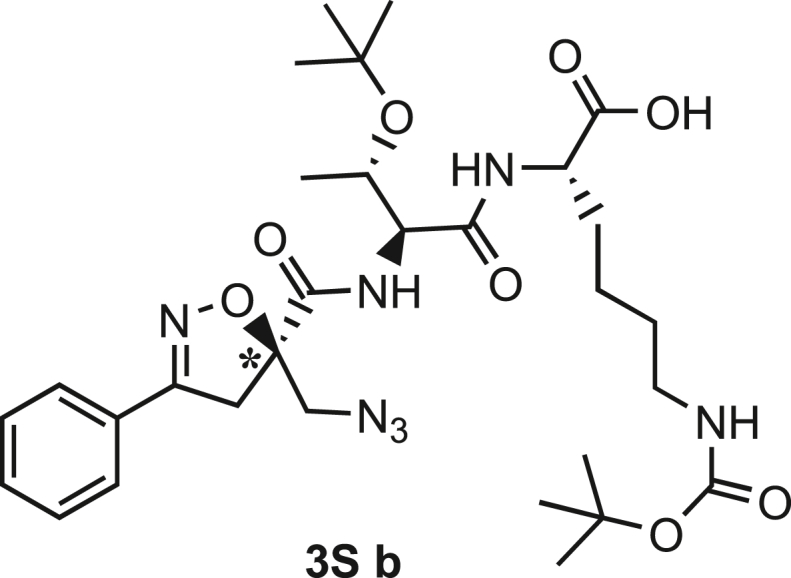

**Molecular weight**: 631.34 g/mol.

**Rf** (Hex/Et 7/3) = 0.

**MS**: Calcd. for [C_30_H_46_N_7_O_8_ + H]^+^: m/z 632.3402, found: 632.01; Calcd. for [C_30_H_46_N_7_O_8_ + Na]^+^: m/z 654.3222 found: 654.38

^**1**^**H NMR (400 MHz, CDCl**_**3**_**)**: δ 7.92 (1H, d, *J* = 6.5 Hz), 7.74–7.60 (3H, m), 7.45 (3H, m), 6.00 (1H, bs), 4.74–4.46 (1H, m), 4.37 (1H, *J* = 6.5Hz and 3.7 Hz, 1H), 4.18 (1H, m), 3.92 (1H, d, *J* = 13.1 Hz), 3.77–3.64 (2H, m), 3.56 (1H, d, *J* = 17.5 Hz), 3.14 (2H, m), 2.11–1.71 (2H, m), 1.46 (13H, bs), 1.28 (9H, s), 1.03 (3H, d, *J* = 6.3 Hz).

^**13**^**C NMR (101 MHz, CDCl**_**3**_**)**: δ 174.51, 170.93 , 169.26, 157.30, 130.91, 128.91, 128.29, 126.97, 89.15, 79.55, 75.47, 66.05, 57.81, 54.72, 52.44, 41.95, 40.29, 31.50, 29.70, 28.43, 28.24, 22.57, 17.66.

**Compound 4S b:** N^2^-(N-((R)5-(aminomethyl)-3-phenyl-4,5-dihydroisoxazole-5-carbonyl)-O-(tert-butyl)-L-allothreonyl)-N^6^-(tert-butoxycarbonyl)-L-lysine.

**Figure:**
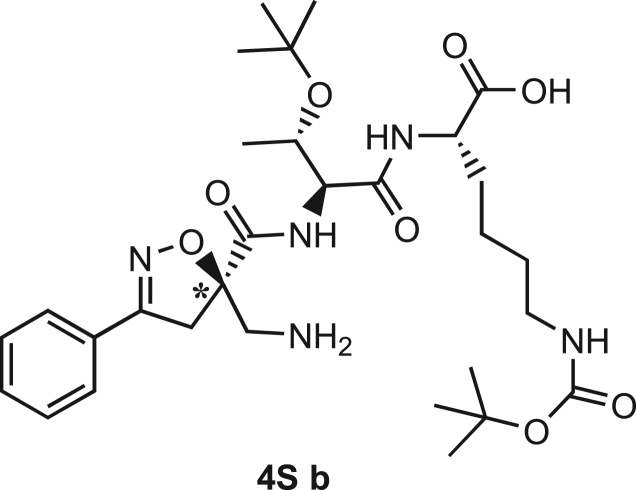

**4S b** was obtained as a pale yellow oil following the general procedure **D** starting from **3S b** (193 mg, 0.56 mmol, 1 eq.), H_2_O (49 eq.) and PMe_3_ (7 eq.). The crude obtained was directly used in the next step. The compound was used in the next step without any further purification. A small amount of crude was purified to fulfill the NMR characterization.

**Molecular weight**: 605.35 g/mol.

**R*f*** (DCM/MeOH 9/1) = 0.1.

**MS**: Calcd. for [C_30_H_47_N_5_O_8_ + Na]^+^: m/z 606.3497; found: 606.52 [M + Na]^+^

^**1**^**H NMR (400 MHz, CD**_**3**_**OD)**: δ 7.85–7.63 (2H, m), 7.58–7.34 (3H, m), 4.35–4.21 (3H, m), 3.81 (1H, d, *J* = 17.7 Hz), 3.67 (1H, d, *J* = 17.8 Hz), 3.55 (1H, d, *J* = 13.5 Hz), 3.33 (1H, m), 3.04 (2H, m), 1.92–1.67 (2H, m), 1.54–1.35 (13H, m), 1.21 (9H, s), 1.13 (3H, d, *J* = 6.3 Hz.

^**13**^**C NMR (101 MHz, CD**_**3**_**OD)**: δ 177.19, 171.98, 169.78, 158.15, 130.70, 128.61, 128.21, 126.70, 88.09, 80.78, 74.41, 66.69, 59.37, 54.75, 44.83, 42.54, 39.97, 32.22, 29.36, 27.45, 27.42, 22.59, 19.53.

**Compound 5S b:** *N* ^*2*^ *-(N-((R)-5-(((((9H-fluoren-9-yl)methoxy)carbonyl)amino)methyl)-3-phenyl-4,5-dihydroisoxazole-5-carbonyl)-O-(tert-butyl)-L-allothreonyl)-N* ^*6*^ *-(tert-butoxycarbonyl)-L-lysine.*

**Figure:**
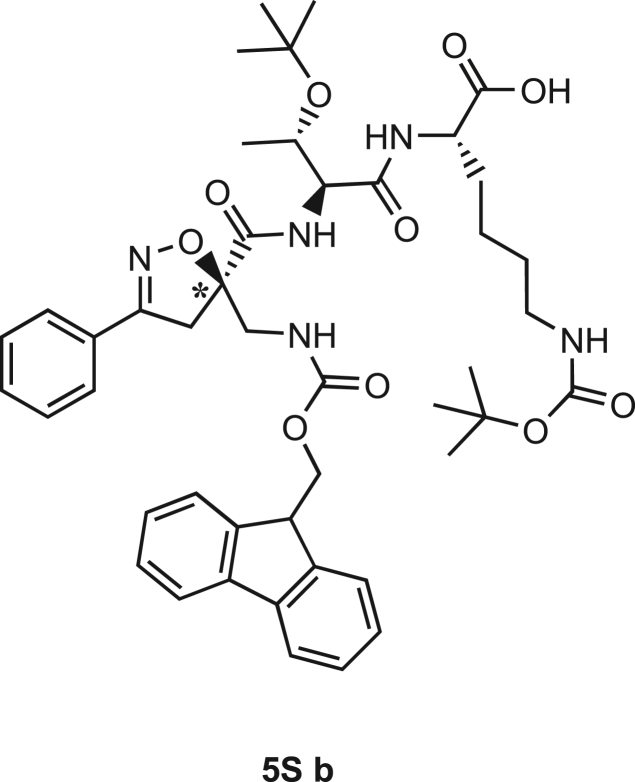

**5S b** was obtained following the general procedure **E** from **4S b** (0.55 mmol). TLC (DCM/MeOH + AcOH 95/5 + 1%) was used to monitor the reaction. The crude obtained was purified by chromatography on silica gel using DCM/MeOH + AcOH 97/3 + 1% as eluent to yield **5S b** (305 mg, 0.3685 mmol) as a white spongy solid in 67% yield.

**Molecular weight**: 827.42 g/mol.

**R*f*** (DCM/MeOH + AcOH 97/3 + 1%) = 0.5.

**[α]**_**20**_^**D**^**(3.20b)**: +25.1° ([c] = 0.01 in CH_2_Cl_2_)

**MS**: Calcd. for [C_45_H_57_N_5_O_10_ + Na]^+^: m/z 850.3998 found: 850.66 [M + Na]^+^

^**1**^**H NMR (400 MHz, CD**_**3**_**OD)**: δ 7.76 (2H, dd, *J* = 7.7 Hz and 3.6 Hz), 7.73–7.66 (2H, m), 7.62 (2H, d, *J* = 7.5 Hz), 7.45 (3H, m), 7.34 (2H, t, *J* = 7.5 Hz), 7.29–7.14 (m, 2H), 4.44 (1H, m, 1H), 4.37 – 4.25 (3H, m), 4.23 – 4.10 (2H, m), 3.84 (1H, d, *J =* 15.3 Hz), 3.77–3.63 (2H, m), 3.57 (1H, d, *J* = 17.7 Hz), 3.02 (2H, dd, *J* = 6.7 Hz), 1.97–1.81 (1H, m), 1.81–1.69 (1H, m), 1.43 (bs, 12H), 1.31 (1H, m), 1.21 (9H, s), 1.11 (3H, d, *J* = 6.2 Hz).

^**13**^**C NMR (101 MHz, CD**_**3**_**OD)** δ 172.27, 170.11, 157.89, 157.80, 157.09, 143.88, 141.12, 130.48, 128.56, 128.55, 127.35, 126.77, 126.63, 124.90, 119.46, 89.47, 79.60, 74.57, 66.91, 66.74, 58.35, 51.55, 44.70, 41.09, 39.77, 31.34, 29.17, 27.41, 27.38, 22.56, 18.53.

#### X-ray diffraction analysis of intermediate 11b

CCDC 2363965 contains the supplementary crystallographic data for this paper. The data is available from the Cambridge Crystallographic Data Centre via www.ccdc.cam.ac.uk/structures. An X-ray quality colourless needle (Figure S17, supplemental information) was obtained from the crystallization of the freshly synthesized compound from diethyl ether:hexane 1:1, overnight, with the slow evaporation method. The specimen is weakly pleochroic when seen through polarized light.

The data was collected at room temperature with a Bruker AXS Smart APEX 3-circle diffractometer equipped with a normal focus sealed X-ray Mo tube (λ=0.71073 Å) operating at a nominal power of 50 kV · 30 mA. A graphite monochromator was used in conjunction with a CCD detector. A 99.4 % complete sphere of diffraction data was recorded up to sinθ/λ = 0.56 Å^–1^, resulting in 4871 independent reflections, of which 3151 were significantly above the background (I > 2σ(I)). Integration of the diffraction effects was carried out with Bruker SAINT+. The raw data were corrected for absorption and possible anisotropies of the primary beam with SADABS [Bruker (2001). SADABS. Bruker AXS Inc., Madison, Wisconsin, USA]. The structure was solved by direct methods and subsequently refined with the SHELX suite of programs. ^59^ The least squares algorithm consisted of 451 parameters and 3 geometrical restraints. It converged to R1(F) = 0.1561 for 3151 F_o_ > 4σ(F_o_), with maximum and minimum Fourier residuals as large as Δρ = +1.45 / –0.33 e/Å^–3^ (see below).

The compound crystallizes in the triclinic chiral P1 space group with 2 formula units in the asymmetric unit, corresponding to the whole cell (Z = Z’ = 2). The unit cell parameters (Å, deg) are *a* = 5.9736(12), *b* = 10.901(2), *c* = 13.209(3), α = 93.05(3), β = 93.22(3), γ = 90.24(3), as estimated from 979 intense reflections among 6.2 and 58.8 deg in 2θ (final integration result). The cell volume reads V = 857.6(3) Å^3^, corresponding to estimated density ρ = 1.334 g·cm^−3^.

Figure S18 (supplemental information) shows the asymmetric unit of the title compound, while Figure S3 summarizes the main features of crystal packing. The compound is chiral; both molecules in the asymmetric unit share the same absolute configuration, that is, R, S at the stereogenic centers C9 (quaternary carbon), C12 (tertiary carbon). See Figure S18 for the atom numbering. The correctness of these attribution is secured by the value of the Flack parameter ^60^ computed on the final model, -0.8(9).

The 5-membred isoxazole–like rings share the same distorted envelope conformation, with puckering amplitudes and phases ^61^ 0.2116 Å, 135.19 (molecule 1, from C1 forward), or 0.2204 Å, 134.85 deg (molecule 2, from C201 forward). The C9 and C209 atoms lie 0.32(1)-0.33(1) Å apart from the mean least–squares plane computed from the atomic coordinates of the remaining atoms in the cycle (O1, N1, C7 and C8 or O201, N201, C207 and C208; see Figure S18 in supplemental information for the atom numbering).

Figure S19 (supplemental information) shows the main packing motifs in the (b,c), (a,c) and (a,b) planes. The title compound has at least one strong hydrogen bond donor (the acidic function), which in fact is employed to form zig-zag chains that run parallel to the a cell axis (Figure S19, Table S4, supplemental information). In turn, these involve alternately both the molecules in the asymmetric unit. Neither relevant intramolecular hydrogen bonds nor intermolecular stacking interactions were detected. The azide groups are aligned roughly along a, while the aromatic rings form apolar layers sandwiched between continuous –N_3_ and –COOH groups (Figure S20, supplemental information).

As a general remark, the specimen employed for the present analysis had a low scattering power and was slightly twinned. The minor twin component (<1 % *w/w*) affected only low-angle reflections and was ignored in the structure refinement procedure. The title compound crystallizes as very thin and long needles, which tend to exfoliate into smaller pieces even under moderate mechanical stress. Despite extensive crystallization attempts, no better specimens were found. However, the resolution of the present dataset is high enough to unequivocally solve the structure, which was the main aim of the X-ray experiment. The current structural model does not account for a couple of weak positive residual Fourier peaks, roughly 1.3-1.4 e·Å^-3^ large, that lie close to the azide groups of both the molecules in the asymmetric unit. More in detail, these residues are placed at roughly 1.6-1.8 Å from nitrogens N2 and N202 (Figure S18, supplemental information), along the main azide axis. They could be related to co-crystallized water molecules, but we prefer to not explicitly attribute them as higher-quality specimens should be required to gain insights on their nature.

#### Circular dichroism

Compounds, previously pre-treated with HFIP, were dissolved in MQ water to a concentration of 500 μM as stock solutions. Before measurement, each compound was diluted to 125 μM concentration with 10 mM PB (pH 7.2 or pH 5.1) buffer or H2O into a cuvette with a path length of 1 mm. The CD spectra were recorded by J-815 spectropolarimeter (JASCO, Tokyo, Japan) from 190 to 260 nm at 20 and 37°C and a scan rate of 50 nm/min (accumulation n=3). Each CD spectrum was corrected by subtracting the corresponding baseline (PB buffer 10 mM).

#### FTIR spectroscopy

Infrared spectra were recorded using a Shimadzu IRAffinity-1S spectrometer in the 600–4000 cm–1 range with a resolution of 2 cm^–1^. A sample for measurements in the solid state was prepared by dissolving the peptidomimetic in PB 10 mM (previously treated with HFIP), placing the solution on the crystal plate, and evaporating the solvent (64 scans were averaged). The ATR FTIR experiments were measured from a solution at 125 μM compound concentration. Transmittance has been recorded and transformed into absorbance [A = 2–log(T)]. Data processing was performed using solver in excel software (Microsoft). Deconvolution of the spectra was done in the spectral range of 1500–1800 cm^–1^. The deconvoluted spectra were fitted with Gaussian band profiles. The positions and number of the components used as an input file for the curve-fitting function were obtained from the deconvoluted spectra. The quality of the fitting was estimated by the standard deviation and error squared.

#### Self-aggregation assay by ThT fluorescence spectroscopy

Ac-PHF6∗-NH_2_ and compounds **1** and **2** were dissolved in pure hexafluoro-isopropanol (HFIP) at a concentration of 1 mM and incubated for 10 minutes at room temperature to dissolve any preformed aggregates. Next, HFIP was evaporated under a stream of dry nitrogen gas followed by vacuum desiccation for at least 3 hours. The resulting thin film was then dissolved in 20 mM MOPS buffer (pH 7.4), sonicated for 1 min, and vortexed for 2 min to get a fully dispersed clean solution at 500 μM concentration. Thioflavin-T binding assays were used to measure the formation of fibrils in solution using a plate reader (Fluostar Optima, BmgLabtech) and standard 96-wells flat-bottom black microtiter plates (final volume 200 μL, 440 nm of excitation wavelength and 480 nm for emission). ThT assay was started by adding 2 μL of a 100 μM heparin solution (heparin sodium salt H-3149, average MW 18 kDa, final concentration 1 μM) to a mixture containing 25 μM of Ac-PHF6∗-NH_2_ or compounds **1** and **2**, 20 μM ThT in 20 mM MOPS pH 7.4 buffer. Fluorescence data were normalized by putting the max fluorescence of Ac-PHF6∗ at 100% (time of kinetics 60 min). The results are represented as the average of three replicates (n = 3) and the error bars are indicated as the ±SEM.

#### Purification of recombinant Wt-Tau

For the TEM analysis, native Tau was purified from the cell extract by sequential anion exchange and phosphocellulose chromatography as previously described. The Tau protein eluate was dialyzed against water and concentrated with Vivaspin® (15R, 2,000 MWCO, Sartorius, UK). Protein concentrations were determined by densitometry of Coomassie Brilliant Blue-stained gels using bovine serum albumin as a standard.^48^ For ThT assays, Tau441wt protein was also purified using heat denaturation following established protocols^56^ and dialyzed against 50 mM ammonium bicarbonate buffer before being lyophilized.

#### ThT fluorescence spectroscopy on wt-Tau441

Lyophilized full-length wt-Tau441 was diluted to 40 μM in PB 25 mM, NaCl 25 mM, and EDTA 2.5 mM pH 6.8. Stock solutions of compounds **1**, **2** and **3** were prepared in water as described in the general protocol « Self-aggregation assay by ThT fluorescence spectroscopy ». ThT fluorescence was measured to evaluate the development of Tau fibrils over time using a fluorescence plate reader (Fluostar Optima, BMG labtech) with 384-wells flat-bottom black plates (final volume in the wells of 40 μL). Experiments were conducted with and without the addition of heparin (final concentration 0.1 μM) and with or without compounds **1** and **2** (final concentration 1 μM) and with different concentrations of compound **3** (50 μM, 10 μM, and 1 μM). The final concentration of tau441 was maintained at 10 μM and that of Thioflavin-T at 25 μM. The ThT fluorescence intensity of each sample (performed in triplicate) was recorded every 10 min (440/480 nm excitation/emission) during 140 h or 40 h under continuous agitation (orbital shaking) at 37 °C on plates sealed with a transparent film. The kinetic curves represent the average of measurements made in triplicate from two different experiments and the error bars are indicated as the ±SEM.

#### Transmission electron microscopy (TEM)

Wt-Tau441 was dissolved in PB buffer (Na2HPO4 and NaH2PO4 25 mM, NaCl 25 mM, EDTA 2.5 mM, pH 6.6) to a final concentration of 40 μM (stock solution). Sample preparation: Experiments were started by adding 10 μL of buffer, 10 μL of Tau solution (final concentration of 10 μM), 10 μL of compound 1, 2 or 3 (either 200 μM or 40 μM or 4 μM to have 5:1, 1:1 and 0.1:1 ratios, respectively), and, when necessary, 10 μL heparin solution solubilized in NaPi buffer (final concentration 0.1 μM). In blank analysis, 10 μL of active compound was replaced by 10 μL MQ H2O and 10 μL heparin solution by 10 μL of buffer. These solutions were maintained at 37°C for 96 h at 1400 rpm.

The samples were prepared on carbon-coated copper grids (ECF200- Cu, 200 mesh, Science Services, Munich, Germany). The grids were pre-treated with argon in a plasma cleaner (Diener Electronics, Ebhausen, Germany). The sample (1.2 μL) was applied onto the grids, and after a sedimentation time of 5 minutes, the excess suspension was removed with filter paper. Grids were then stained with 1% acetate uranyl solution (1.2 μL) for 5 minutes, excess stain was removed with filter paper and washed thoroughly with 3 x 10 μL of Milli-Q water. The prepared grids were analyzed with a JEOL JEM-2200FS electron microscope (JEOL, Freising, Germany), a cold field emission electron gun, and an applied acceleration voltage of 200 kV. A bottom-mounted Gatan OneView camera (Gatan, Pleasanton, CA, USA) was used for digital recording. The images were processed using the image-processing system Digital Micrograph GMS3 (Gatan, Pleasanton, CA, USA) and the image editing software ImageJ. To investigate the patterns, the images were analyzed, selected, and edited to show similarities and differences using the software ImageJ. Width distribution analyses of at least 100 individual fibrils from different fields of view in the same specimen were performed.

#### Metabolic activity and cytotoxicity profiling using a combined LDH and MTT Assays

PC12 cells were cultured in 96-well plates at 1×104 cells/well in 50 μl of serum-reduced medium supplemented with 100 ng/ml 7S mouse NGF to induce neuronal differentiation. Cells were incubated for 48, test compounds were added in an additional volume of 50 μl, and incubation was continued for 20 hours. For the LDH assay, 50 μl medium from each well of the assay plate was transferred to a separate 96-well plate. To quantify LDH release, 50μl of LDH reagent (4 mM iodonitrotetrazolium chloride (INT), 6.4 mM beta-nicotinamide adenine dinucleotide sodium salt (NAD), 320 mM lithium lactate, 150 mM of 1-methoxyphenazine methosulfate (MPMS) in 0.2 M Tris-HCl buffer, pH 8.2) was added. Absorbance was measured at 490 nm using a Thermomax Microplate Reader operated with SoftMaxPro Version 1.1 (Molecular Devices Corp., Sunnyvale, CA, U.S.A.). For the MTT assay, MTT reagent (3,(4,5.dimethylthiazol-2-yl)2,5-diphenyltetrazolium bromide) was added to the wells of the remaining assay plate at a final concentration of 1 mg/ml MTT. Cells were incubated for 2 hours before the reaction was stopped by adding 50 μl of lysis buffer (20% (wt/vol) sodium dodecyl sulfate in 1:1 (vol/vol) N,N-dimethylformamide/water, pH 4.7). After overnight incubation at 37 °C, optical densities of the formazan product were determined at 570 nm. MTT conversion measurements were normalized to the optical densities of negative control wells. All experiments were performed in triplicates on two independent plates.

#### Live-cell imaging and Fluorescence Decay After Photoactivation (FDAP)

FDAP experiments were performed essentially as previously described.^1^ Briefly, cells expressing TauΔK280 were plated on 35-mm glass-bottom culture dishes (MatTek, USA), transfected, and neuronally differentiated by medium exchange to serum-reduced DMEM containing 100 ng/mL 7S mouse NGF. After 3 days, the medium was exchanged to serum-reduced DMEM without phenol red with NGF, and the respective compound (or DMSO for carrier control) was added at the desired concentration. After 20 hours, live cell imaging was performed using a laser scanning microscope (Nikon Eclipse Ti2-E (Nikon, Japan)) equipped with a LU-N4 laser unit with 488-nm and 405-nm lasers and a Fluor 60× ultraviolet-corrected objective lens (NA 1.4) enclosed in an incubation chamber at 37°C and 5% CO2. Photoactivation was performed with a 405-nm laser using the microscope software (NIS-Elements version AR 5.02.03 (Nikon, Japan)). A series of consecutive images were acquired at a frequency of 1 frame/s, and 112 images were collected per activated cell at a resolution of 256×256 pixels. Effective diffusion constants were determined by fitting the fluorescence decay data from the photoactivation experiments using a one-dimensional diffusion model function for FDAP. A reaction-diffusion model was used to estimate the association rate k∗on and the dissociation rate k∗off constant of tau binding.

### Quantification and statistical analysis

Statistical analysis was carried out with GraphPad Prism v8.0.1 (GraphPad Software, USA). All data sets were tested for normality using D’Agostino-Pearson and Shapiro-Wilk test. If necessary, data sets were log transformed in order to enable further statistic testing. Statistical outliers were identified using the ROUT method. The homogeneity was assessed using Levene’s test. An unpaired two-tailed *t*-test was used to compare two datasets. In the case of unequal variances, the Welch’s correction was applied. A one-way ANOVA followed by Dunnett’s post-hoc test was performed to compare more than two data sets with a single control. All statistical values are expressed as mean ± SEM. All the information regarding the number of cells involved in the statistical analysis, the statistical test used and the stars representing *p* values can be found in the figures’ legend.
