# Supplementary material for: Application of modular isoxazoline-β2,2-amino acid-based peptidomimetics as chemical model systems for studying the tau misfolding
Source: iScience. 2025 Mar 22;28(4):112272. doi: 10.1016/j.isci.2025.112272 (PMC12013406; doi:10.1016/j.isci.2025.112272)
Supplement: Document S1. Figures S1–S20 and Tables S1–S4 [file mmc1.pdf]

iScience, Volume *28*

Supplemental information

Application of modular isoxazoline-$\beta^{2,2}$-amino acid-based peptidomimetics as chemical model systems for studying the tau misfolding

Davide di Lorenzo, Nicolo Bisi, Raffaella Bucci, Inga Ennen, Leonardo Lo Presti, Veronica Dodero, Roland Brandt, Sandrine Ongeri, Maria-Luisa Gelmi, and Nicolo Tonali

Figure S1: Circular dichroism of N_3-Isox-$\beta^{2,2}$-OH at 1 mM in water, related to Figure 2A

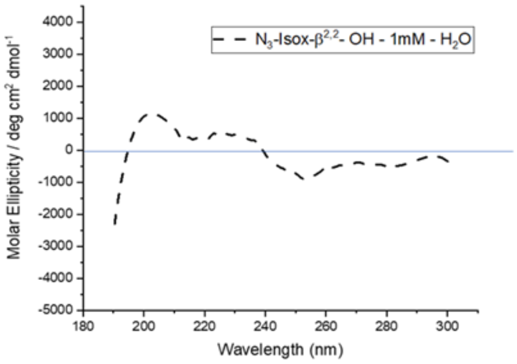

Figure S2: Comparison of CD spectra of 1 and 2 at time 0 and at 24h in 20 mM PB pH 7.2, related to Figure 2A

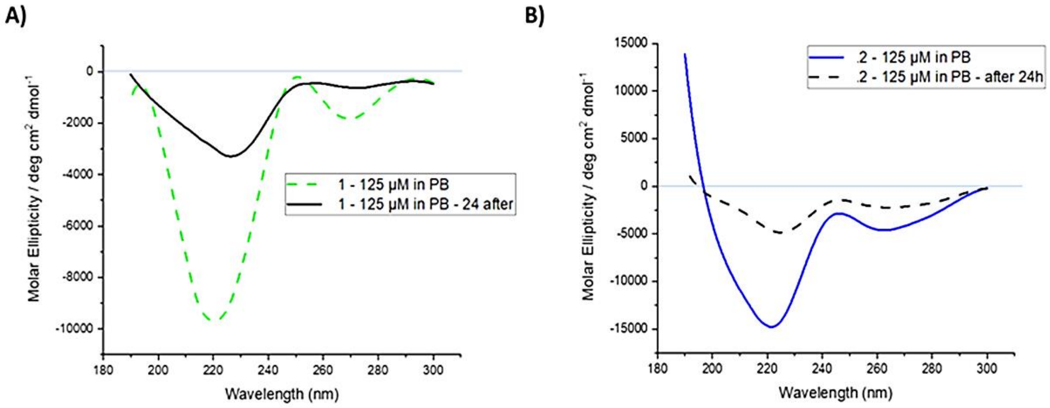

A) CD spectrum of **1** at 125 μM; B) CD spectrum of **2** at 125 μM.

Figure S3: CD spectra of compounds 1 and 2 in 20 mM PB (125 μM) at pH 5.1, related to Figure 3.

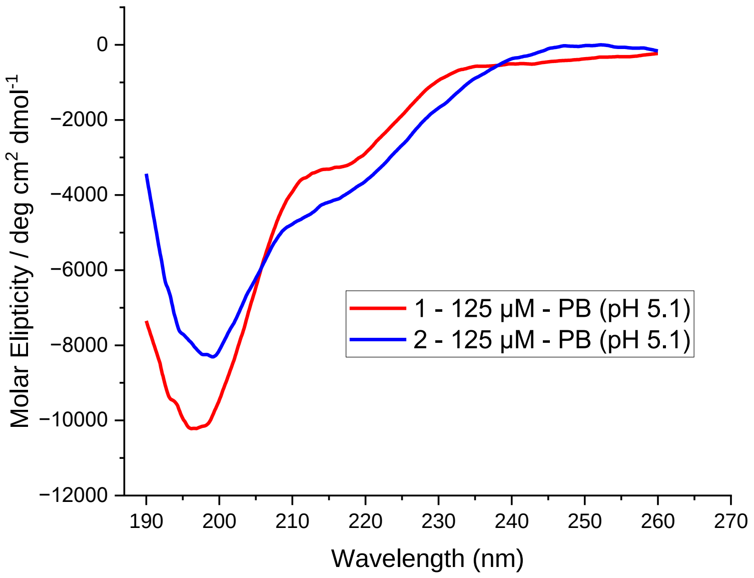

Figure S4: The 1H-1H ROESY spectrum showing sequential and inter-strand ROEs of compound 2, related to Figure 4B.

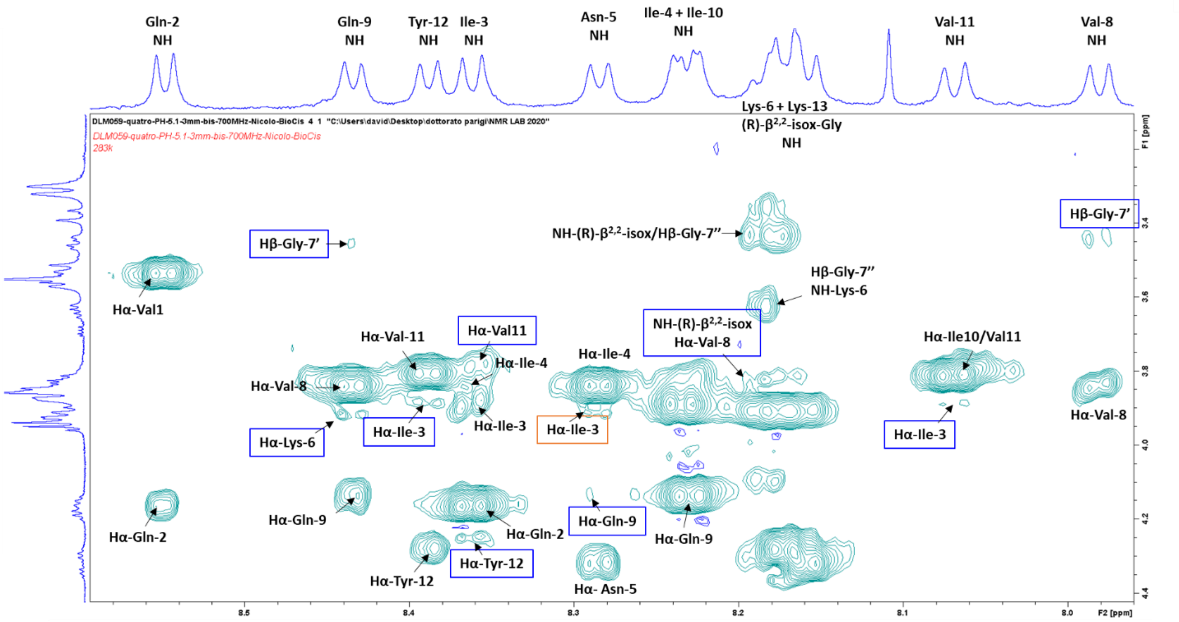

Sequential CH$_\alpha$/NH (i, $i+1$) ROEs are highlighted in black and inter-strand CH$_\alpha$/NH interactions highlighted in blue.

Figure S5: Transmission Electron Microscopy (TEM) images of control for compounds 1 and 2, related to Figure 6B.

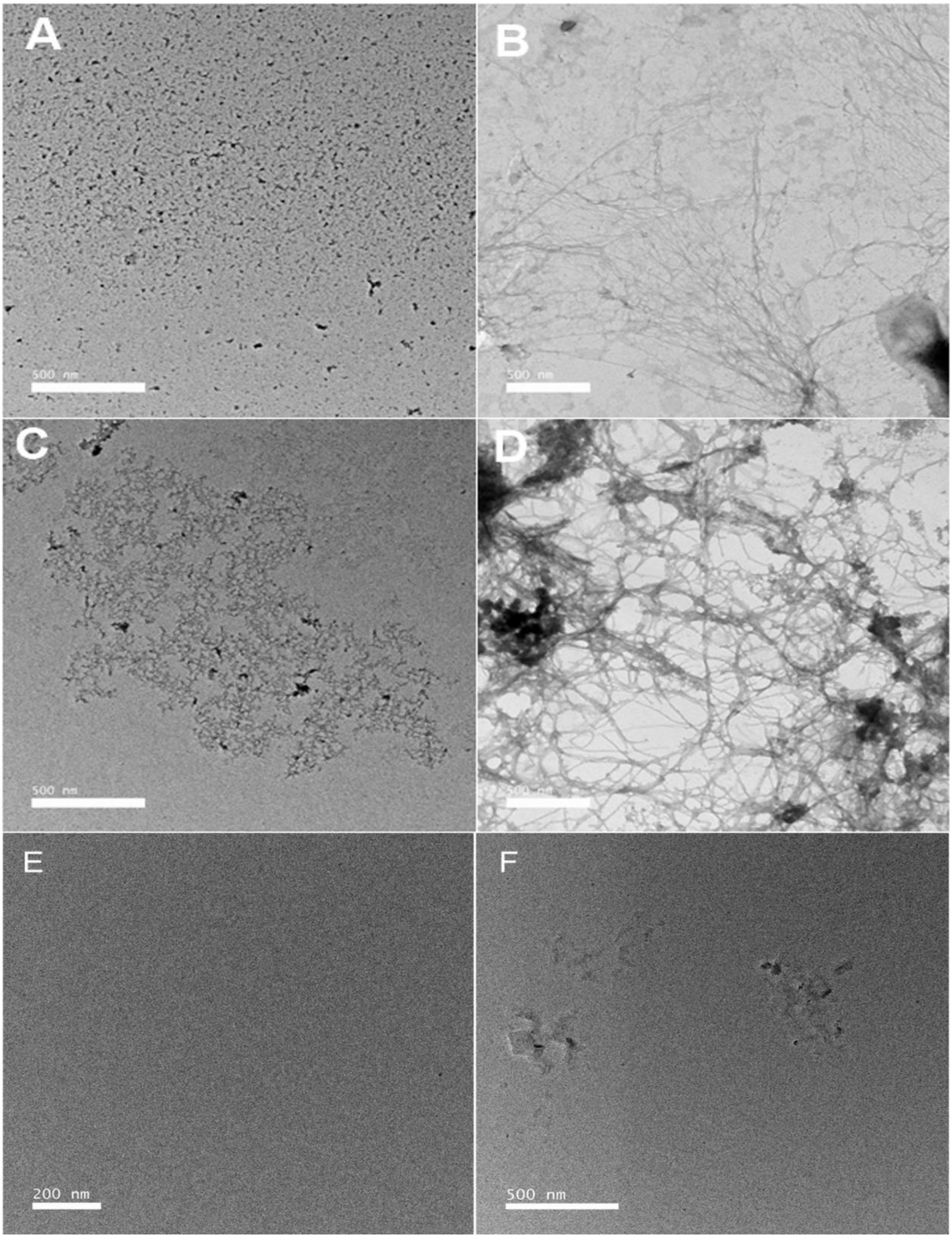

A) Tau (10 µM) without heparin; B) Tau (10 µM) with heparin (0.1 µM); C) Tau (10 µM) and **2** (1 µM); D) Tau (10 µM) and **1 (**1 µM); E) Compound **1** at 1 µM; F) Compound **2** at 1 µM.

Figure S6: Representative low-magnification pictures of tau fibrils, related to Figure 6B.

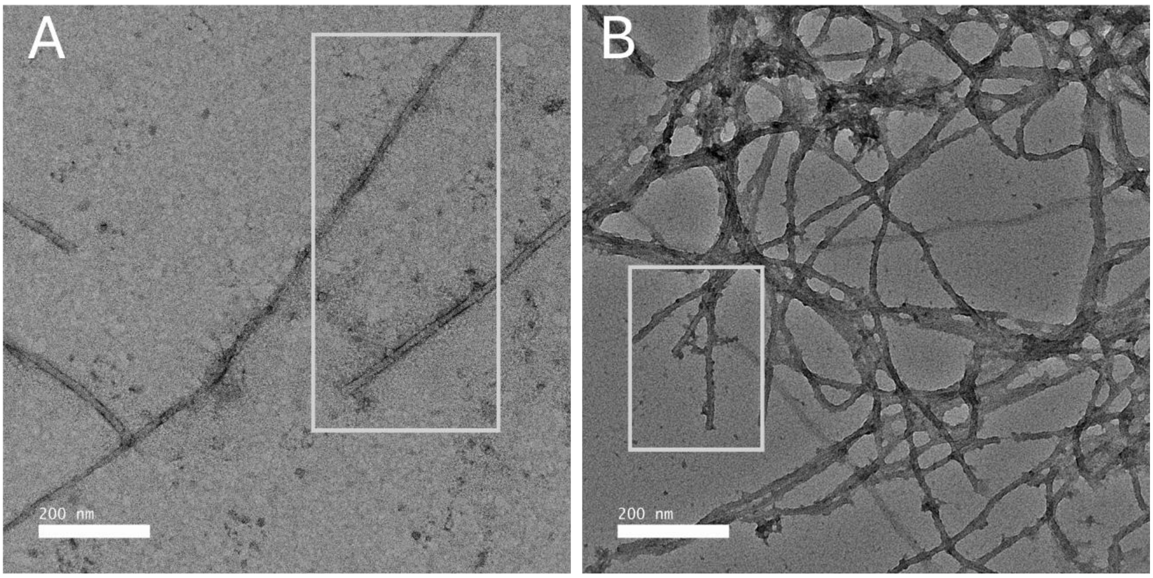

A) Tau fibrils in the presence of heparin (0.1 µM); B) Tau fibrils in the presence of compound **1** (1 µM).

Figure S7: MTT assay on compound 1 at different concentrations (from 3.12 to 50 µM), related to Figure 6.

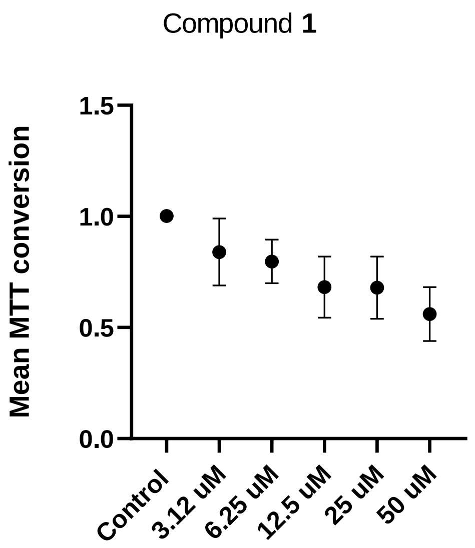

The graph represents two independent plates with three replicates per compound concentration. Error bars indicate mean values ± SEM. Each concentration: n = 6; Positive control: n = 8; Negative control: n = 10. As control, DMSO 0.6% is used.

Figure S8: FDAP analysis of 1 on PC-12 cells transfected with PAGFP-Tau441 ΔK280, related to Figure 6C.

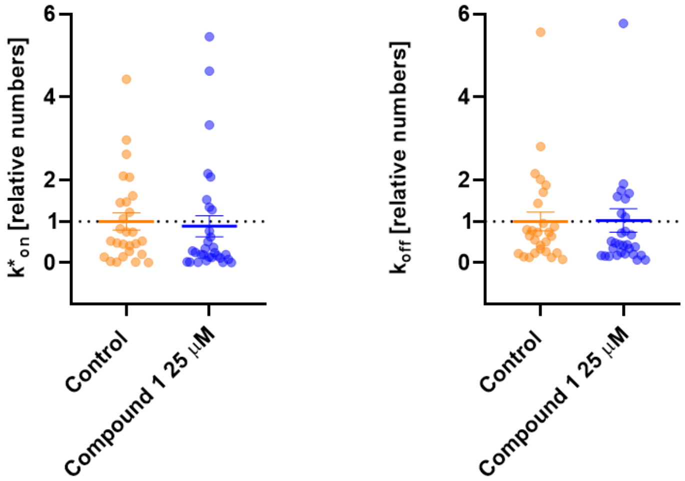

FDAP data were fitted with a mathematical model, whose outcome allowed the analysis of Tau K_{on} and K_{off}. Experiments were performed with differentiated model neurons (PC12) cells differentiated with NGF), pre-incubation time was 24 h, DMSO concentration 0.125% (Control).

Figure S9: FDAP decay images of PAGFP-ΔK280 transfected cells at 0, 2, 20 and 90 sec, related to Figure 6C.

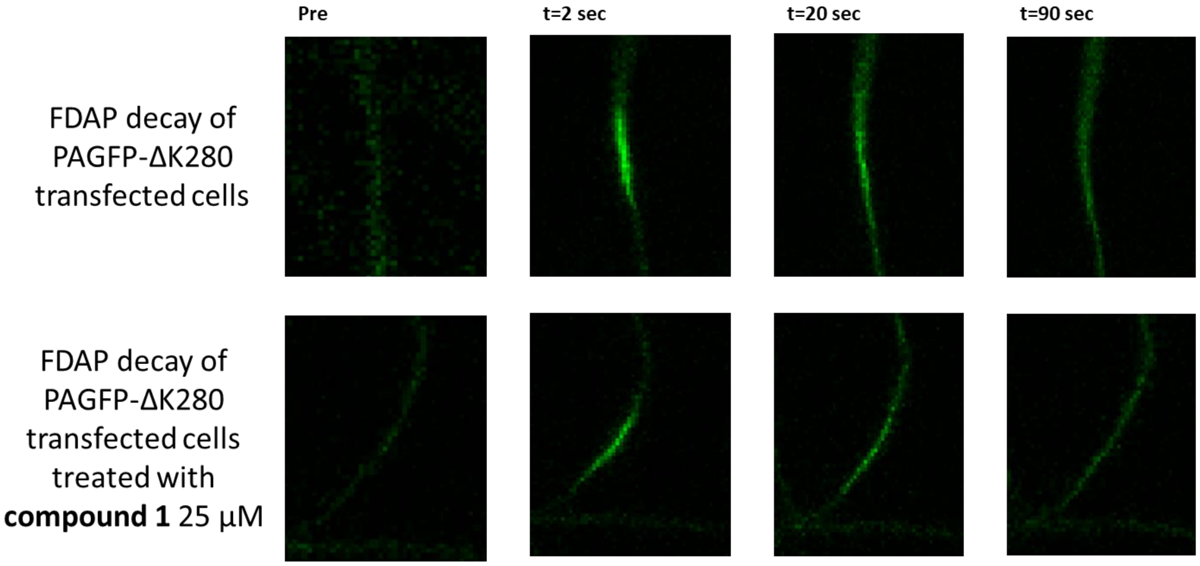

Control (up) and in the presence of compound **1** (down).

Figure S10: Circular dichroism and infra-red spectroscopy of compound 3, related to Figure 7.

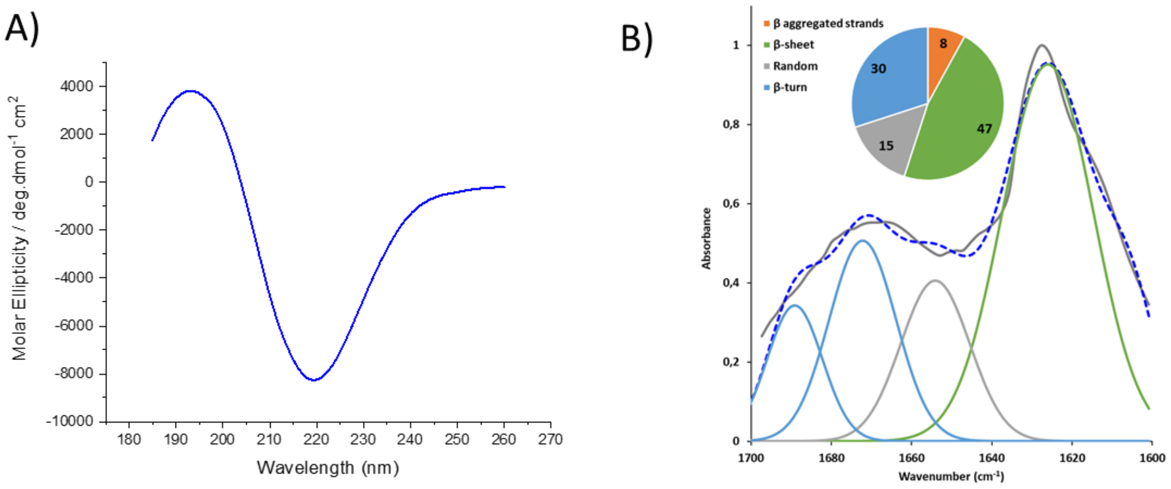

A) CD spectrum of compound **3** in 20 mM PB pH 7.2 at 125 µM concentration; B) IR-ATR amide I deconvolution of **3** (error squared = 0.068, standard deviation 7.74) in PB (125 µM, pH 7.2) with schematic representation of their secondary structure contents.

Figure S11: Transmission Electron Microscopy (TEM) images of control for compound 3, related to Figure 7B.

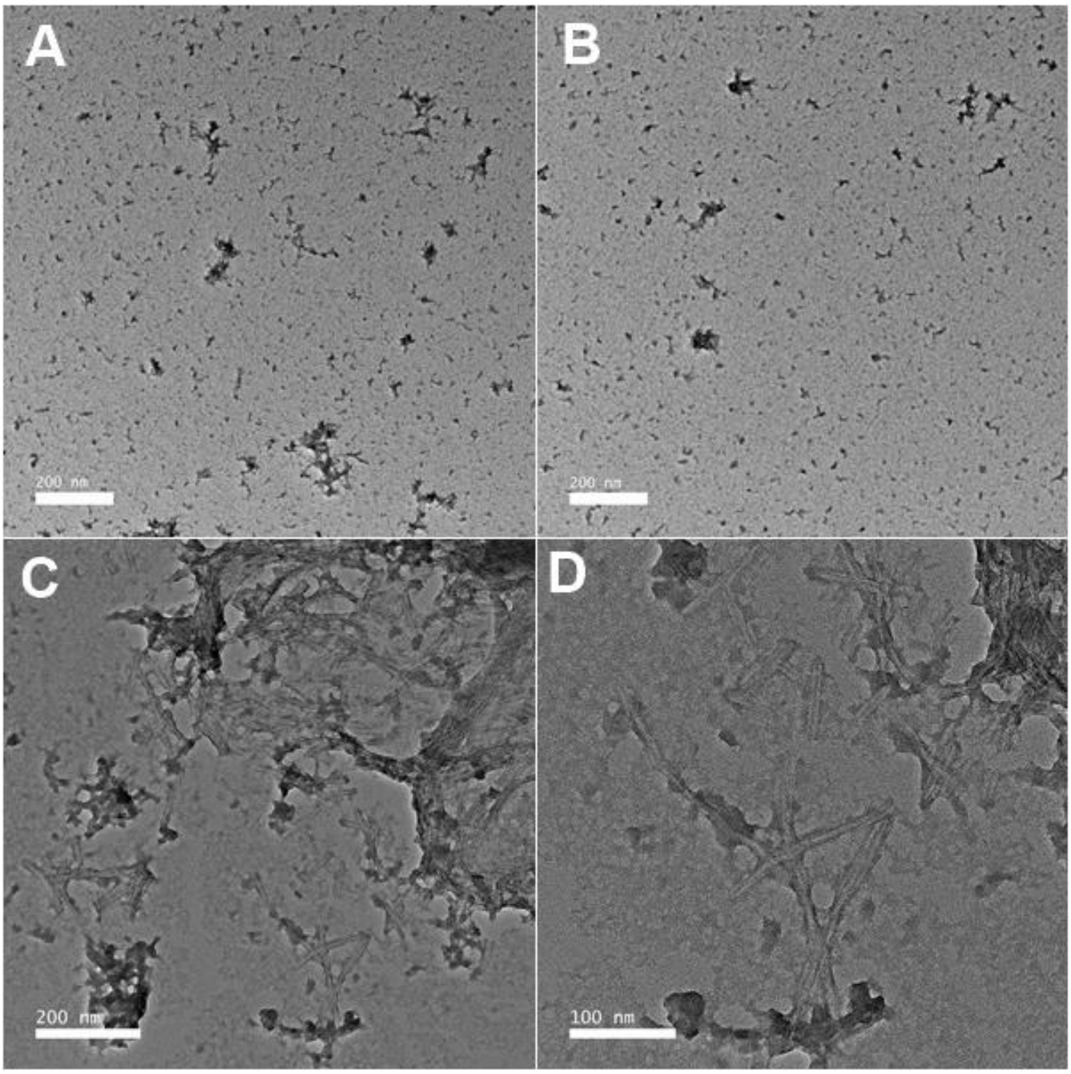

A) Tau (10 µM) and **3** (50 µM) B) Tau (10 µM) and **3** (10 µM) C-D) Tau (10 µM) and **3** (1 µM).

Figure S12: FDAP analysis of 3 on PC-12 cells transfected with PAGFP-Tau441 ΔK280, related to Figure 7C.

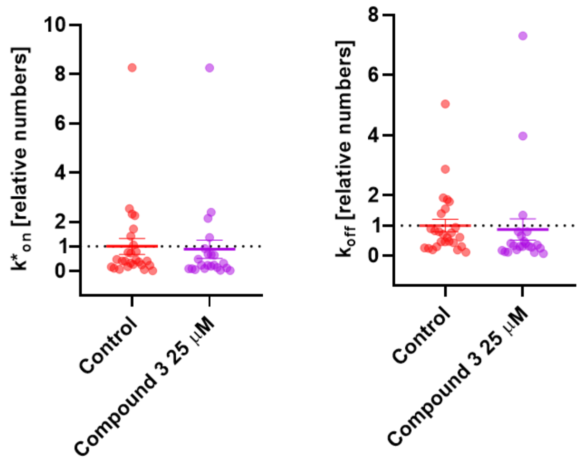

The fluorescence decay is fitted with a mathematical model, whose outcome allow to analyze Tau K_{on} and K_{off}. Experiments were performed with differentiated model neurons (PC12) cells differentiated with NGF), compound incubation time was 24 h, DMSO concentration 0.125% (Control).

Figure S13: FDAP decay images of PAGFP-ΔK280 transfected cells at 0, 2, 20 and 90 sec, related to Figure 7C.

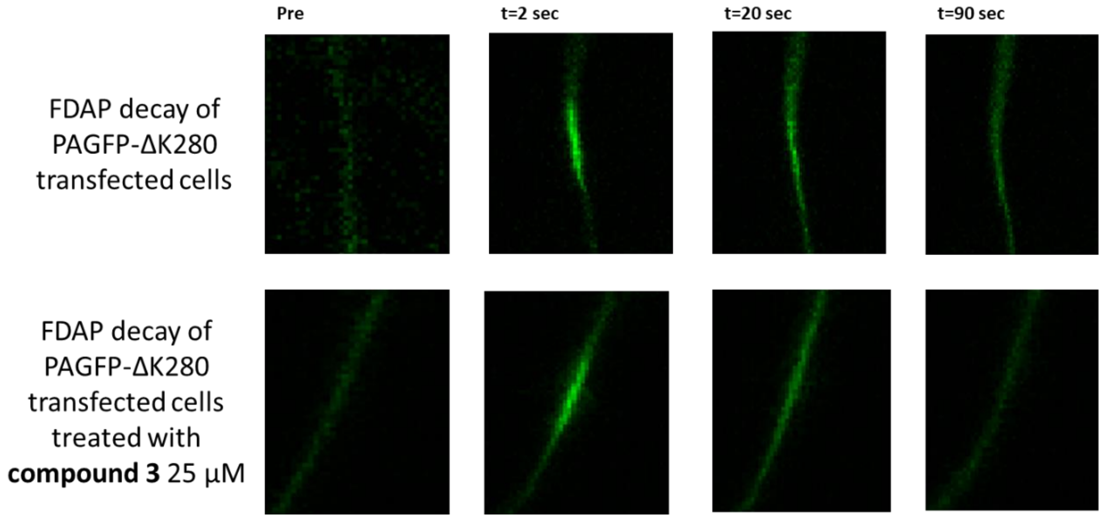

Control (up) and in the presence of compound **3** (down).

Figure S14: LC-MS and HRMS of compound 1

DLM-061	P1-C9	HP-gr-ACN-5-100.m	z-DLM-061-01.d	2	Davide DI LORENZO	FLUOR	XSELECT 01433023015 - 2.1x75mm 2.5µm - H2O/ACN/0.1%AF

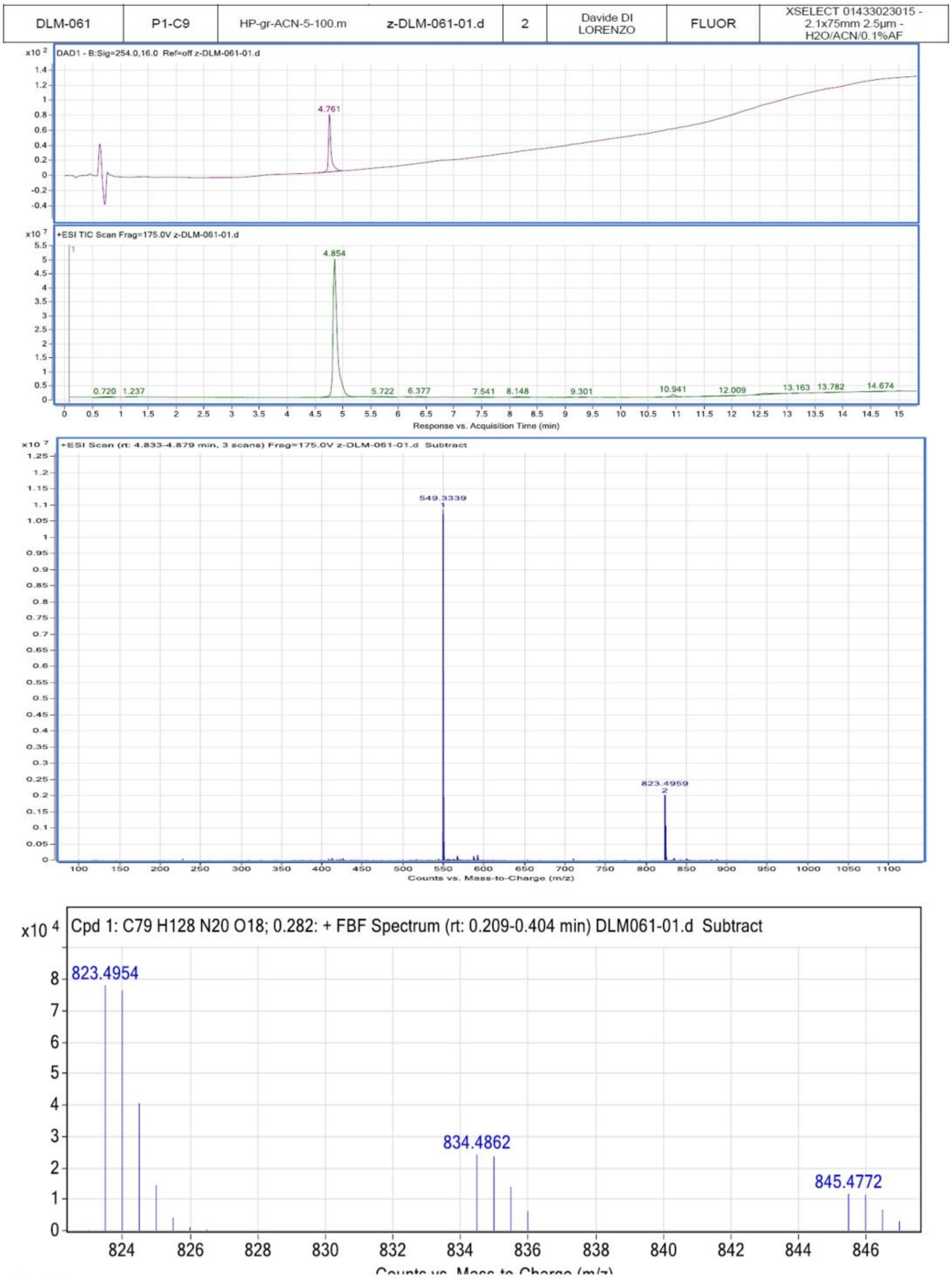

Peak List

m/z	z	Abundance	Target Formula	Target Ion Species	Calculated m/z	Diff (mDa)	Diff (ppm)
823,4954	2	78101,54	C79H128N20O18	(M+2H)+2	823,4931	2,38	**2,89**
823,9967	2	76445,04	C79H128N20O18	(M+2H)+2	823,9945	2,19	**2,66**
824,4980	2	40560,20	C79H128N20O18	(M+2H)+2	824,4959	2,07	**2,51**
834,4862	2	24212,14	C79H128N20O18	(M+H+Na)+2	834,4840	2,20	**2,64**
834,9871	2	23555,83	C79H128N20O18	(M+H+Na)+2	834,9855	1,62	**1,94**

Figure S15: LC-MS and HRMS of compound 2

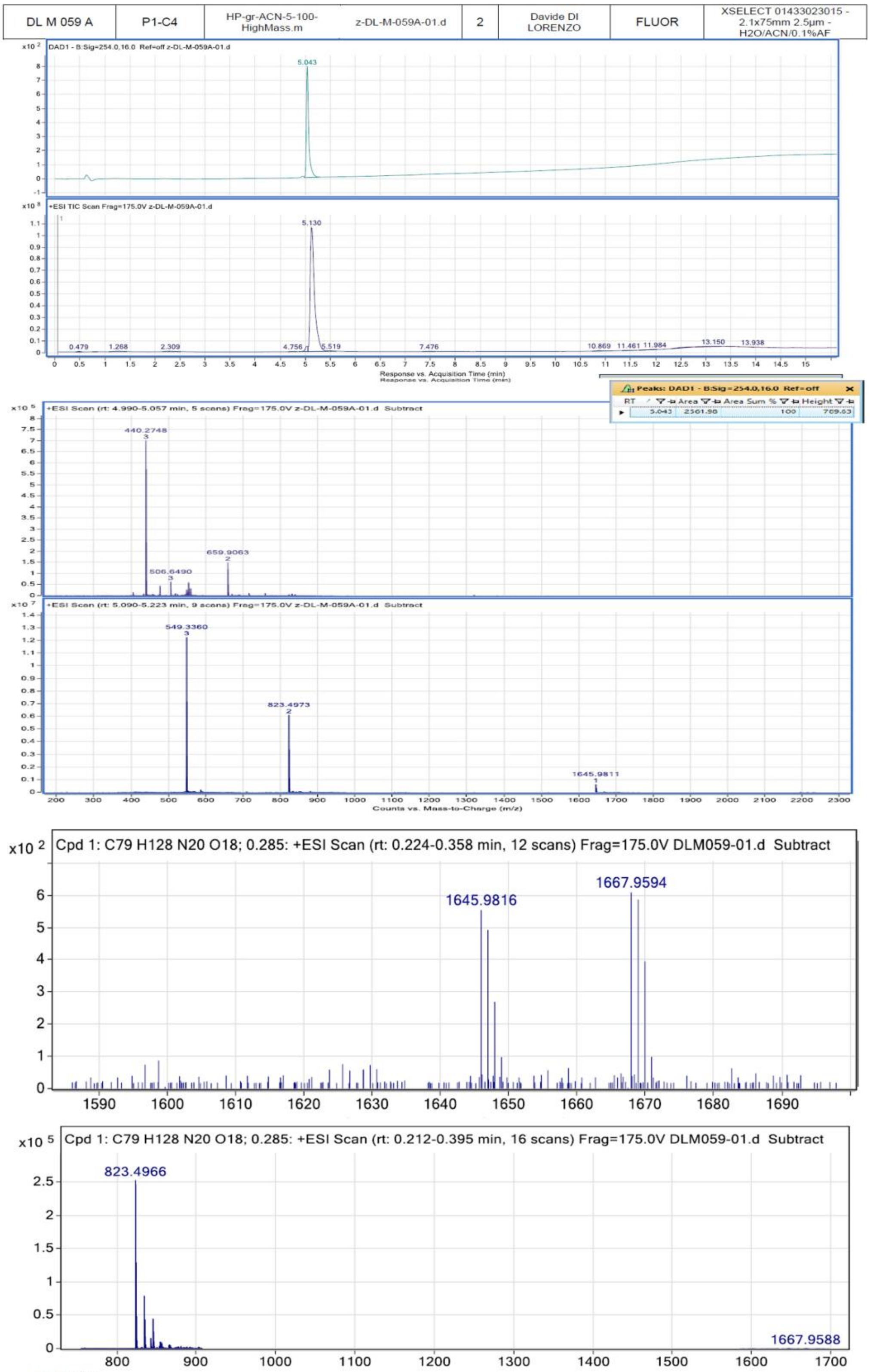

Peak List

m/z	z	Abundance	Target Formula	Target Ion Species	Calculated m/z	Diff (mDa)	Diff (ppm)
823,4966	2	252812,27	C79H128N20O18	(M+2H)+2	823,4931	3,58	**4,35**
823,9978	2	247020,44	C79H128N20O18	(M+2H)+2	823,9945	3,28	**3,99**

Figure S16: LC-MS and HRMS of compound 3

DLM 190-1	P1-C2	HP-gr-ACN-5-100-HighMass.m	z-DLM190-1-01.d	2	Davide DI LORENZO	FLUOR	XSELECT 01433023015 - 2.1x75mm 2.5µm - H2O/ACN/0.1%AF

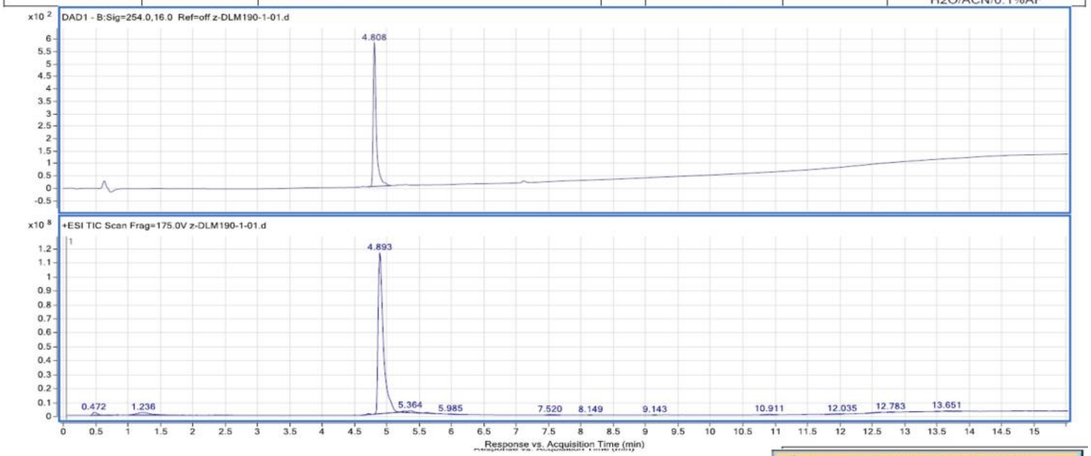

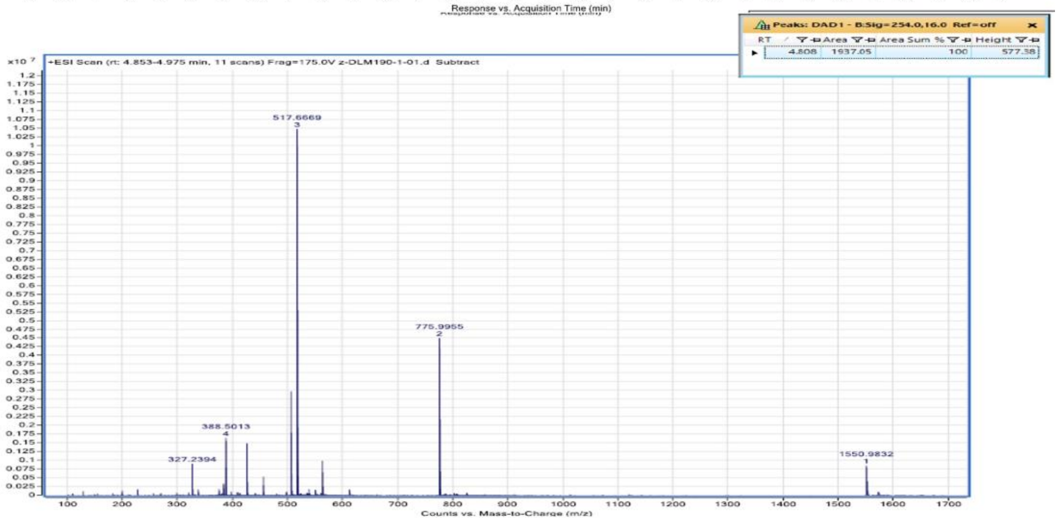

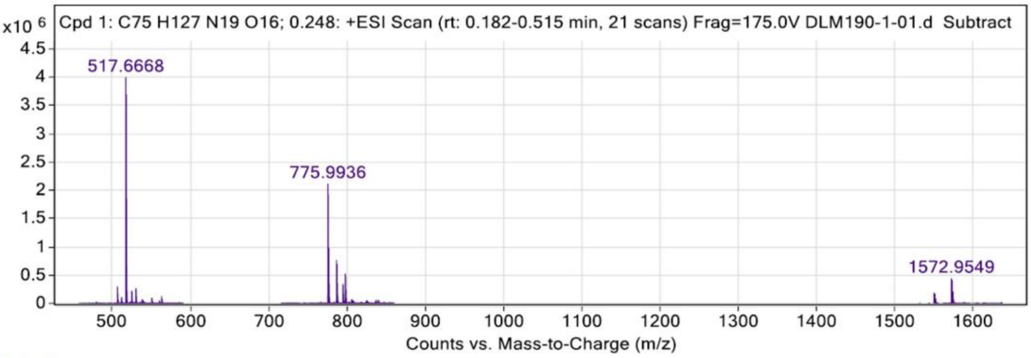

Peak List

m/z	z	Abundance	Target Formula	Target Ion Species	Calculated m/z	Diff (mDa)	Diff (ppm)
517,6668	3	3998935,25	C75H127N19O16	(M+3H)+3	517,6642	2,55	**4,93**
518,0010	3	3693351,50	C75H127N19O16	(M+3H)+3	517,9985	2,52	**4,86**
518,3351	3	1854781,75	C75H127N19O16	(M+3H)+3	518,3328	2,27	**4,38**
775,9936	2	2116123,00	C75H127N19O16	(M+2H)+2	775,9927	0,91	**1,17**
776,4950	2	1924265,13	C75H127N19O16	(M+2H)+2	776,4941	0,89	**1,14**
1550,9712	1	256764,48	C75H127N19O16	(M+H)+	1550,9781	-6,92	**-4,46**
1551,9743	1	233447,08	C75H127N19O16	(M+H)+	1551,9810	-6,69	**-4,31**
1572,9530	1	611022,88	C75H127N19O16	(M+Na)+	1572,9600	-7,08	**-4,50**
1573,9560	1	561111,56	C75H127N19O16	(M+Na)+	1573,9630	-7,00	**-4,45**

Figure S17: A single crystal of 11b

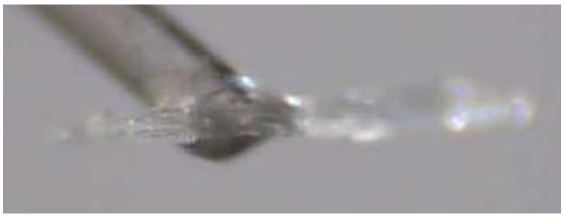

The crystal is mounted on the top of a capillary fibre with a drop of bicomponent epoxy glue. The dimensions of the crystal are ~ 0.925 x 0.075 x 0.075 mm.

Figure S18: Asymmetric unit of 11b

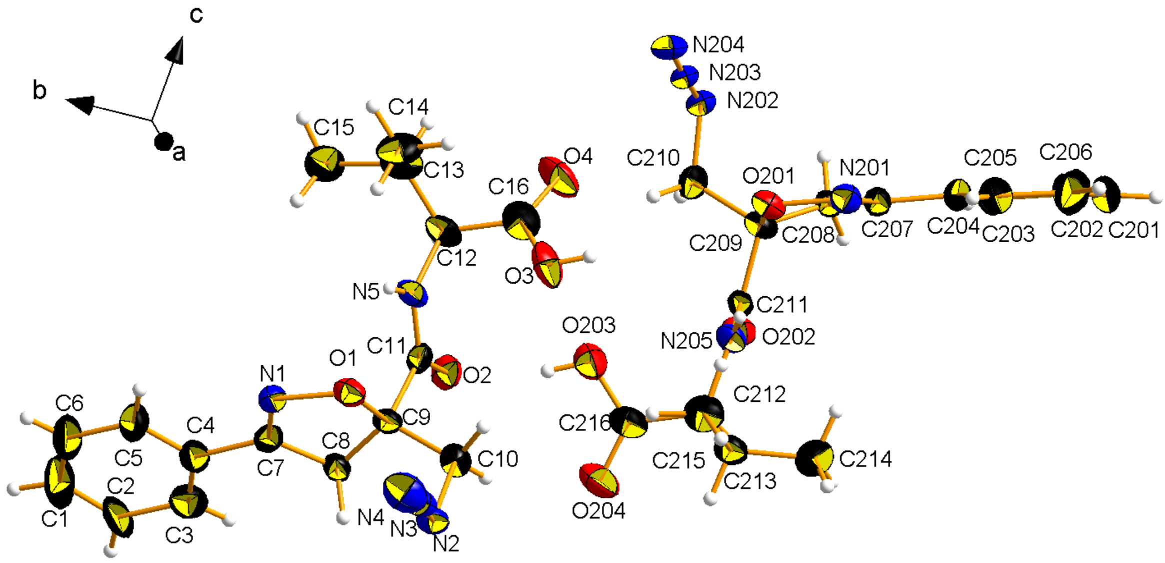

The atom-numbering scheme is highlighted for the symmetry–independent part of the molecule. Thermal ellipsoids at RT were drawn at the 30 % probability level. Atoms are represented with the usual colour code (C: black; N: blue; O: red; H: white).

Figure S19: Wires–stick representation of the crystal packing of 11b at room temperature

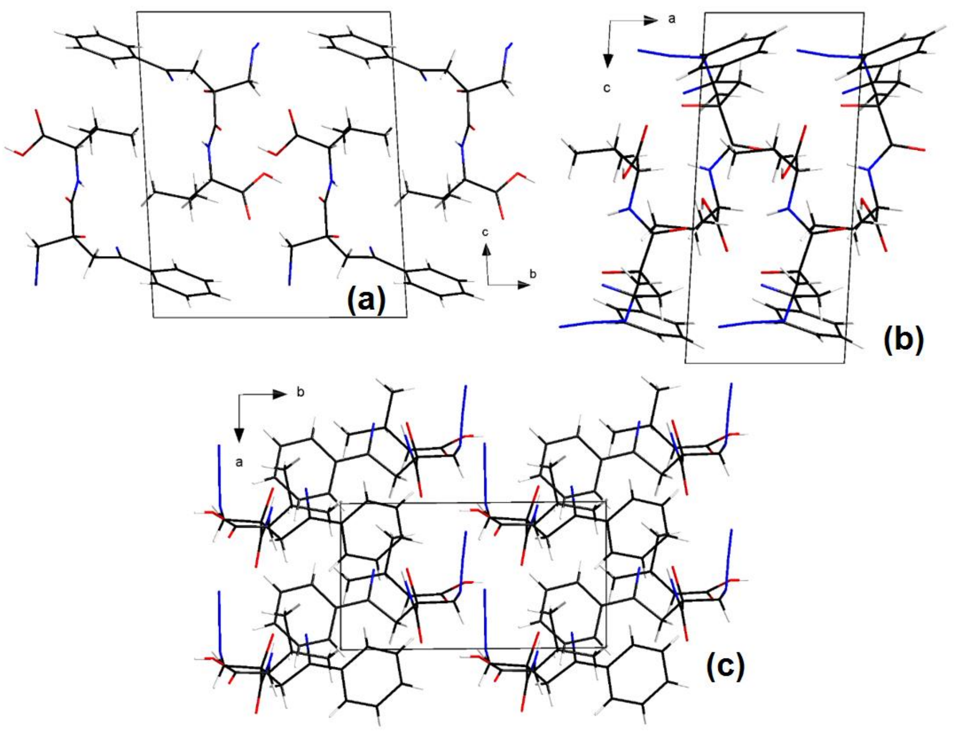

View along the *a* (a), *b* (b) and *c* (c) cell axes. Colour code as in Figure S18.

Figure S20: O–H···O hydrogen bond motif along axis a.

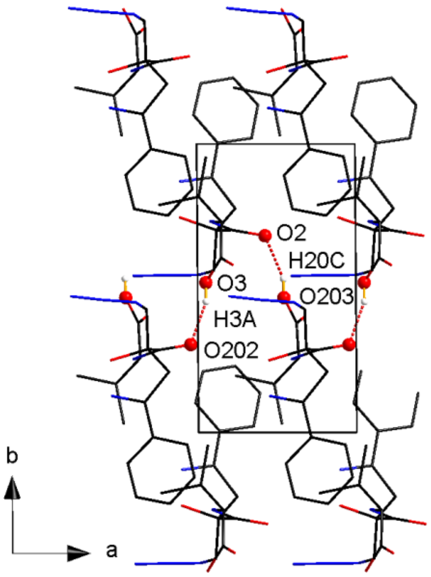

Same colour code as in Figures S18-S19. Nonessential hydrogen atoms were omitted for clarity.

Table S1: [1]H and ^{13}C chemical shifts of 3.6 mM compound 1 in 20 mM PB/10% D_2O at pH 5.1 and at 283K, related to Figures 4.

Residue	δ NH (ppm)	δ Hα (ppm)<br>^3J (Hz)<br>C_α	δ Hβ (ppm)<br>C_β	δ and other protons (ppm) and C_δ	^{13}C δ
**Val-1**	NH_2 (97) : ---	CH_α (95) : 3.54<br>d, J = 6.46 Hz<br>58.26	CH_β (96) : 1.93	$CH_{3\gamma}$ (116) : 0.73<br>$CH_{3\gamma}$ (116) : 0.73	CO (86) : --
**Gln-2**	NH (85) : 8.54<br>NH_2 (114) : ND	CH_α (84): 4.16<br>52.76	$CH_{2\beta}$ (94) : 1.72<br>36.0	$CH_{2\gamma}$ (112) : 2.05	CO (83) : --<br>*CO (113)* : --
**Ile-3**	NH (82) : 8.09	CH_α (81) : 3.71<br>58.03	$CH_{2\beta}$ (92) : 1.43	$CH_{3\gamma}$ (111) : 0.89<br>$CH_{2\gamma}$ (110) : 1.18<br>$CH_{3\delta}$ (109) : 0.55	CO (80) : --
**Ile-4**	NH (79) : 8.31	CH_α (78) : 3.71	$CH_{2\beta}$ (90) : 1.45	$CH_{3\gamma}$ (107) : 0.55<br>$CH_{2\gamma}$ (106) : 0.89<br>CH_{3d} (108) : 0.55	CO (77) : --
**Asn-5**	NH_2 (76) : 8.27<br>NH (105) : ND	CH_α (75): 4.35<br>49.94	$CH_{2\beta}$ (87) : 2.31 / 2.35<br>36.0	---	CO (74) : --<br>*CO (103)* : --
**Lys-6**	NH (72) : 8.09<br>NH_2 (102) : 7.28	CH_α (70) : 3.92	$CH_{2\beta}$ (73) : 1.35	$CH_{2\gamma}$ (99) : 1.02 /<br>$CH_{2\delta}$ (100) : 1.22<br>$CH_{2\varepsilon}$ (101) : 2.48 /<br>38.87	CO (69) : --
**Isox-7**	NH (68) : 8.28	--	$CH_{2\beta}$ (13) : 3.40 / 3.73 / 42<br>$CH_{2\beta}$ (9) : 3.31 / 3.45 / 41	5 CH^{Ar} (1-6) : 7.45 – 7.38 (m, 2H) / 7.28 – 7.24 (m, 3H) / 131.0, 127.64, 126.89	CO (12) : --<br>C (7) : --<br>C (10) : --
**Val-8**	NH (14) : 8.02	CH_α (16) : 3.82 / 59.63	CH_β (17) : 1.89	$CH_{3\gamma}$ (46) : 0.65 /<br>$CH_{3\gamma}$ (47) : 0.7 /	CO (18) : --
**Gln-9**	NH (19) : 8.40<br>NH_2 (66) : ND	CH_α (21): 4.02 / 52.62	$CH_{2\beta}$ (22) : 1.61	$CH_{2\gamma}$ (57) : 1.91	CO (23) : --<br>*CO (65)* : --
**Ile-10**	NH (24) : 8.07	CH_α (26) : 3.70<br>58.01	$CH_{2\beta}$ (27) : 1.42	$CH_{3\gamma}$ (55) : 0.52 /<br>$CH_{2\gamma}$ (54) : 0.80 /<br>$CH_{3\delta}$ (56) : 0.37 /	CO (28) : ---
**Val-11**	NH (29) : 7.93	CH_α (31) : 3.77<br>59.1	CH_β (40) : 1.65	$CH_{3\gamma}$ (52) : 0.58 /<br>$CH_{3\gamma}$ (53) : 0.58 /	CO (32) : --
**Tyr-12**	NH (33) : 8.33	CH_α (34): 4.27 / 55.06	$CH_{2\beta}$ (42) : 2.65	CH^{Ar}(63) : 6.85 /<br>130.4<br>CH^{Ar}(62) : 6.53 /<br>115.5	CO (35) : --
**Lys-13**	NH (36) : 8.16<br>NH_2 (51) : 7.33<br>$CONH_2$ (31) : ND	CH_α (37) : 3.93	$CH_{2\beta}$ (44) : 1.48	$CH_{2\gamma}$ (48) : 1.07 /<br>$CH_{2\delta}$ (49) : 1.37 /<br>$CH_{2\varepsilon}$ (50) : 2.68 /<br>39.16	CO (38) : --

Table S2: ^{1}H and ^{13}C chemical shifts of 3.6 mM compound 2 in 20 mM PB/10% D_2O at pH 5.1 and at 283K, related to Figures 5.

Residue	δ NH (ppm)	δ Hα (ppm) ^3J (Hz) $C_α$	δ Hβ (ppm) $C_β$	δ and other protons (ppm) and C $_δ$	^{13}C δ
**Val-1**	NH_2 (97) : ---	$CH_α$ (95) : 3.56 d, J = 6.46 Hz	$CH_β$ (96) : 1.93	$CH_{3γ}$ (116) : 0.77 / $CH_{3γ}$ (116) : 0.77 /	CO (86) : --
**Gln-2**	NH (85) : 8.55 NH_2 (114) : ND	$CH_α$ (84): 4.17	$CH_{2β}$ (94) : 1.72	$CH_{2γ}$ (112) : 2.06	CO (83) : -- *CO (113)* : --
**Ile-3**	NH (82) : 8.36	$CH_α$ (81) : 3.91	$CH_{2β}$ (92) : 1.53	$CH_{3γ}$ (111) : 0.90 / $CH_{2γ}$ (110) : 1.22 / $CH_{3δ}$ (109) : 0.57 /	CO (80) : --
**Ile-4**	NH (79) : 8.22	$CH_α$ (78) : 3.85	$CH_{2β}$ (90) : 1.51	$CH_{3γ}$ (107) : 0.87 / $CH_{2γ}$ (106) : 0.1.16 / CH_{3d} (108) : 0.55 /	CO (77) : --
**Asn-5**	NH_2 (76) : 8.28 NH (105) : ND	$CH_α$ (75): 4.33	$CH_{2β}$ (87) : 2.26	---	CO (74) : -- *CO (103)* : --
**Lys-6**	NH (72) : 8.15 NH_2 (102) : 7.28	$CH_α$ (70) : 3.93	$CH_{2β}$ (73) : 1.48	$CH_{2γ}$ (99) : 1.05 / $CH_{2δ}$ (100) : 1.27 / $CH_{2ε}$ (101) : 2.55 /	CO (69) : --
**Isox-7**	NH (68) : 8.28	--	$CH_{2β}$ (13) : 3.45 / 3.63 $CH_{2β}$ (9) : 3.37 / 3.53 /	5 CH^{Ar} (1-6) : 7.45 – 7.38 (m, 2H) / 7.28 – 7.24 (m, 3H) /	CO (12) : -- C (7) : -- C (10) : --
**Val-8**	NH (14) : 8.02	$CH_α$ (16) : 3.86	$CH_β$ (17) : 1.82	$CH_{3γ}$ (46) : 0.60 / $CH_{3γ}$ (47) : 0.60 /	CO (18) : --
**Gln-9**	NH (19) : 8.43 NH_2 (66) : ND	$CH_α$ (21): 4.15	$CH_{2β}$ (22) : 1.72	$CH_{2γ}$ (57) : 2.068	CO (23) : -- *CO (65)* : --
**Ile-10**	NH (24) : 8.23	$CH_α$ (26) : 3.85	$CH_{2β}$ (27) : 1.52	$CH_{3γ}$ (55) : 0.55 / $CH_{2γ}$ (54) : 1.16 / 0.86 / $CH_{3δ}$ (56) : 0.53 /	CO (28) : --
**Val-11**	NH (29) : 8.06	$CH_α$ (31) : 3.82	$CH_β$ (40) : 1.67	$CH_{3γ}$ (52) : 0.6 / $CH_{3γ}$ (53) : 0.6 /	CO (32) : --
**Tyr-12**	NH (33) : 8.39	$CH_α$ (34): 4.30	$CH_{2β}$ (42) : 2.67 / 2.72	CH^{Ar}(63) : 6.87 / CH^{Ar}(62) : 6.54 /	CO (35) : --
**Lys-13**	NH (36) : 8.17 NH_2 (51) : 7.31 $CONH_2$ (31) : ND	$CH_α$ (37) : 3.93	$CH_{2β}$ (44) : 1.52	$CH_{2γ}$ (48) : 1.09 // $CH_{2δ}$ (49) : 1.39 / $CH_{2ε}$ (50) : 2.70 / 39.16	CO (38) : --

Table S3: [1]H and ^{13}C chemical shifts of 3.6 mM compound 3 in 20 mM PB/10% D_2O at pH 5.1 and at 283K.

Residue	δ NH (ppm)	δ Hα (ppm) ^3J (Hz) $C_α$	δ Hβ (ppm) $C_β$	δ and other protons (ppm) and C δ	[13]C δ
**Lys-1**	NH_2 (29) : --- NH_2 (132) : 7.51	CHα (95): 4.00 54.70	$CH_{2β}$ (98) : 1.82	$CH_{2γ}$ (116) : 1.33 $CH_{2δ}$ (130) : 1.62 $CH_{2ε}$ (131) : 2.92	CO (86) : --
**Val-2**	NH (85) : 8.56	CHα (84) : 4.09	CHβ (94) : 1.92	$CH_{3γ}$ (112) : 0.83 $CH_{3γ}$ (129) : 0.88	CO (83) : --
**Val-3**	NH (82) : 8.40	CHα (82) : 4.05	CHβ (92) : 1.92	$CH_{3γ}$ (109) : 0.80 $CH_{3γ}$ (110) : 0.86	CO (80) : --
**Val-4**	NH (79) : 8.31	CHα (78) : 3.98	CHβ (90) : 1.88	$CH_{3γ}$ (106) : 0.75 $CH_{3γ}$ (107) : 0.82	CO (77) : --
**Ile-5**	NH (76) : 8.15	CHα (75) : 4.10	CHβ (125) : 1.58	$CH_{3γ}$ (127) : 0.68 $CH_{2γ}$ (126) : 1.30 $CH_{3δ}$ (128) : 1.01	CO (28) : --
**Thr-6**	NH (72) : 8.17	CHα (70): 4.17	CHβ (73) : 4.01	$CH_{3γ}$ (99) : 1.06	CO (69) : --
**Isox-7**	NH (68) : 8.43	--	$CH_{2β}$ (13) : 3.68 / 3.88 $CH_{2β}$ (9) : 3.353/ 3.73	5 CH^{Ar} (1-6) : 7.68 – 7.70 (m, 2H) / 7.49 – 7.41 (m, 3H) / 133.4, 130.9, 128.9,	CO (12) : -- C (7) : -- C (10) : --
**Thr-8**	NH (14) : 8.18	CHα (16): 4.25	CHβ (17) : 4.15	$CH_{3γ}$ (46) : 1.06	CO (18) : --
**Lys-9**	NH (19) : 8.50 NH_2 (118) : 7.49	CHα (21): 4.28	$CH_{2β}$ (22) : 1.73	$CH_{2γ}$ (57) : 1.38 $CH_{2δ}$ (65) : 1.62 $CH_{2ε}$ (66) : 1.92	CO (23) : --
**Val-10**	NH (24) : 8.18	CHα (26) : 3.98	CHβ (27) : 1.92	$CH_{3γ}$ (55) : 0.80 $CH_{3γ}$ (119) : 0.87	CO (28) : --
**Ile-11**	NH (29) : 8.25	CHα (31) : 4.04	CHβ (40) : 1.74	$CH_{3γ}$ (52) : 1.08 $CH_{2γ}$ (53) : 1.39 $CH_{3δ}$ (120) : 0.77	CO (32) : --
**Leu-12**	NH (33) : 8.34	CHα (34) : 4.26	$CH_{2β}$ (42) : 1.40 / 1.46	$CH_{2γ}$ (58) : 1.53 / 0.86 $CH_{3δ}$ (121) : 0.79 $CH_{3δ}$ (63) : 0.79	CO (35) : --
**His-13**	NH (36) : 8.47 $CONH_2$ (39) : ND	CHα (37): 4.59	$CH_{2β}$ (44) : 3.10 / 3.22	CH^{Ar}(123) : 7.24 CH^{Ar}(122) : 8.55	CO (38) : --

Table S4: Symmetry-independent intermolecular hydrogen bonds in compound **11b**. Estimated standard deviations are reported in brakets.

O–H⋯O	$d_{O–H}$ / Å	$d_{H\cdots O}$ / Å	$d_{O\cdots O}$ / Å	α_{OHO} / deg	Symmetry
O3–H3A⋯O202	0.82	1.92	2.715(1)	162	-1+x, y, z
O203–H20C⋯O2	0.82	1.92	2.700(1)	160	x, y, z
